# Downregulation of dermatopontin in cholangiocarcinoma cells suppresses CCL19 secretion of macrophages and immune infiltration

**DOI:** 10.1007/s00432-023-05532-1

**Published:** 2024-02-01

**Authors:** Peng Xu, Siyang Li, Ke Liu, Rui Fan, Fahui Liu, Haoxuan Zhang, Donghua Liu, Dongyan Shen

**Affiliations:** Xiamen Cell Therapy Research Center, The First Affiliated Hospital of Xiamen University, School of Medicine, Xiamen University, No. 55 Zhenhai Road, Xiamen, 361003 Fujian Province China

**Keywords:** Cholangiocarcinoma, DPT, Tumor microenvironment, Immune infiltration, Macrophages, CCL19

## Abstract

**Objective:**

The tumor microenvironment (TME) in cholangiocarcinoma (CHOL) is typically characterized by a low level of immune infiltration, which accounts for the dismal prognosis of this patient population. This study sought to investigate the mechanisms underlying the reduced infiltration of immune cells into the CHOL TME.

**Methods:**

We constructed a Least Absolute Shrinkage and Selection Operator (LASSO) regression model to identify prognosis-related differentially expressed genes (DEGs). The 'Corrplot' package was employed to analyze the correlation between dermatopontin (DPT) and immune infiltration in CHOL. The Tumor and Immune System Interaction Database (TISIDB) was used to evaluate the association between DPT and immunology. Single-cell analysis was conducted to localize CCL19 secretions. Western blot and qPCR were utilized to detect DPT expression, while immunofluorescence was performed to investigate the cellular localization of DPT. Additionally, ELISA analysis was employed to assess the alteration in CCL19 secretion in cancer-associated fibroblasts (CAFs) and macrophages.

**Results:**

Our findings revealed that CHOL patients with low DPT expression had a poorer prognosis. Enrichment analysis demonstrated a positive correlation between DPT levels and the infiltration of immunomodulators and immune cells. Moreover, high DPT levels were associated with enhanced anti-PD-1/PD-L1 immunotherapeutic responses. Furthermore, DPT expression impacted the landscape of gene mutations, showing a negative association with tumor grade, stage, and lymph node metastasis. Based on the results of protein peptides analysis and cell experiments, it was inferred that the downregulation of DPT in CHOL cells effectively suppressed the secretion of CCL19 in macrophages.

**Conclusions:**

DPT is a novel prognosis-related biomarker for CHOL patients, and this study provides preliminary insights into the mechanism by which DPT promotes the infiltration of immune cells into the CHOL TME.

**Supplementary Information:**

The online version contains supplementary material available at 10.1007/s00432-023-05532-1.

## Introduction

Cholangiocyte differentiation is a distinctive feature seen in cholangiocarcinoma (CHOL), an epithelial cancer that originates within the bile ducts (de Groen et al. 1999). CHOL ranks as the second most frequent primary hepatic cancer and epidemiological studies have indicated a growing disease incidence in the West (Welzel et al. 2006). Advanced CHOL has a dismal prognosis, with a median survival of only 24 months (Farley et al. 1995). Unfortunately, the majority of patients receive a late-stage diagnosis for which no effective cure is available. Current treatment approaches are largely limited to symptom relief, extending survival, and enhancing the quality of life. Therefore, there is an urgent need for more effective methods to identify and treat CHOL.

Dermatopontin (DPT), a non-collagenous extracellular matrix (ECM) protein initially identified in the dermis, has been documented in various tissues, including the bile duct (Okamoto and Fujiwara 2006). Current evidence suggests that DPT regulates the ECM architecture and modifies the interaction between decorin and transforming growth factor-beta (TGF-β) (MacBeath et al. 1993; Takeda et al. 2002). Furthermore, DPT shows strong associations with cancer. For example, it has been observed that DPT exhibits a potent ability to inhibit the invasion and migration of cancer cells in liver and oral cancers (Fu et al. 2014; Yamatoji et al. 2012). Additionally, multiple signaling pathways linked to cancer progression and prognosis, such as Wnt/β-catenin, Hippo/YAP, TGF-β, and ERK/MAPK pathways, have also been correlated with DPT expression (Catalán et al. 2022; Guo et al. 2019; Liu et al. 2020; Ye et al. 2023). Nonetheless, the biological roles and underlying mechanisms of DPT in CHOL remain incompletely understood.

Chemokine (C–C motif) ligand 19, also known as CCL19, is a chemotactic factor belonging to the C–C chemokine family. CCL19 primarily interacts with its specific receptor, CCR7 (C–C chemokine receptor 7), and plays a vital role in immune cell chemotaxis and localization. In recent years, researchers have increasingly focused on its role in cancer. On one hand, studies have documented downregulated CCL19 expression in patients with colorectal and gastric cancers, with high CCL19 levels effectively inhibiting proliferation and migration of these cancer cells (Xu et al. 2018; Zhou et al. 2020). On the other hand, research has indicated that CCL19 secreted by cancer-associated fibroblasts (CAFs) promotes CD8^+^ T cell infiltration into the tumor microenvironment (TME) in lung cancer, leading to tumor growth inhibition (Cheng et al. 2018). Furthermore, CAR-T cells with high CCL19 secretion have been reported to enhance immune cell infiltration into the TME and improve CAR-T therapy efficacy in patients (Pang et al. 2021). Therefore, it is crucial to investigate whether CCL19 serves a similar function in CHOL and identify its potential upstream regulators.

Overall, this study revealed that reduced expression of the immune-related biomarker DPT in CHOL cells may diminish the infiltration of various immune cytotoxic cells into the TME, such as CD8^+^ T cells and NK cells. Numerous studies have indicated a positive correlation between immune cell infiltration and cancer patient prognosis (Salgado et al. 2015; Wang et al. 2020). Moreover, cancer patients with high DPT expression levels tend to exhibit a better response and prognosis with anti-PD-1/PD-L1 immunotherapy. Finally, through integrated bioinformatics analysis and cellular experiments, it was uncovered that DPT secreted by CHOL cells could directly stimulate the secretion of the chemokine CCL19 by macrophages, potentially elucidating the mechanism by which DPT elevates immune cell infiltration levels in the TME.

## Materials and methods

### Data sources

The transcriptome profiles and clinical data for TCGA-CHOL were obtained from the University of California, Santa Cruz (UCSC) Xena database. Additionally, we accessed three CHOL datasets from the Gene Expression Omnibus (GEO) database by searching for the keywords GSE26566, GSE45001, and GSE138709. Prior to preprocessing, which included background correction, normalization, and expression calculation, we harmonized the probes to align with the official gene symbols listed in the annotation data provided by the respective platforms.

### Analysis of differentially expressed genes

DEGs were examined using the R software's limma tools with GSE26566 and GSE45001 datasets (|log_2_(FC)|> 1, *p* < 0.05). In the GSE26566 dataset, the surrounding liver group was excluded and only the normal bile duct and CHOL groups were retained. Applying the “DESeq2”, “limma”, and “edgeR” tools, DEGs in the TCGA-CHOL samples were screened. The VennDiagram package was used to display the intersected DEGs in these three datasets.

### Construction of Lasso Regression Model

The least absolute contraction and selection operator (LASSO) regression analysis on the intersected genes linked to overall survival (OS) in the aforementioned three sets of data was carried out to narrow down prognostic relevant genes using the glmnet R software package in The Cancer Genome Atlas (TCGA) database. The Akaike Information Criterion (AIC) approach was then employed to linearly integrate the regression coefficients from the multivariate Cox regression analysis with the levels of expression of a subset of genes associated with survival. The optimal survival-related risk model was generated, and the following formula was used to calculate the risk score:

$$\mathrm{Riskscore }= \sum_{i=1}^{n}{\text{Coef}}i\times \mathrm{ Exp}i$$

where Expi is the expression value of the survival-related genes and Coefi is the corresponding regression coefficient calculated by multivariate Cox regression analysis. TCGA data were used as the training cohort.

### Survival and prognostic analysis

Using the R packages Survival and survminer, Kaplan–Meier analysis of CHOL patients in low and high-risk groups was carried out. To assess the efficacy of the risk signatures in predicting the outcomes of CHOL patients, time-dependent receiver operating characteristic (ROC) curves were generated using the R package survivalROC. The area under the ROC curve’s (AUC) size indicates how well a risk model can predict outcomes. Using the Pheatmap R package, a risk plot was generated to display the distribution of samples’ survival status across several prognostic groups. The analysis of the risk-related gene's mRNA expression in CHOL was conducted using GEO datasets.

### Enrichment analysis of GO and KEGG

To assess the mRNA expression level of DPT in CHOL patients and normal tissue samples, data from both TCGA and GEO were collected. The cohort was then divided into high and low DPT expression groups based on the median DPT expression value. A heatmap was used to visualize genes that showed strong positive or negative associations with DPT expression. Furthermore, the Kyoto Encyclopedia of Genes and Genomes (KEGG) pathways and Gene Ontology (GO) were employed for exploring biological processes and detecting biological functions. To further validate the KEGG pathways and GO processes related to the signature, GO and KEGG analyses for the differentially expressed genes were conducted using the R package clusterProfiler.

### Correlation analysis of DPT and immune infiltration in the tumor microenvironment of CHOL

We conducted correlation analysis to examine the association between DPT expression and immune cell infiltration in CHOL tumor samples by utilizing data from TCGA databases. The assessment was carried out using the “corrplot” package in R and the Timer2.0 bioinformatics website (http://timer.comp-genomics.org/timer/). Statistical significance was determined with a *p* value less than 0.05.

### Analysis of tumor mutation and genomic alterations

We used the camoip database (https://www.camoip.net/) to perform an analysis and visualization of variations in mutated genes between the high- and low-DPT groups. Furthermore, we examined the likelihood and characteristics of DPT mutations in CHOL samples using the cBioPortal database (https://www.cbioportal.org/). The ULACAN database (https://ualcan.path.uab.edu/index.html) was used to further investigate the effect of DPT expression on the clinical grade, stage and metastasis of CHOL and LIHC.

### Analysis of downstream target genes and PPI network of DPT

We harnessed the GeneMANIA online database (https://genemania.org/) to investigate the association between DPT and DEGs and subsequently built an interaction network where nodes were associated with a significance level of less than 0.05.

### Cell culture and lentivirus infection

We acquired three bile duct cancer cell lines (QBC939, HUCCT1, RBE) and human monocyte cell lines (THP-1) from Qingqi (Shanghai) Biotechnology Development Co., Ltd. Cancer-associated fibroblasts were isolated from clinical CHOL tissue obtained from patients who underwent surgery. CAFs were validated through flow cytometry, confirming the expression of α-SMA, FAP, and FSP1. RBE and HUCCT1 cell lines were cultured in DMEM medium (BasalMedia, Shanghai, China), while QBC939 was cultured in 1640 medium (BasalMedia, Shanghai, China). These media were supplemented with 10% fetal bovine serum (Cegrogen biotech, Germany) and 0.1% penicillin and streptomycin (BasalMedia, Shanghai, China). The cells were maintained at 37 °C in a humidified 5% CO_2_ incubator. THP-1 cells were treated with 50 ng/mL 12-myristate 13-acetate (PMA) (MCE, NJ, USA) for 72 h, leading to their differentiation into macrophages, which were subsequently used in further experiments. To generate lentivirus, we obtained plvx-puro-DPT overexpressing plasmids, empty control plasmids, and packaging plasmids psPAX2 and pMD2.G from MiaoLing Plasmid Platform. These plasmids were transfected into 293 T cells using Lipofectamine 3000 (cat. L3000008, ThermoFisher) as per the manufacturer's instructions. QBC939, HUCCT1, and RBE cell lines were infected with the corresponding lentivirus for 72 h and then subjected to puromycin selection (1 μg/mL) for 48 h to establish stable DPT overexpression cell lines.

### Immunohistochemistry

First, the paraffin-embedded sections were cut into 4 μm thick slices and baked for 4–6 h at 60 °C. Subsequently, the sections were dewaxed in a clearing agent, followed by a series of alcohol washes (100%, 95%, and 75%), rinsed with distilled water, and subjected to antigen retrieval in boiling water using citric acid tissue antigen retrieval solution (MXB Biotechnologies, Fujian, China). They were then blocked with peroxidase and 10% BSA and incubated overnight with murine anti-DPT antibody (1:100, Proteintech, Wuhan, China) at 4 °C. Afterward, the sections were washed with PBS three times, incubated with an HRP-conjugated secondary antibody (Sigma, St. Louis, Missouri, USA), and stained with DAB (MXB Biotechnologies, Fujian, China) and hematoxylin. Finally, the sections were dehydrated and sealed.

### RNA isolation and qRT-PCR

Total RNAs were extracted from tissues or cells using an RNAsimple Total RNA kit (TianGen, Beijing, China). Then, 1 µg of RNA was utilized to synthesize cDNAs with the FastKing RT Kit (with gDNase) (TianGen, Beijing, China). Real-time quantitative PCR was conducted using QuantStudio™ Real-Time PCR software in accordance with the manufacturer's protocol. The relative changes in gene expression were determined using the 2^−ΔΔCt^ method and normalized to the internal control GAPDH. The primer sequences used were as follows:

DPT forward: 5′-CTATATCCGAGGAGCAACAACC-3′;

reverse: 5′-TTCAGTCATCCGGCACATTAT-3′;

CCL19 forward: 5′-GGCACCAATGATGCTGAAGA-3′;

reverse: 5′-GCAGCCATCCTTGATGAGAAG-3′;

GAPDH forward: 5′-CCCTTCATTGACCTCAACTACA-3′;

reverse: 5′-ATGACAAGCTTCCCGTTCTC-3′.

### Protein extraction and western blot

The cells were harvested using RIPA lysis buffer (Solarbio, Beijing, China), which contains both protease inhibitors and phosphatase inhibitors. Protein extraction involved the use of an oscillation mixer to ensure thorough cell lysis at a temperature of 4 ºC for over 30 min. Subsequently, the protein concentration was determined and quantified using the BCA method. The proteins were first subjected to electrophoresis on a 10% polyacrylamide gel and then transferred onto a PVDF membrane that had a thickness of 0.22 μm. To prevent non-specific binding, a 5% milk solution was used for blocking, and the samples underwent three PBS washes. The specific primary antibody used was murine anti-DPT (1:1000, Proteintech, Wuhan, China), and it was incubated at 4 °C overnight. The secondary antibody employed was goat anti-rabbit IgG antibody (1:3000, MA, USA), and it was incubated at room temperature for 2 h. The final step involved imaging after adding an ECL (YanXi Biotechnology, Shanghai, China) reaction enhancement solution.

### Macrophages and CAFs co-cultured with CHOL cells

For the macrophage co-culture system, 5 × 10^5^ THP-1 cells were seeded onto 6-well plates and cultured with RPMI-1640 medium supplemented with 50 ng/mL PMA for 48 h. When THP-1 cells adhered to the surface of the plates, it indicated that THP-1 cells had differentiated into macrophages. Subsequently, the medium was replaced by RPMI-1640 medium with 10% fetal calf serum for 24 h. Finally, the medium of macrophages was changed to the conditioned medium (CM) of different CHOL cells. 24 h later, it was replaced with fresh medium, and at 48 h, the supernatant was collected for subsequent ELISA assay.

For the CAFs co-culture system, 3 × 10^5^ CAFs were seeded into 6-well plates for 24 h and the medium was replaced by the CM of different CHOL cells. The following steps were consistent with the co-culture of macrophages.

### ELISA assay

The standard product and the supernatant collected earlier were added to the wells of the perforated strip provided in the CCL19 ELISA kit (ABclonal, Wuhan, China). The mixture was then incubated at 37 °C for 2 h. Afterward, the plate was removed, and the liquid was discarded. The perforated strip was washed three times with a washing buffer. Subsequently, 100 μl of biotin-conjugated antibody was added to each well, followed by incubation at 37 °C for 1 h. The buffer in the perforated strip was then discarded, and the strip was washed three times with a washing solution. Next, 100 μl of the prepared streptavidin-avidin-HRP working solution was added, and incubation was carried out once more at 37 °C for 1 h. The perforated strip was then removed from the incubator and washed three times. Following this, 100 μl of TMB substrate was added, and incubation was continued for 20 min. Finally, 50 μl of termination solution was introduced, and the optical density (OD) value of each well was measured. The concentration of CCL19 in each well was calculated based on the standard product-derived curve.

### Immunofluorescence staining

Cells were first fixed with 4% paraformaldehyde, then treated with 0.25% Tritonx-100 to improve membrane permeability, and incubated with glycine for 5 min at room temperature. Subsequently, murine anti-DPT (1:100, Proteintech, Wuhan, China) was added for incubation at 4 °C overnight and then incubated with 488 Alexa Fluor secondary antibody (ThermoFisher, MA, USA) at room temperature for 1 h. Finally, nuclear dye DAPI was incubated for 2 min and observed by a confocal microscope (Leica, Wetzlar, Germany).

### Statistical analysis

GraphPad Prism 9 software was used to analyze all quantitative data. Data were represented as mean ± SEM calculated using GraphPad. The differences among groups were detected with a *t* test. The survival analyses were determined by the Kaplan–Meier curve, log-rank test, and Cox proportional hazard regression mode. Correlation analysis was conducted using Spearman’s test. In all analyses, a *p* value less than 0.05 was statistically significant. The use of asterisks (*, **, ***, ****) indicated significance levels, with *p* < 0.05, *p* < 0.01,* p* < 0.001, and *p* < 0.0001, respectively. Unless specified otherwise, all data were representative of at least three independent experiments.

## Results

### Screening the intersected DEGs of CHOL using GEO and TCGA databases

**Fig. 1:**
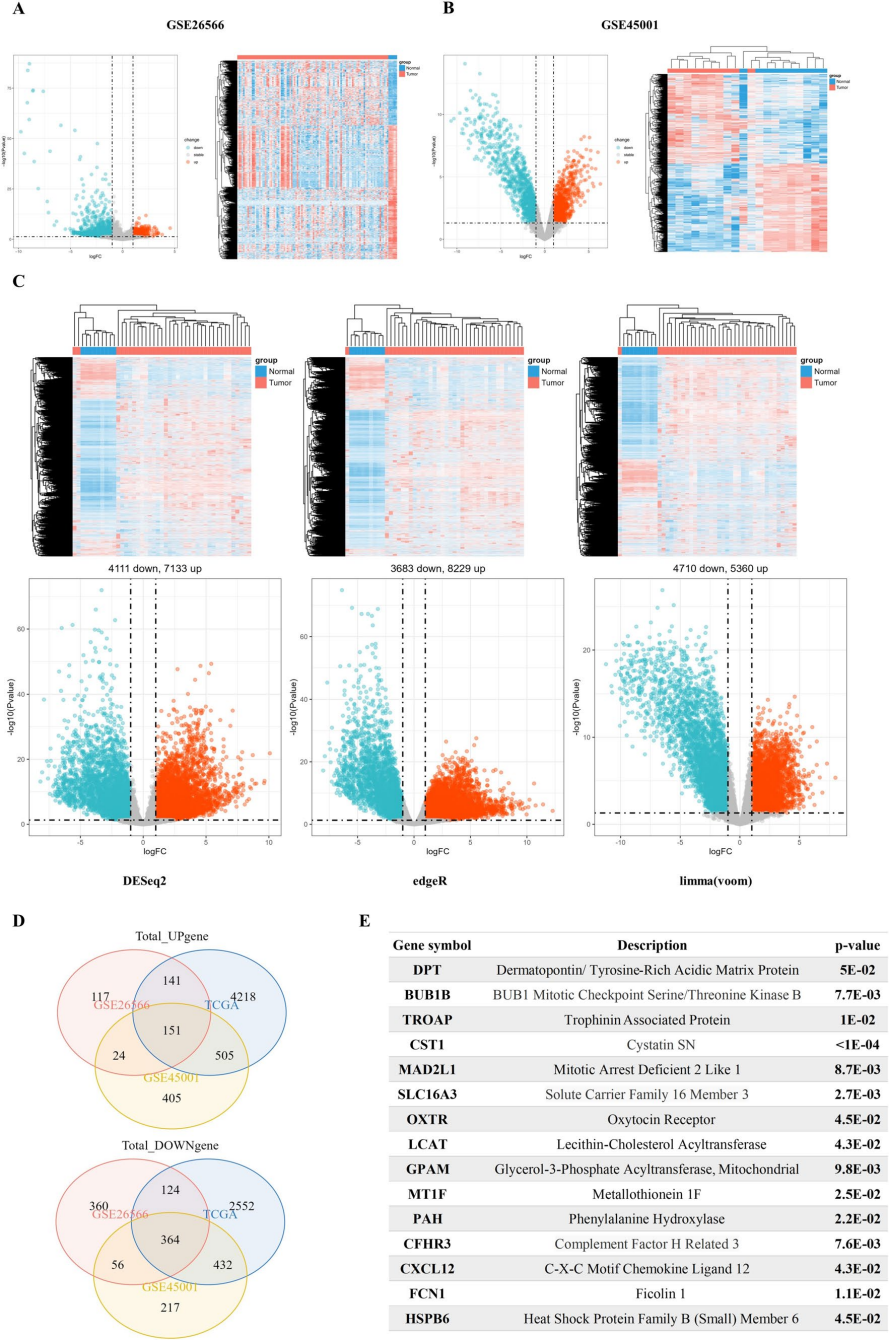
Screening of the intersected DEGs of CHOL. **A**–**B** Volcano plot (|log (FC)|> 1, *p* < 0.05) and heatmap plot of DEGs in GSE26566 and GSE45001 datasets. **C** Using DESeq2, edgeR, and limma algorithms, DEGs in CHOL from TCGA were visualized by volcano plot and heatmap plot. **D** Intersection of DEGs from GEO and TCGA database. **E** Overlapping DEGs and log-rank p genes

In the GSE26566 sample, a total of 433 upregulated genes and 904 downregulated genes were identified (|log_2_(FC)|> 1, *p* < 0.05). Similarly, in the GSE45001 sample, there were 1,069 downregulated genes and 1,085 upregulated genes (|log_2_(FC)|> 1, *p* < 0.05). Dimensionality reduction was performed using principal component analysis (PCA) (Supplementary Fig. 1A–C). Heatmaps and volcano maps were generated to visualize these differentially expressed genes (DEGs) (Fig. 1A, B). DEGs in TCGA samples were also examined using three R packages: DESeq2, edgeR, and limma. The results were displayed in heatmaps and volcano maps (Fig. 1C). By applying the criteria of |log_2_(FC)|> 1, *p* < 0.05, 5,015 upregulated genes and 3,472 downregulated genes were identified from TCGA datasets using DESeq2, edgeR, and limma algorithms (Supplementary Fig. 1D). The intersection of genes with differential expression in the GSE26566, GSE45001, and TCGA datasets yielded 151 upregulated genes and 364 downregulated genes (Fig. 1D). Finally, to identify genes affecting CHOL prognosis at *p* < 0.05, the log-rank p test was performed with TCGA-CHOL dataset, yielding 15 prognostic-related DEGs by intersecting these genes with the DEGs mentioned above (Fig. 1E).

### DPT is a prognostic CHOL biomarker

**Fig. 2:**
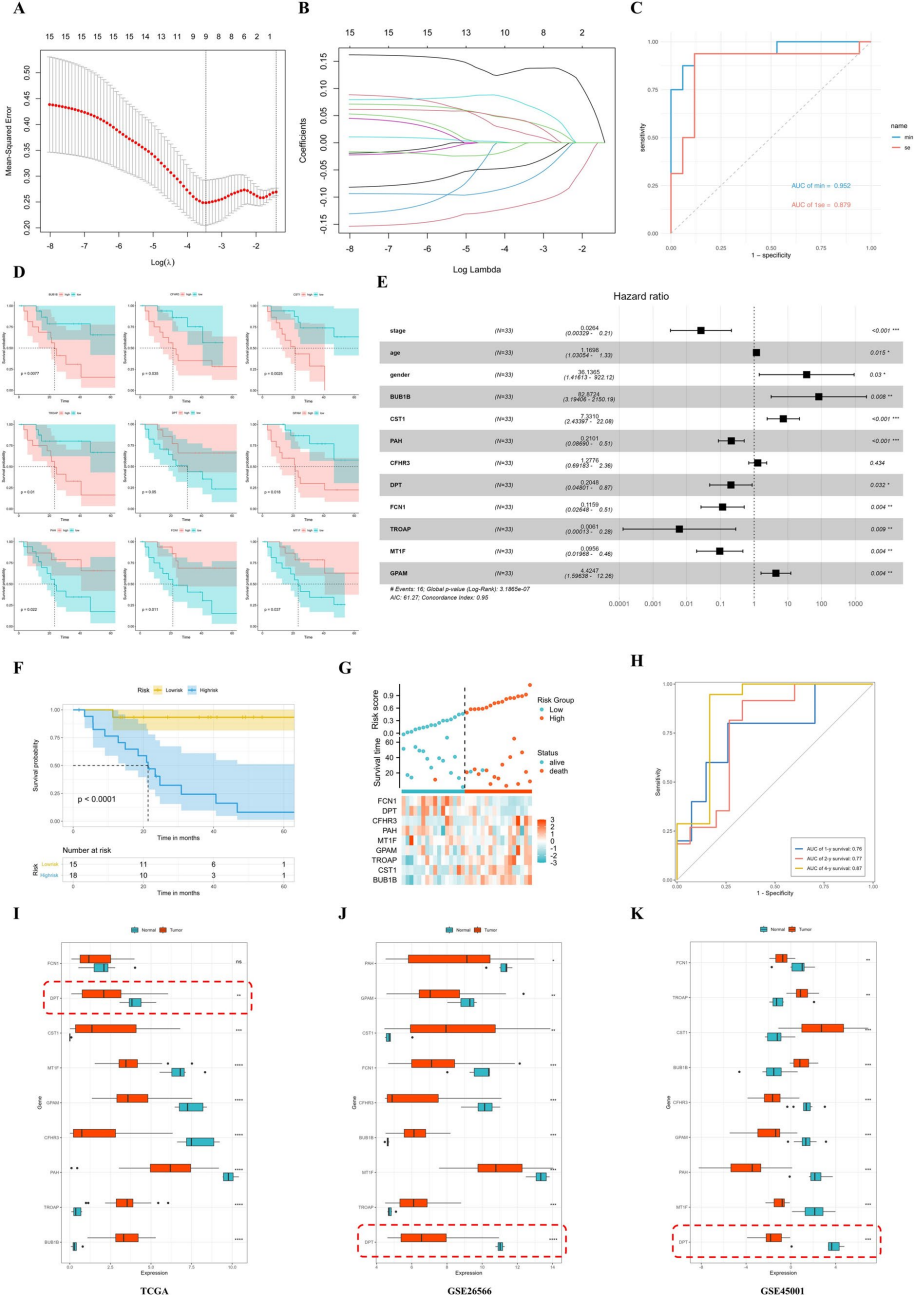
DPT is a favorable biomarker for the prognostic prediction of CHOL. **A** Cross-validation for tuning parameter screening in the LASSO regression model. **B** Coefficient profiles in the LASSO regression model. **C** ROC curve verified the validity of the model. **D** Kaplan–Meier analysis verified the clinical prognostic effect of the target genes. **E** Constructing a cox-forest model to predict overall survival risk and protective factors in patients with CHOL. **F** The prognosis values of the risk model in TCGA-CHOL database. **G** Distribution of risk score, survival time, and candidate genes in high- and low-risk groups. **H** ROC curve was used to demonstrate the survival rate at 1, 3, and 4 years, respectively. **I**–**K** The expression of these nine genes was displayed with GEO (GSE26566 and GSE45001) and TCGA datasets (*, *p* < 0.05; **, *p* < 0.01; ***, *p* < 0.001; ****, *p* < 0.0001)

To identify specific targets affecting CHOL prognosis, the 15 prognostic-related DEGs were used to construct a model through a lasso regression algorithm, calculating regression coefficients. Nine genes were identified as the most valuable candidates affecting CHOL patients’ prognosis (Fig. 2A–B). Subsequently, Kaplan–Meier analysis demonstrated significant correlations between the expression level of these 9 candidate genes in CHOL and survival outcomes, and all correlations were statistically significant when comparing high and low levels of gene expression (Fig. 2D). ROC curve analysis confirmed the model's diagnostic specificity and sensitivity, with area under the ROC curve values for MIN and SE of 0.952 and 0.879, respectively (Fig. 2C). Additionally, a Cox-forest model was performed, revealing that age, gender, BUB1B, CST1, CFHR3, and GPAM were associated with an increased risk for CHOL survival (hazard ratios [HRs] > 1), while stage, PAH, DPT, FCN1, TROAP, and MT1F were protective factors (HRs < 1) (Fig. 2E). After sorting the TCGA samples into high- and low-risk groups based on the most appropriate cutoff value, Kaplan–Meier analysis showed that the low-risk group had a significantly better prognosis (*p* < 1 × 10^–4^) (Fig. 2F and Supplementary Fig. 2A). Furthermore, a heatmap was generated to visualize differences in the expression of several target genes between the high- and low-risk groups (Fig. 2G). Immune analysis of the risk model revealed significantly higher stromal and immune scores in the low-risk group (Supplementary Fig. 2B) and higher infiltration levels of CD8^+^ T cells, CD4^+^ T cells, and other immune cells in the low-risk group (Supplementary Fig. 2C). The AUCs for 1-, 2-, and 4-year OS in the TCGA dataset were 0.76, 0.77, and 0.87, respectively, indicating the model's clinical significance (Fig. 2H). Finally, the expression levels of these 9 genes were compared in normal bile duct and CHOL using GSE26566, GSE45001, and TCGA datasets. The findings indicated that DPT exhibited the most significant difference (Fig. 2I–K). Based on the literature (Fu et al. 2014; Zavvos et al. 2017), DPT was defined as the most valuable biomarker for CHOL prognosis.

### DPT potentially improves CHOL patients’ prognosis by elevating immune infiltration levels

**Fig. 3:**
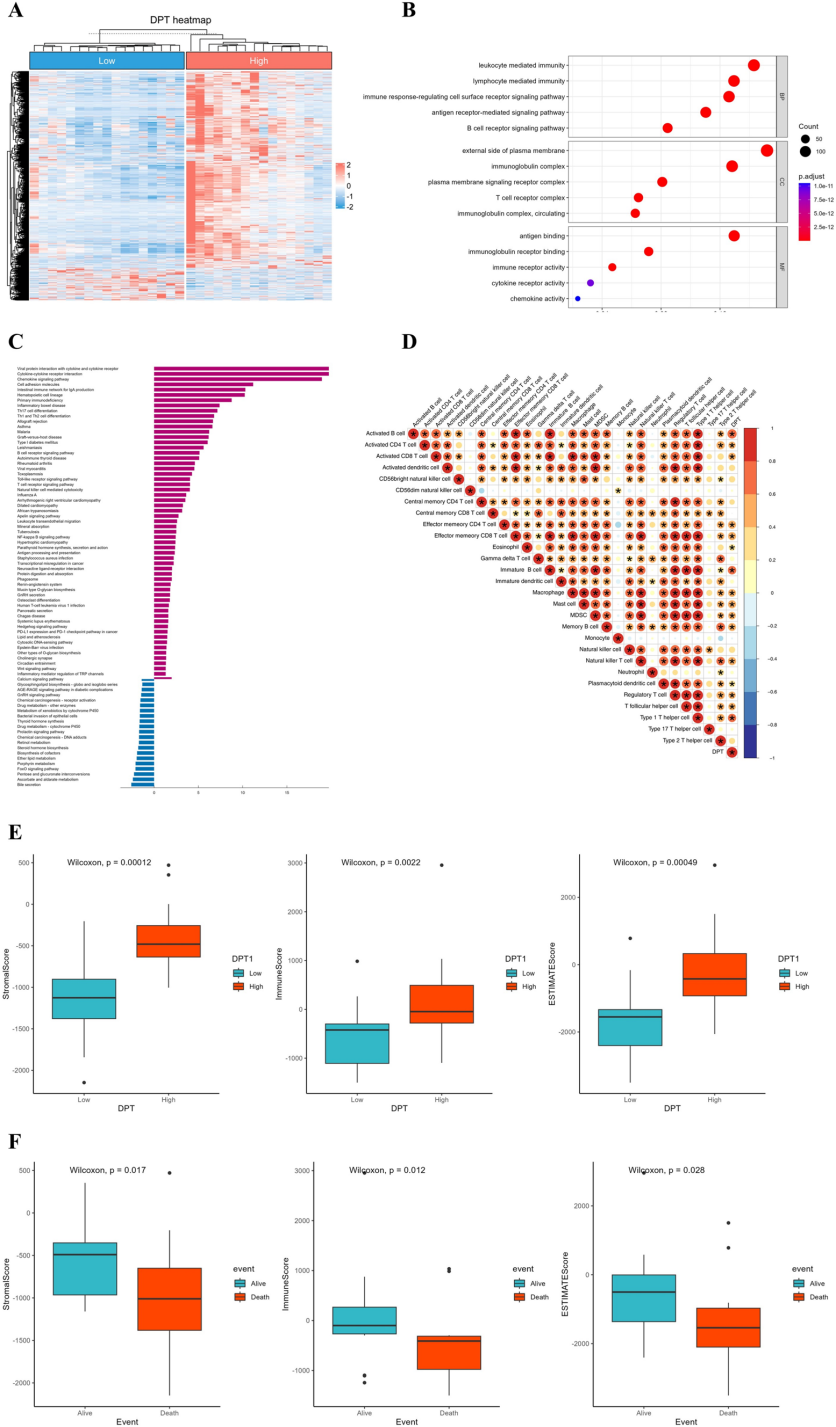
DPT can enhance the prognosis of CHOL patients by increasing immune infiltration. **A** Heatmap shows the DEGs of DPT low- and High-expression groups using TCGA-CHOL Dataset. **B**–**C** GO and KEGG analysis of DPT-related DEGs. **D** Analysis of the correlation between DPT and the level of immune cell infiltration. **E** Assessment of the associations between DPT and scores (stromal score, immune score, and estimate score) with the ESTIMATE algorithm. **F** Investigation of the relationship between scores (stromal score, immune score, and estimate score) and CHOL patients’ prognosis

To explore the biological function of DPT, TCGA-CHOL samples were divided into high- and low-expression groups based on the median DPT expression, and the DEGs were visualized in a heatmap (Fig. 3A). These DEGs were subjected to GO analysis, which revealed a strong association between DPT and leukocyte-mediated immunity, lymphocyte-mediated immunity, and other immune-related functions (Fig. 3B). KEGG analysis indicated that the top 5 signaling pathways enriched in the high-expression of DPT were viral protein interaction with cytokines and cytokine receptors, cytokines-cytokine receptors interaction, the chemokine signaling pathway, cell adhesion molecules, and the intestinal immune network for IgA production (Fig. 3C). Correlation analysis using the 'corrplot' package and Timer 2.0 showed that most types of immune cells, especially CD8^+^ T cells, CD4^+^ T cells, and M1 macrophages, were strongly positively correlated with DPT expression (Fig. 3D and Supplementary Fig. 3A–B). Furthermore, the relationship between DPT expression and immune/stromal scores was assessed. The results indicated that DPT expression was positively correlated with both scores, suggesting that DPT is closely related to immune cells and stromal cells in the TME (Fig. 3E). Higher immune and stromal scores were associated with better prognosis (Fig. 3F). Collectively, these findings suggested that DPT is an immune-related marker and may improve CHOL patient prognosis by enhancing immune cell infiltration.

### DPT is closely related to the immune regulatory molecules

**Fig. 4:**
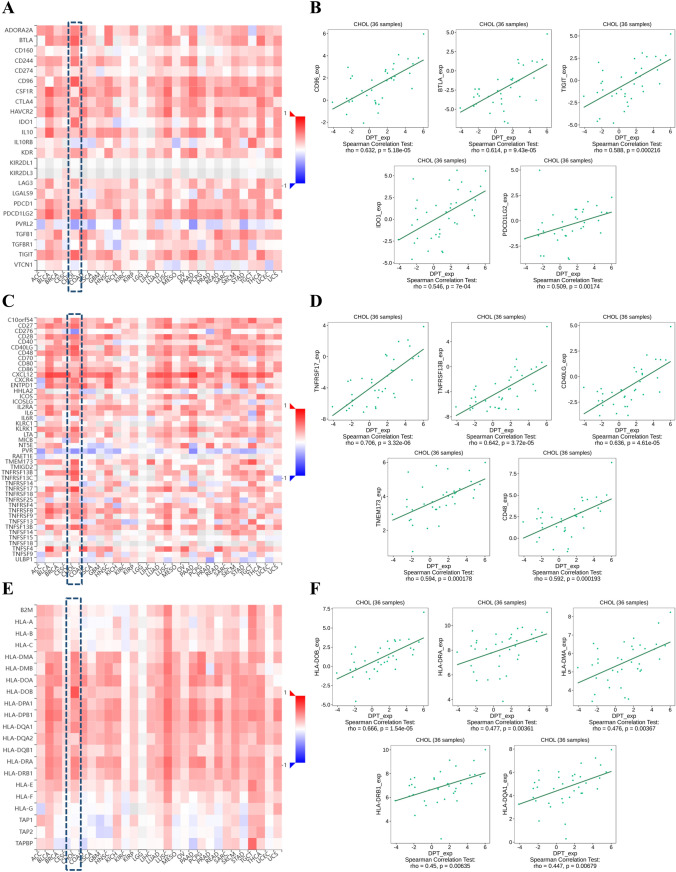
Relationship between DPT and immunoinhibitors, immunostimulators, and MHC molecules. **A–B** TISIDB was used to investigate the correlation between DPT expression and immunoinhibitors in CHOL. **C–D** TISIDB was used to investigate the correlation between DPT expression and immunostimulators in CHOL. **E–F** TISIDB was used to investigate the correlation between DPT expression and MHC molecules in CHOL

TISIDB analysis revealed the association between DPT and immunoinhibitors, immunostimulators, and Major Histocompatibility Complex (MHCs) in the TCGA dataset. Our results indicated a positive correlation between DPT expression in CHOL and most immunoinhibitors. The top 5 positively correlated immunoinhibitors were CD96 (rho = 0.632, *p* = 5.18e-05), BTLA (rho = 0.614, *p* = 9.34 × 10^–5^), TIGIT (rho = 0.588, *p* = 2.16 × 10^–4^), IDO1 (rho = 0.546, *p* = 7 × 10^–4^), and PDCD1LG2 (rho = 0.509, *p* = 1.74 × 10^–3^) (Fig. 4A–B). We also used a string diagram to visualize the interaction between DPT and classical immune checkpoint molecules such as PD-1, PD-L2, and CTLA4 (Supplementary Fig. 4A). Interestingly, our findings also revealed a strong positive correlation between DPT and several immunostimulators, including TNFRSF17 (rho = 0.706, *p* = 3.32 × 10^–6^), TNFRSF13B (rho = 0.642, *p* = 3.72 × 10^–5^), CD40LG (rho = 0.636, *p* = 4.61 × 10^–5^), TMEM173 (rho = 0.594, *p* = 1.78 × 10^–4^), and CD48 (rho = 0.592, *p* = 1.93 × 10^–4^) (Fig. 4C–D). Furthermore, most MHC molecules exhibited a significant positive correlation with DPT expression in CHOL samples, with the top 5 being HLA-DOB (rho = 0.666, *p* = 1.54 × 10^–5^), HLA-DRA (rho = 0.477, *p* = 3.61 × 10^–3^), HLA-DMA (rho = 0.476, *p* = 3.67 × 10^–3^), HLA-DRB1 (rho = 0.45, *p* = 6.35 × 10^–3^), and HLA-DOA1 (rho = 0.447,* p* = 6.79 × 10^–3^) (Fig. 4E–F). To enhance the robustness of the results, we also investigated the correlation between DPT and immunomodulators in the GSE26566 and GSE45001 datasets, confirming the positive correlation of DPT with the expression of most immunoinhibitors, immunostimulators, and MHCs, consistent with TCGA dataset analysis (Supplementary Fig. 4B–G). In summary, these results suggest that DPT plays a pivotal role in immune regulation in CHOL.

### DPT can significantly improve the response and survival of patients to anti-PD-1/PD-L1 immunotherapy

**Fig. 5:**
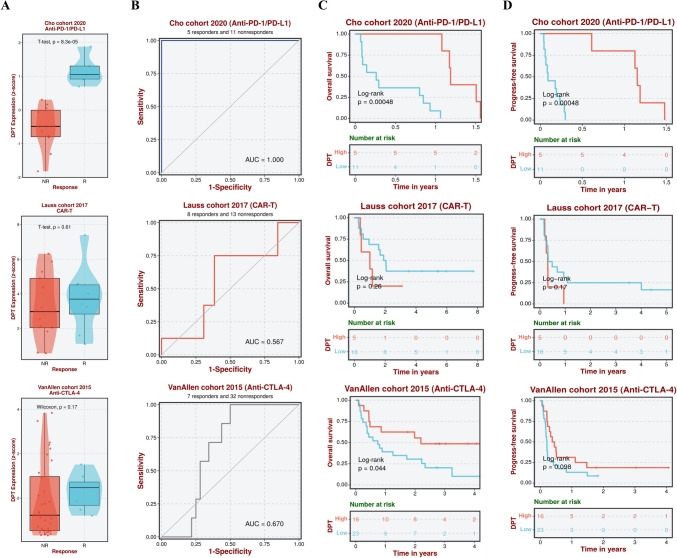
DPT may significantly improve patient response and survival to anti-PD-1/PD-L1 Immunotherapy. **A** The response effect in different therapeutic target study groups varied with the expression level of DPT. **B** The diagnostic specificity and sensitivity of different therapeutic targets for CHOL. **C–D** Impact of different immunotherapies on survival

We analyzed three research cohorts to investigate the association between DPT expression and sensitivity to specific immunotherapy, which included the Lauss cohort 2017, Cho cohort 2020, and the VanAllen cohort 2015. The results showed that high DPT expression was significantly associated with a positive response to anti-PD-1/PD-L1 therapy. Notably, the response sensitivity to CAR-T therapy in the Lauss cohort 2017 and anti-CTLA-4 therapy in the VanAllen cohort 2015 did not depend on DPT expression (Fig. 5A). The ROC curve further demonstrated the diagnostic sensitivity and efficacy of anti-PD-1/PD-L1 therapy in the Cho cohort 2020 (AUC = 1.00), while CAR-T therapy and anti-CTLA-4 therapy had lower diagnostic sensitivity and specificity with AUC values of 0.576 and 0.670, respectively (Fig. 5B). Kaplan–Meier survival curves for OS and PFS indicated that anti-PD-1/PD-L1 therapy in patients with high DPT expression significantly improved survival outcomes (OS:* p* = 4.8 × 10^–4^; PFS: *p* = 4.8 × 10^–4^). In contrast, CAR-T therapy (OS: *p* = 0.26; PFS: *p* = 0.17) and anti-CTLA-4 therapy (OS: *p* = 0.044; PFS: *p* = 0.098) had no significant impact (Fig. 5C–D). In conclusion, these findings suggest that anti-PD-1/PD-L1 therapy is more effective in patients with high DPT expression.

### DPT mutation landscape and correlations with clinical subgroups in CHOL

**Fig. 6:**
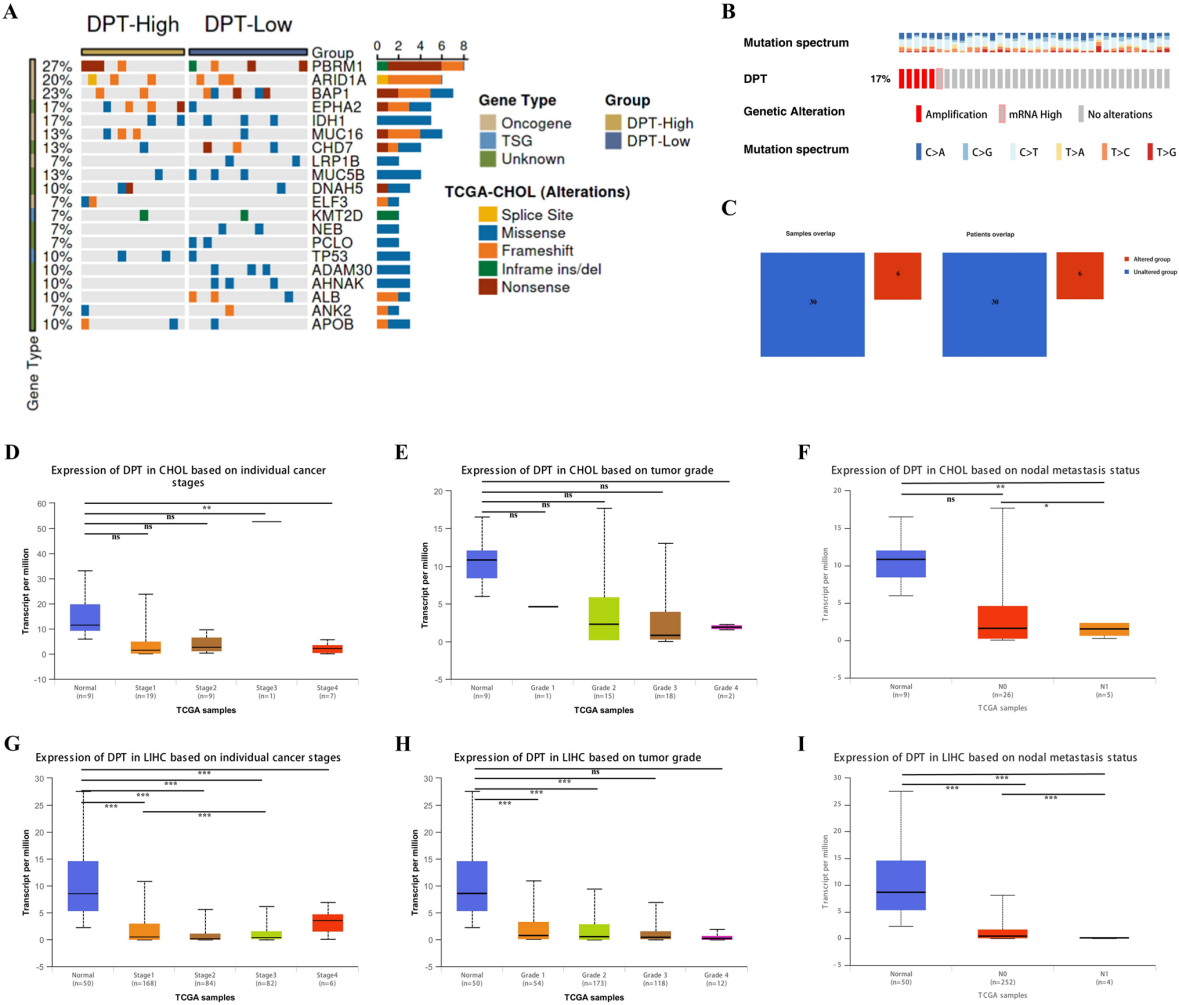
The landscape of DPT mutations and its links to clinical subgroups in CHOL. **A** camoip database showed the gene mutation frequency in the high- and low-DPT group. **B** Exhibition of mutation rate and major mutation types of DPT in CHOL using cBioPortal database. **D–F** Using UALAN website to show the relationship between DPT and tumor stage, grade, and lymph node grade of CHOL. **G–I** Using UALCAN website to show the relationship between DPT and tumor stage, grade, and lymph node grade of LIHC

CHOL is characterized by high heterogeneity and genetic variability (Wang and Du 2021; Yamaji-Hasegawa et al. 2022). We next investigated the relationship between DPT expression and somatic mutations using TCGA-CHOL data. As shown in Fig. 6A, the gene mutation rate in the high-DPT group was lower than in the low-DPT group, with missense mutations being the most prevalent. Additionally, DPT genomic alterations in CHOL were further analyzed using the cBioPortal database, which revealed that 17% of CHOL patients (6 altered and 30 unaltered) had DPT genomic alterations, mainly in the form of amplifications (Fig. 6B–C). This variation in DPT genomic status may contribute to differences in tumor grade and prognosis among CHOL patients. We also utilized the UALCAN database to explore the relationship between DPT expression and tumor grade, stage, and lymph node metastasis in CHOL and LIHC. The results indicated that DPT expression in CHOL and LIHC gradually decreased with increasing tumor grade, stage, and lymph node metastasis stage (Fig. 6D–F). However, this trend was not statistically significant in CHOL but showed a highly significant correlation with LIHC (Fig. 6G–I). Taken together, DPT expression is inversely correlated with somatic mutations as well as tumor grade, stage, and lymph node metastatic grade in both CHOL and LIHC.

### CCL19 as a potential target for DPT-Mediated Enhancement of Immune Cell Infiltration in CHOL

**Fig. 7:**
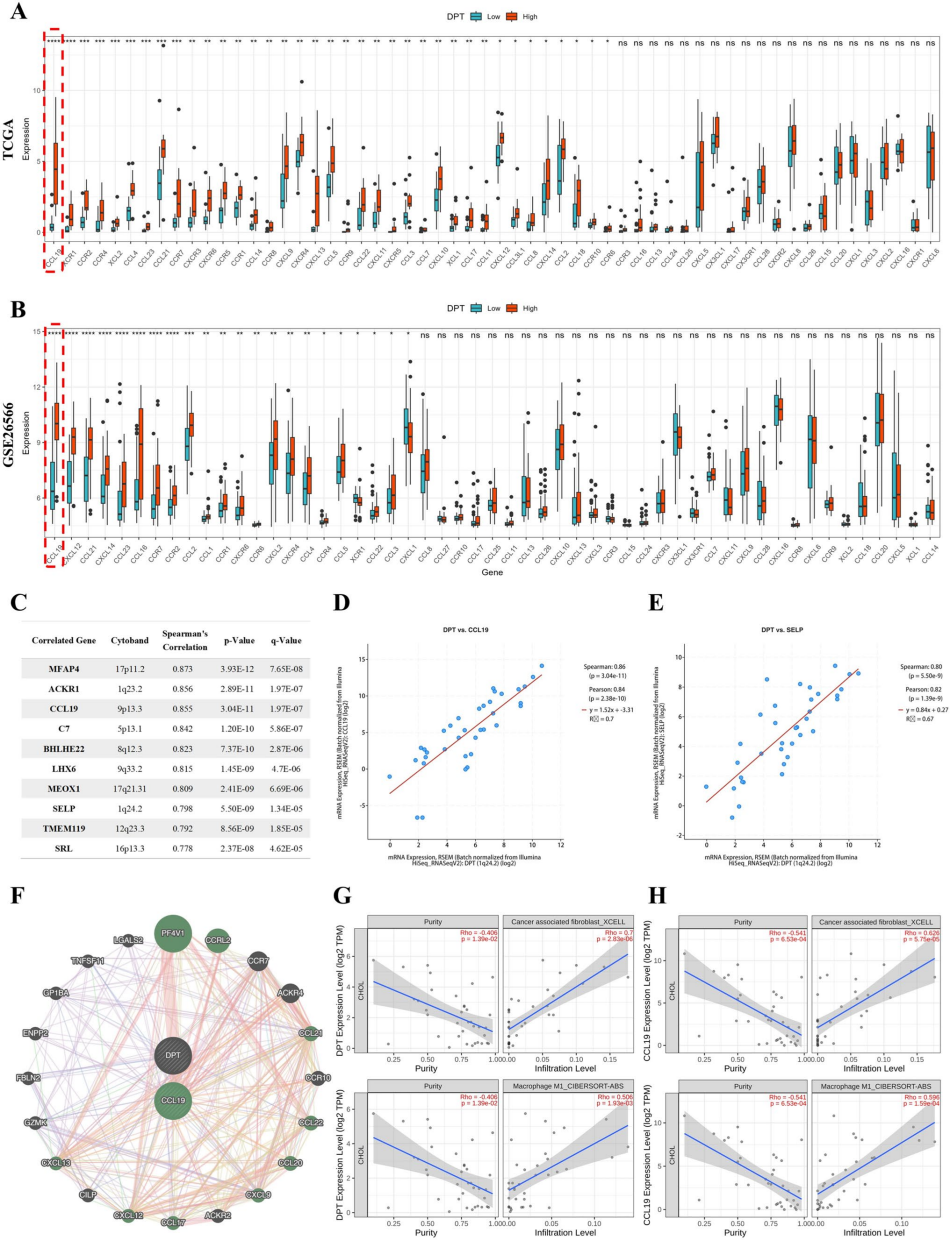
DPT may target CCL19 to promote immune cell infiltration in CHOL. **A–B** The intersection of DPT-related DEGs and chemokine genes in GSE26566 and TCGA datasets. **C** Exhibition of the top 10 genes most associated with DPT in CHOL using cBioPortal database. **D–F** Analysis of the correlation between DPT and chemokine CCL19, adhesion molecule SELP (Green represents chemokines). **G** Exploration of the relationship between DPT and macrophages, CAFs using Timer2.0 database. **H** Exploration of the relationship between CCL19 and macrophages, CAFs using Timer2.0 database

Given the correlations between DPT and immune infiltration and their downstream signal pathways, including the chemokine signaling pathway and cell adhesion molecules, we explored the relationships between DPT and chemokines and adhesion molecules. Our findings revealed significant changes in the expression levels of several chemokines and adhesion molecules between the high- and low-DPT groups in the TCGA and GSE26566 CHOL datasets (Fig. 7A–B and Supplementary Fig. 5A–B). Subsequently, we further investigated DPT-correlated genes using the cBioPortal website, revealing the top 10 DPT-correlated genes, along with their cytobands, Spearman's correlation coefficients, *p* values, and *q* values (Fig. 7C). Among the top 10 DPT-correlated genes, CCL19 was the only chemokine, and SELP was the only adhesion molecule. The relationships between DPT and CCL19 (Spearman = 0.86; *p* = 3.04 × 10–11) and SELP (Spearman = 0.80; *p* = 5.50 × 10–9) were visualized in scatter plots (Fig. 7D–E). Additionally, we confirmed the relationship between DPT and CCL19 and SELP using the GeneMANIA online database (Fig. 7F and Supplementary Fig. 5C). Previous studies have shown that CCL19 is primarily secreted by CAFs and macrophages, while SELP is mainly produced by endothelial cells (Cheng et al. 2018; Xuan et al. 2015; Yeini and Satchi-Fainaro 2022). Using Timer2.0, we found that DPT and CCL19 were both positively correlated with CAFs and macrophages (Fig. 7G–H). DPT and SELP were both positively correlated with endothelial cells (Supplementary Fig. 5D–E). However, since this study represents a preliminary exploration of the mechanisms underlying the low level of immune cell infiltration in the TME of CHOL. Meanwhile, the correlation of DPT with the chemokine signaling pathway is stronger than with the cell adhesion molecule pathway shown in Fi. 3C. Consequently, only the target (CCL19) of the chemokine signaling pathway was selected for further verification. Furthermore, we detected the expression level of CCL19 in CHOL using the GSE26566, GSE45001, and TCGA-CHOL datasets, and all findings indicated that CCL19 is lowly expressed in CHOL tissues compared with normal tissues (Supplementary Fig. 5F). At the same time, in CHOL patients, the high-CCL19 group showed better prognosis than the low-CCL19 group (Supplementary Fig. 5G). In summary, these results suggest that DPT may promote immune cell infiltration by increasing the production of downstream CCL19 and SELP.

### Single-cell analysis of CCL19 expression in various cells of CHOL TME

**Fig. 8:**
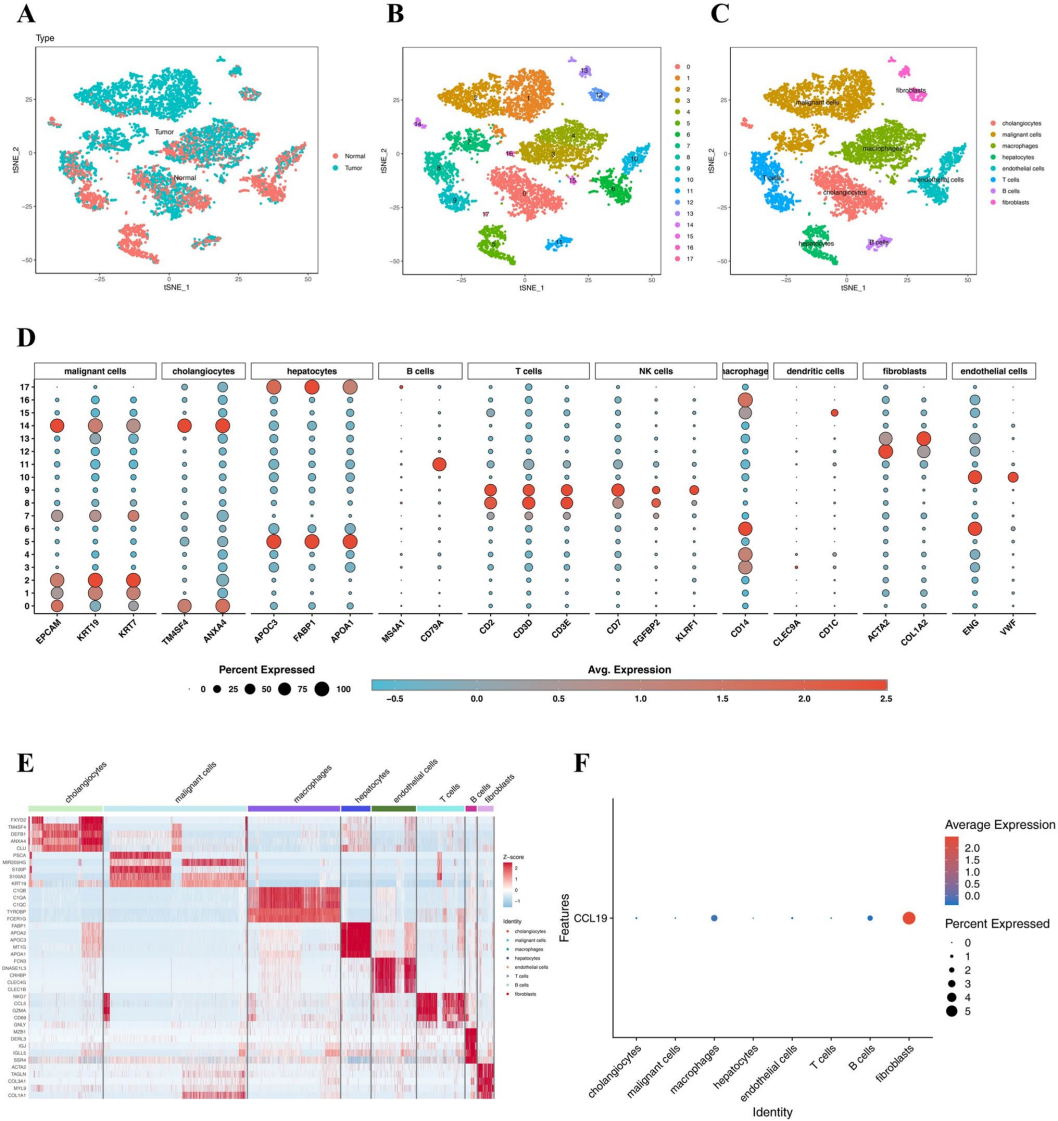
Single-cell analysis of CCL19 expression in different cells of CHOL TME. **A–C** The cells of CHOL were divided into 8 cell groups via principal component analysis and t-distributed stochastic neighbor embedding (tSNE). **D** The markers of these 8 cell groups and the clusters in which they are expressed. **E** Heatmap was used to mark the top 5 DEGs of each cell group. **F** The synthetic position of DPT in the CHOL TME was demonstrated using a bubble diagram

To determine the specific locations where CCL19, a potential downstream target of DPT, is synthesized in the CHOL TME, we analyzed the 10 × Genomics single-cell sequencing dataset GSE138709 obtained from the GEO database. Initially, all cells in the CHOL TME were categorized into two types: normal and tumor (Fig. 8A). Principal component analysis and t-distributed stochastic neighbor embedding (tSNE) were then applied for dimensionality reduction and clustering, resulting in the identification of 18 clusters (Fig. 8B). Based on classical markers for various cell types, these 18 clusters were annotated into eight cell groups: cholangiocytes (marked with TM4SF4, ANXA4), malignant cells (EPCAM, KRT19, and KRT7), macrophages (marked with CD14), hepatocytes (marked with APOC3, FABP1, and APOA1), endothelial cells (marked with ENG and VWF), T cells (marked with CD2, CD3D, and CD3E), B cells (marked with MS4A1 and CD79A), and fibroblasts (marked with ACTA2 and COL1A2) (Fig. 8C). We identified the DEGs for each cell group using the Wilcoxon rank-sum test and visualized the top 5 DEGs for each group in a heatmap (Fig. 8D–E). Finally, the expression level of the chemokine CCL19 was visualized in a bubble plot. CCL19 was predominantly expressed by CAFs and macrophages in the TME (Fig. 8F).

### DPT and CCL19 are lowly expressed in CHOL clinical tissues

**Fig. 9:**
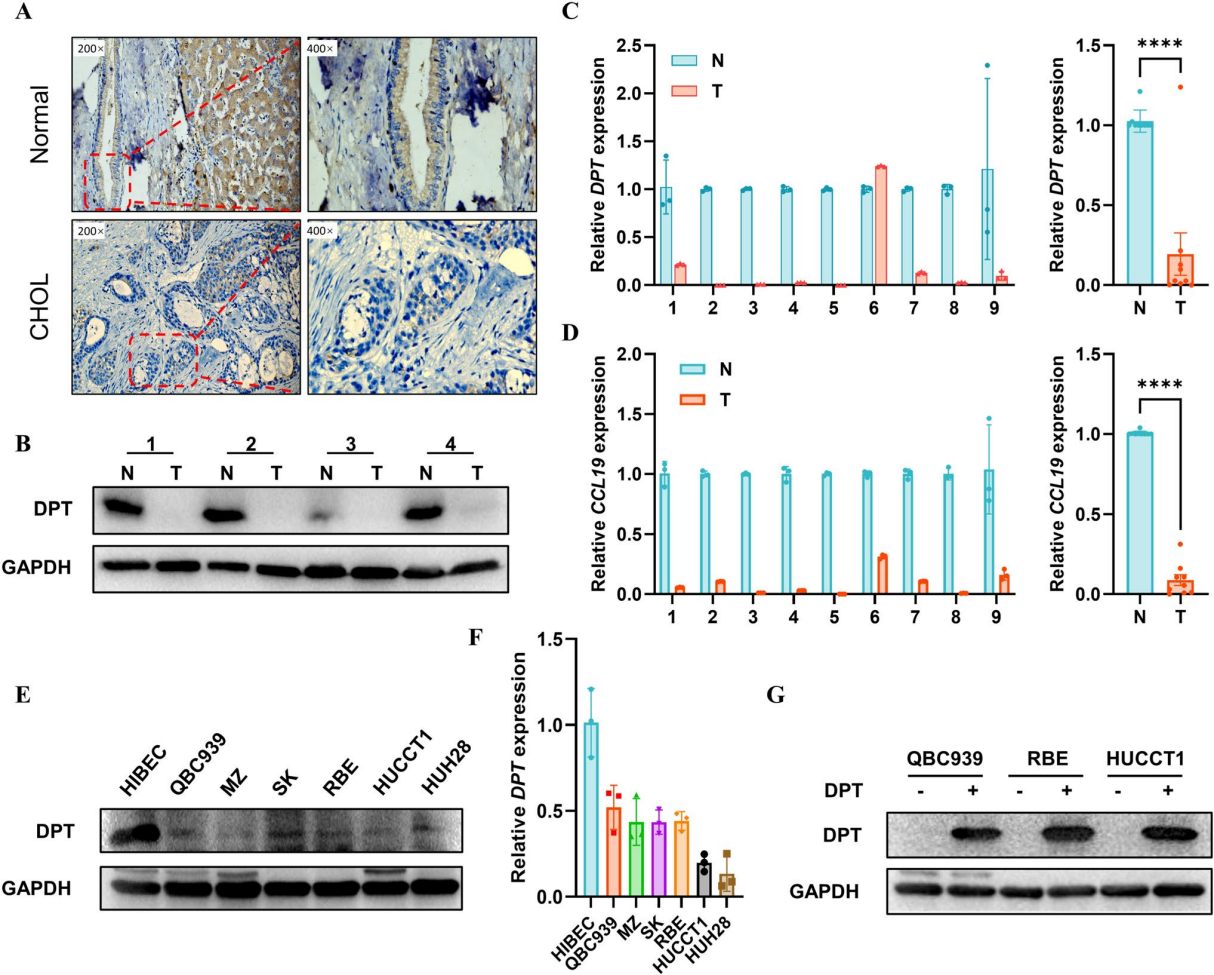
DPT and CCL19 is lowly expressed in CHOL cells and clinical tissues. **A** Detection of the DPT expression in normal bile duct epithelial and CHOL via immunohistochemistry. **B–C** Through qPCR and WB analysis, the expression of DPT mRNA and protein in normal bile duct epithelial and CHOL specimens was detected. **D** qPCR was performed to assess the expression of CCL19 mRNA in CHOL tissues. **E–F** mRNA and protein level of DPT in normal biliary cells (HIBEC) and various CHOL cell lines (QBC939, MZ, SK, RBE, HUCCT1, and HUH28). **G** Construction of CHOL cell lines overexpressing DPT by lentivirus transfection

To confirm the robustness of the results obtained from database analysis, we collected paired CHOL specimens for further investigation. Initially, we conducted IHC analysis to assess the expression of DPT in CHOL tissues, revealing a significant decrease in DPT protein expression compared to normal bile duct epithelial tissues (Fig. 9A). Furthermore, we performed qPCR and WB to measure the levels of DPT mRNA and protein, and the results exhibited a consistent trend with the IHC analysis (Fig. 9B–C). Likewise, we observed a higher expression of CCL19 in normal bile duct epithelial tissues compared to CHOL tissues (Fig. 9D). At the cellular level, we cultured several cell lines, including normal bile duct cells and CHOL cells, for the detection of DPT gene and protein. Similarly, DPT gene and protein expression were significantly higher in normal bile duct cells compared to CHOL cell lines (Fig. 9E–F). Finally, we selected various CHOL cell lines, such as QBC939, HUCCT1, and RBE, to create DPT-overexpressed cell models for further analysis (Fig. 9G). In conclusion, DPT was significantly downregulated in CHOL tissues and cell lines, with low expression of CCL19 in these tissues. These findings provide the basis for further exploring the relationship between DPT and CCL19.

### CHOL cells derived DPT promotes the secretion of CCL19 in macrophages to trigger immune infiltration

**Fig. 10:**
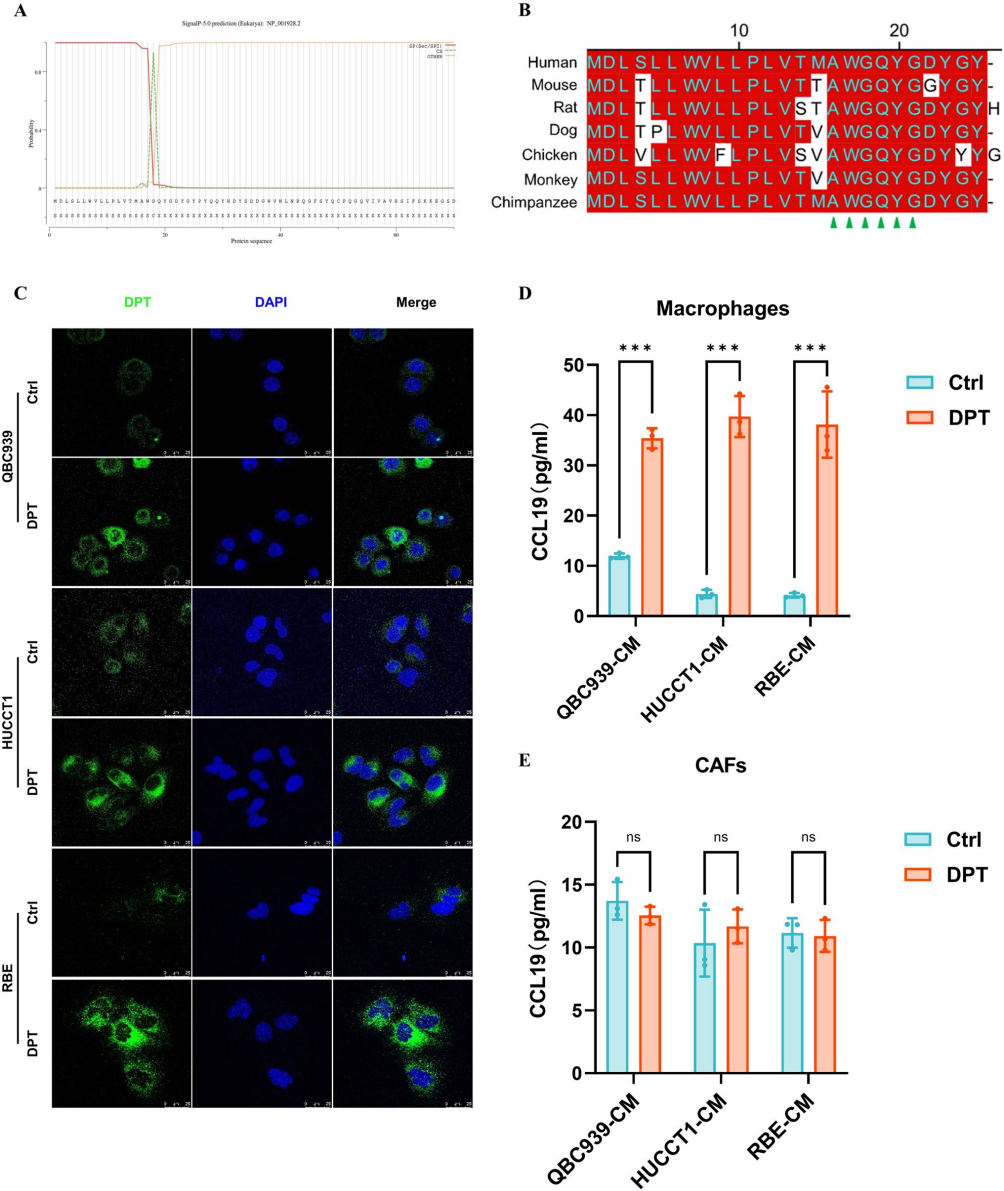
DPT overexpression in CHOL cells stimulates macrophages to produce CCL19. **A** The signal peptide structure in front of the DPT protein sequence was found using the SignalP-5.0 website. **B** The sequence near the cleavage site of DPT signal peptide was consistent in various species using MegAlign analysis (human, mouse, rat, dog, chicken, monkey, and chimpanzee). **C** Confocal microscopy exhibited the localization of DPT in various CHOL cell lines. **D–E** Through ELISA assay, the effect of exocrine DPT on the secretion of CCL19 by macrophages but CAFs was detected

To further validate that DPT can elevate the expression of the chemokine CCL19, we analyzed the DPT protein sequence using the SignalP-5.0 website. Our analysis revealed the presence of a signal peptide at the beginning of the DPT protein, with the cleavage site between positions 18 and 19: AWG-QY (probability: 0.9329). This suggests that DPT may be secreted into the TME as an exocrine protein (Fig. 10A). Additionally, we compared the DPT signal peptide sequences in various species, including humans, mouse, rats, dogs, chickens, monkeys, and chimpanzees, and found that the sequences near the cleavage site were highly conserved (Fig. 10B). Furthermore, immunofluorescence analysis of different CHOL wild-type and DPT-overexpressing cell lines showed that DPT is primarily localized in the cytoplasm rather than the nucleus (Fig. 10C). Based on this previous research and the evidence from immunofluorescence and bioinformatics analysis, we speculate that DPT functions as an exocrine protein in the CHOL TME (Okamoto and Fujiwara 2006; Takeuchi et al. 2006). Next, we investigated whether DPT secreted by CHOL cells can affect two common cell types in the TME, namely, CAFs and macrophages, to enhance CCL19 expression. ELISA and co-culture experiments indicated that macrophages, co-cultured with conditioned media from DPT-overexpressing CHOL cells, exhibited significantly higher CCL19 secretion compared to the control group (Fig. 10D). However, no significant changes were observed in the CAFs co-culture system (Fig. 10E). In summary, these findings demonstrate that CHOL cells overexpressing DPT can promote the secretion of the chemokine CCL19 in macrophages, resulting in increased immune cell infiltration in the TME, explaining the beneficial effects of DPT in improving CHOL prognosis.

## Discussion

CHOL is a highly interstitial and heterogeneous epithelial tumor, and the TME is typically immunosuppressed, characterized by the absence of antigen-presenting cells, reduced immune cell infiltration, and the upregulation of immunosuppressive molecules like PD-L1. These features contribute to the immune escape of tumor cells, potentially causing resistance to drugs such as gemcitabine, cisplatin, and anti-PD-1/PD-L1 antibodies. Therefore, more research is warranted to understand the relationship between CHOL and the TME.

Herein, we identified 9 DEGs as significant for CHOL patients' prognosis: BUB1B, TROAP, CST1, GPAM, MT1F, PAH, CFHR3, DPT, and FCN1. After a comprehensive review of various studies, DPT emerged as the key target to influence tumor development and CHOL patients' prognosis.

DPT, an extracellular matrix (ECM) protein rich in tyrosine, is located on chromosome 1q32.1 and consists of 183 amino acid residues (Huang et al. 2021; Kim et al. 2019). It primarily contributes to ECM formation and regulation, influencing various biological processes like cell adhesion, migration, and invasion (Guo et al. 2019; Kramer et al. 2021; Seetaraman Amritha et al. 2020; Unamuno et al. 2020). Previous studies have shown that DPT can strongly inhibit various tumor-related signaling pathways, including Wnt/β-catenin, TGF-β, Hippo/YAP, and the ERK/MAPK pathway. Moreover, the expression of DPT has been linked to the development and prognosis of cancers (Patel et al. 2016; Xi et al. 2018). For instance, DPT can modulate the connection and adhesion of liver cancer and oral cancer cells to the ECM, thereby affecting the invasion and migration capacity (Fu et al. 2014; Yamatoji et al. 2012); It can also regulate the MEK-ERK-MYC signaling pathway, inhibiting the expression of downstream proteins such as CDK4, CDK6, and p21, which, in turn, suppresses the proliferation of thyroid cancer cells. Despite the established role of DPT in preventing the growth of various cancers, its relationship with the TME and immune cells remains relatively unexplored.

In recent decades, researchers have increasingly recognized the significance of the TME and immune infiltration, as both have a considerable impact on tumor development and patient prognosis (Dieci et al. 2021; Liu et al. 2022; Wang et al. 2020). In our study, we established a positive correlation between the expression level of DPT and immune infiltration, including immune killer cells such as CD4^+^ T cells, CD8^+^ T cells, B cells, and NK cells, and a negative correlation with myeloid-derived suppressor cells (MDSC) and M2 macrophages. Notably, CAFs exhibited the strongest correlation. These findings suggest that DPT may enhance the prognosis of CHOL patients by regulating the infiltration of various immune cell types. In the TME, immune modulators such as immunoinhibitors, immunostimulators, and MHC molecules play pivotal roles in tumor development and progression. Immune checkpoint molecules, including PD-1, PD-L1, CTLA4, and TIM-3, can induce immune cell exhaustion, resulting in an unfavorable prognosis (Ma et al. 2019; Viramontes et al. 2022). In contrast, immunostimulators like CD28 enhance T-cell activation and proliferation, promoting anti-tumor immune responses (Velasquez et al. 2017). It is widely thought that various HLA family molecules can present tumor antigens to T cells, facilitating tumor clearance by immune cells. This study indicates that when DPT is highly expressed, immune modulators, particularly immunostimulators and MHC molecules, are also highly expressed, contributing to the improved prognosis of CHOL patients with high DPT expression. Recently, immunotherapy has gained attention as a promising approach to cancer treatment. However, a significant portion of patients do not respond to immunotherapy (Riley et al. 2019; Topalian et al. 2020; Zhang and Zhang 2020). In our research, DPT emerged as a critical gene that enhances the response to PD-1/PD-L1 immunotherapy. Additionally, DPT can synergize with PD-1/PD-L1 immunotherapy to improve patient prognosis, in contrast to anti-CTLA4 and CART immunotherapy. Although DPT is an immune-related gene that effectively increases immune infiltration and modulator expression, the underlying mechanism remains unknown. Both chemokines and adhesion molecules are essential for facilitating immune cell access to the TME (Nagarsheth et al. 2017; Somasiri et al. 2004). Chemokines are a kind of small cytokines or signal proteins released by cells that can trigger directed chemotaxis of immune cells. Adhesion proteins can help immune cells migrate and localize to specific tissues by mediating cell–cell and cell–matrix interactions, modulating signal transduction, and influencing immune cell subset differentiation. All these processes are crucial for the maintenance of immune function and immune homeostasis. Therefore, we hypothesized whether there is a connection between DPT and chemokines, adhesins, which would mediate the effects mentioned above.

Through database analysis and basic experiments, we found that DPT and CCL19 were underexpressed in CHOL tissues and cell lines. CCL19 was selected as a candidate target for understanding the effects of DPT on immune infiltration. It is now understood that CCL19, a member of the chemokine family, participates in immune system regulation and inflammatory processes. Numerous studies have demonstrated that CCL19 is mainly secreted by macrophages and CAFs (Fujimura and Aiba 2020; Hogstrom et al. 2023). Our study suggests that exocrine DPT stimulates macrophages to release CCL19 but has no impact on CAFs. This could be due to the absence of a DPT receptor in CAFs, resulting in no significant changes in the CCL19-related signaling pathway. Alternatively, this study sought to demonstrate that DPT enhances immune infiltration via CCL19 and improves patient prognosis, while CAFs primarily promote cancer in the TME. Recent research has shown that local injection of CCL19-producing mesenchymal stem cells into murine TME can increase immune cell infiltration, enhancing the efficacy of anti-PD-L1 antibodies (Iida et al. 2020). These findings align with our research objectives. Although we explored the connection between DPT and CCL19 in vitro through ELISA assays and co-culture systems, further validation in *vivo* is needed. Our ultimate goal is to identify a novel immune-related biomarker and provide initial insights into its potential mechanism for regulating immune infiltration and immunotherapy.

## Conclusions

This study underscores the significant role of DPT in modulating immune infiltration within the CHOL TME, making it a potential marker for predicting CHOL patient prognosis and guiding clinical immunotherapy.

## Supplementary Information

Below is the link to the electronic supplementary material.

Supplementary file1 (ZIP 153266 KB)

Supplementary file2 (ZIP 4862 KB)

Supplementary file3 (PDF 1117 KB)

## Data Availability

Publicly available datasets were analyzed in this study. UCSC Xena: https://xena.ucsc.edu/ (accessed on 15 November 2022). GEO database: https://www.ncbi.nlm.nih.gov/geo/ (accessed on 5 December 2022).
