# Supplementary figures and images for: Downregulation of dermatopontin in cholangiocarcinoma cells suppresses CCL19 secretion of macrophages and immune infiltration
Source: J Cancer Res Clin Oncol. 2024 Feb 1;150(2):66. doi: 10.1007/s00432-023-05532-1 (PMC10834663; doi:10.1007/s00432-023-05532-1)

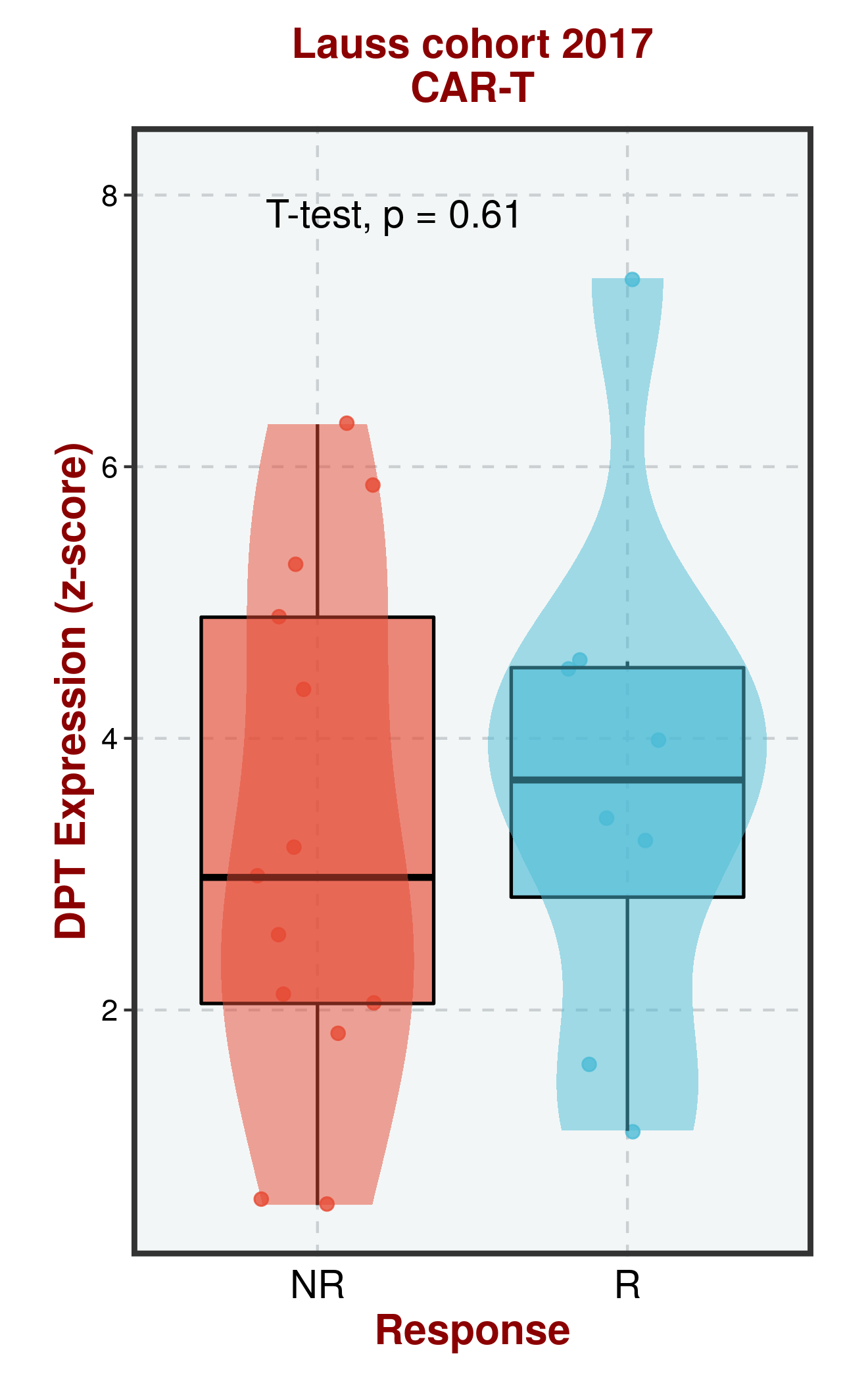

Supplement: Supplementary file 1 — Supplementary file1 (ZIP 153266 KB) [file 432_2023_5532_MOESM1_ESM.zip › Websites/BEST/Differential expression analysis/BEST_SingleGene_Immunotherapy_Expression_DPT_hgbkrK6Ffi/Plot_GSE100797.png]

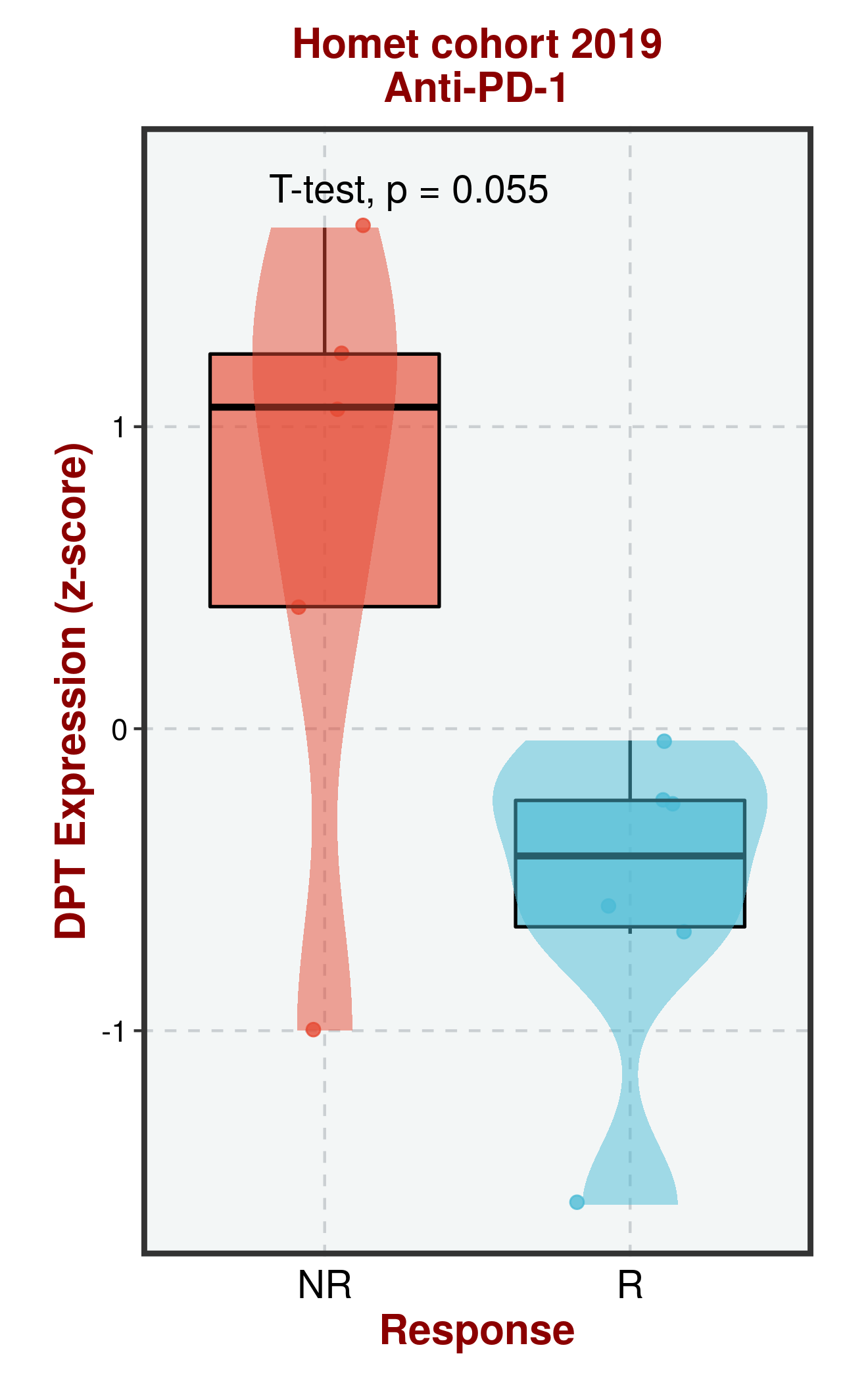

Supplement: Supplementary file 1 — Supplementary file1 (ZIP 153266 KB) [file 432_2023_5532_MOESM1_ESM.zip › Websites/BEST/Differential expression analysis/BEST_SingleGene_Immunotherapy_Expression_DPT_hgbkrK6Ffi/Plot_GSE111636.png]

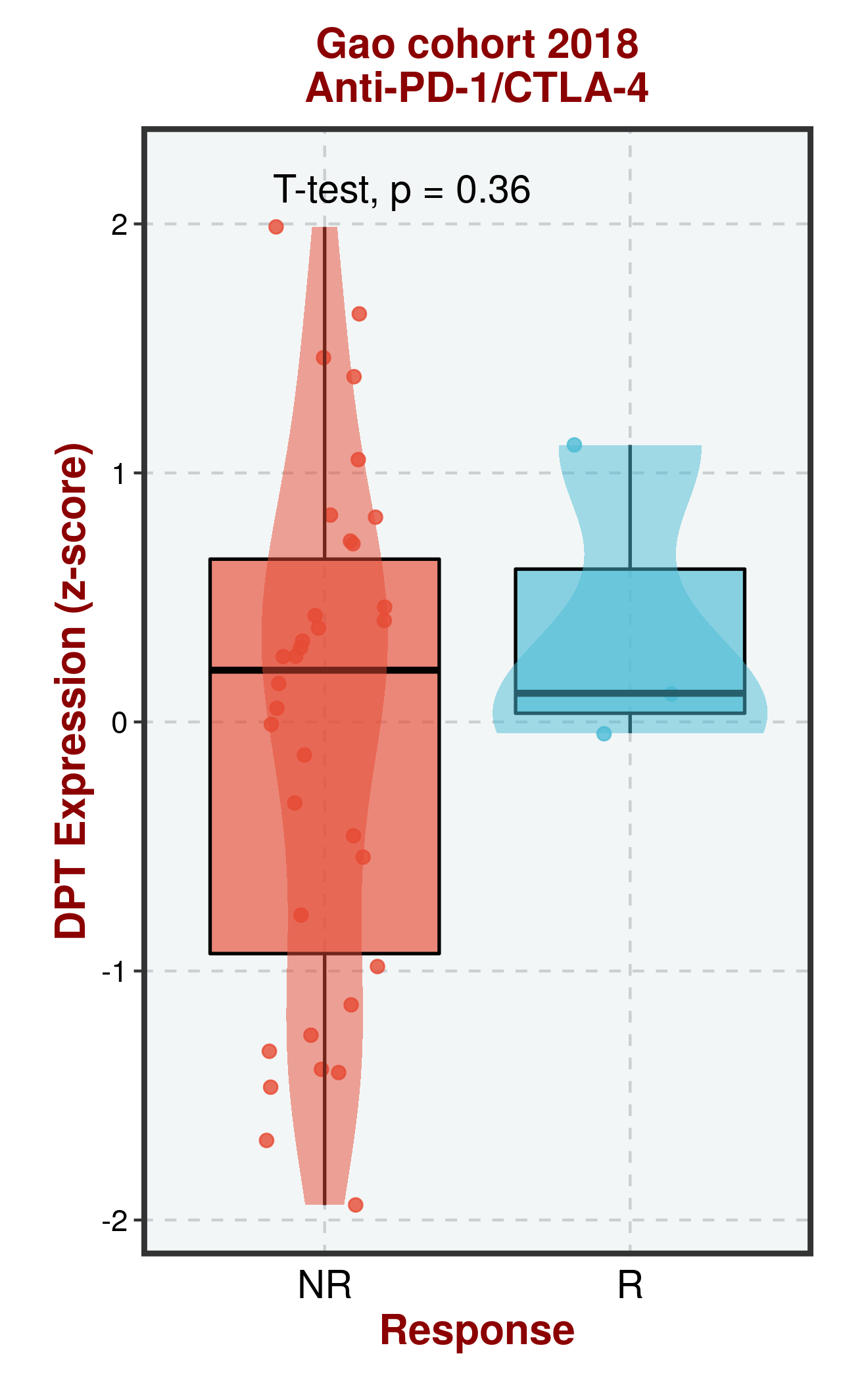

Supplement: Supplementary file 1 — Supplementary file1 (ZIP 153266 KB) [file 432_2023_5532_MOESM1_ESM.zip › Websites/BEST/Differential expression analysis/BEST_SingleGene_Immunotherapy_Expression_DPT_hgbkrK6Ffi/Plot_GSE115821.png]

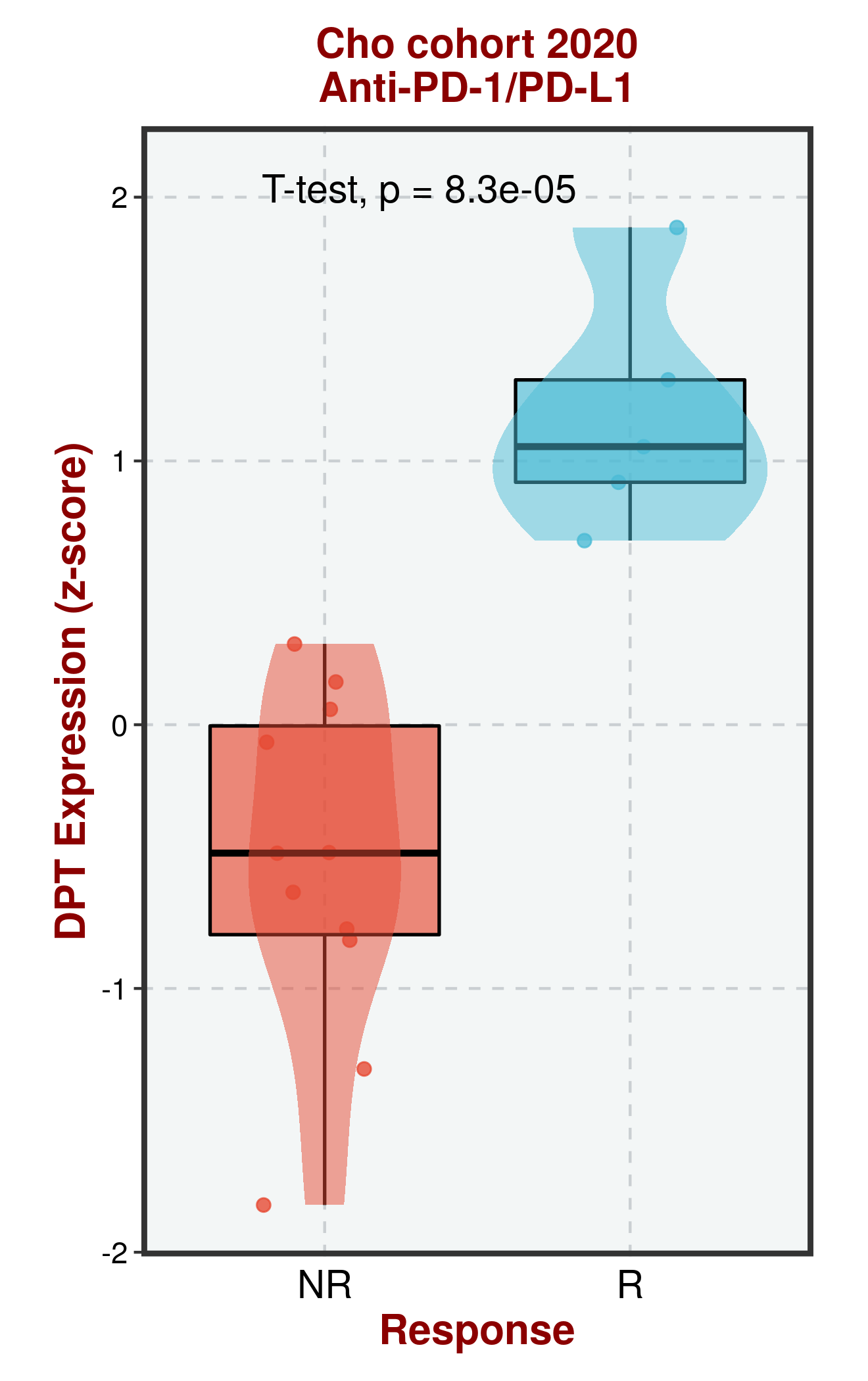

Supplement: Supplementary file 1 — Supplementary file1 (ZIP 153266 KB) [file 432_2023_5532_MOESM1_ESM.zip › Websites/BEST/Differential expression analysis/BEST_SingleGene_Immunotherapy_Expression_DPT_hgbkrK6Ffi/Plot_GSE126044.png]

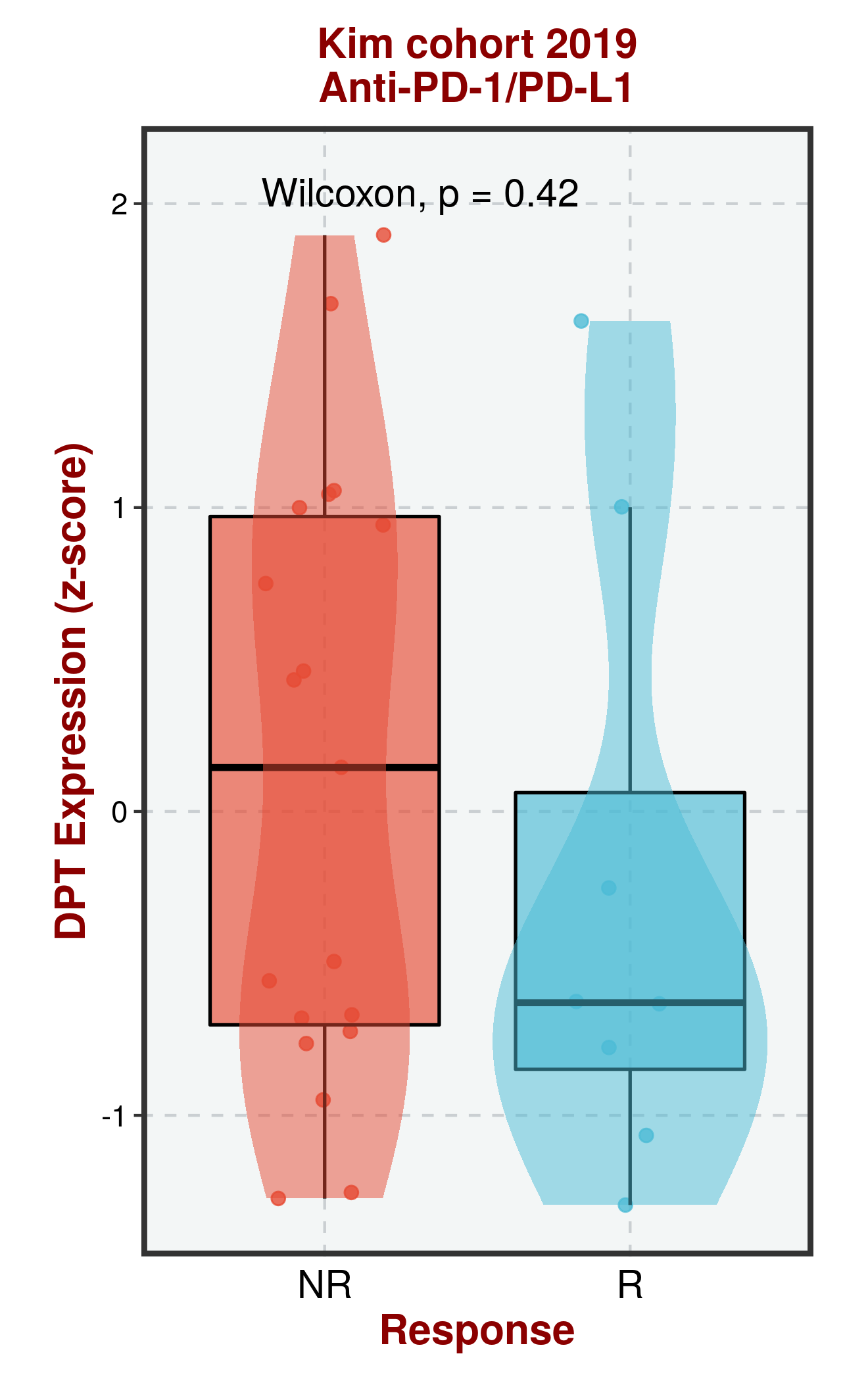

Supplement: Supplementary file 1 — Supplementary file1 (ZIP 153266 KB) [file 432_2023_5532_MOESM1_ESM.zip › Websites/BEST/Differential expression analysis/BEST_SingleGene_Immunotherapy_Expression_DPT_hgbkrK6Ffi/Plot_GSE135222.png]

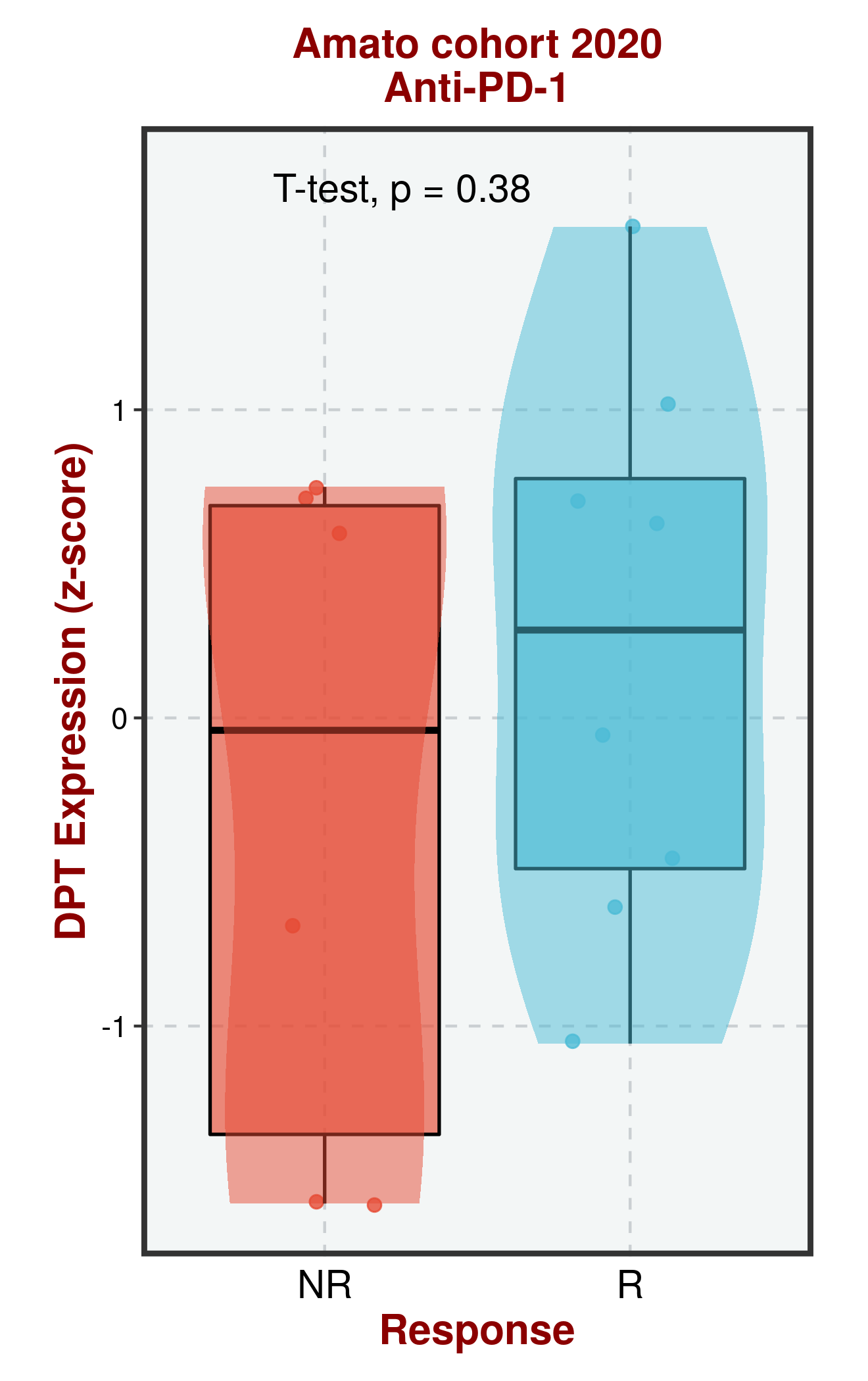

Supplement: Supplementary file 1 — Supplementary file1 (ZIP 153266 KB) [file 432_2023_5532_MOESM1_ESM.zip › Websites/BEST/Differential expression analysis/BEST_SingleGene_Immunotherapy_Expression_DPT_hgbkrK6Ffi/Plot_GSE145996.png]

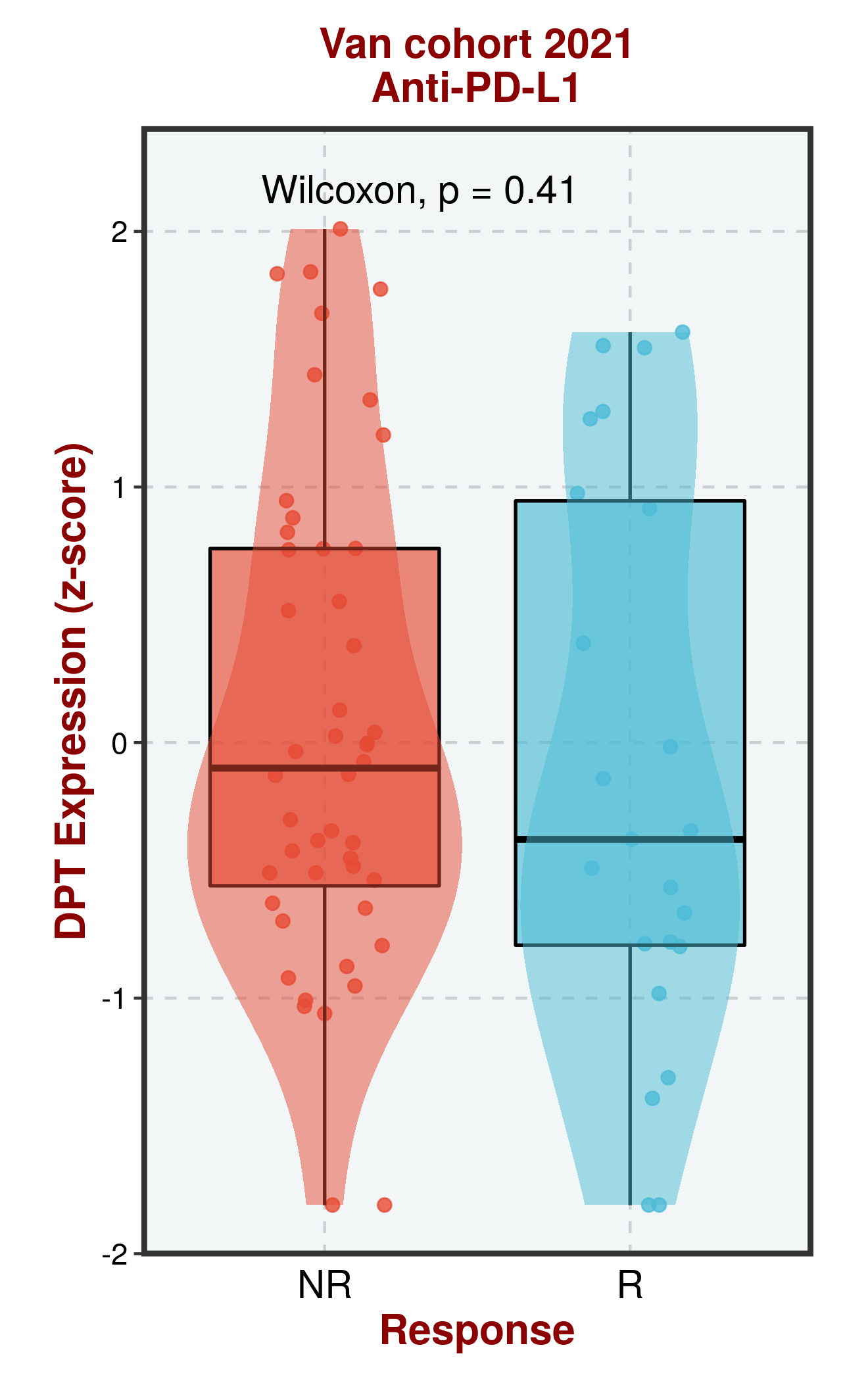

Supplement: Supplementary file 1 — Supplementary file1 (ZIP 153266 KB) [file 432_2023_5532_MOESM1_ESM.zip › Websites/BEST/Differential expression analysis/BEST_SingleGene_Immunotherapy_Expression_DPT_hgbkrK6Ffi/Plot_GSE165252.png]

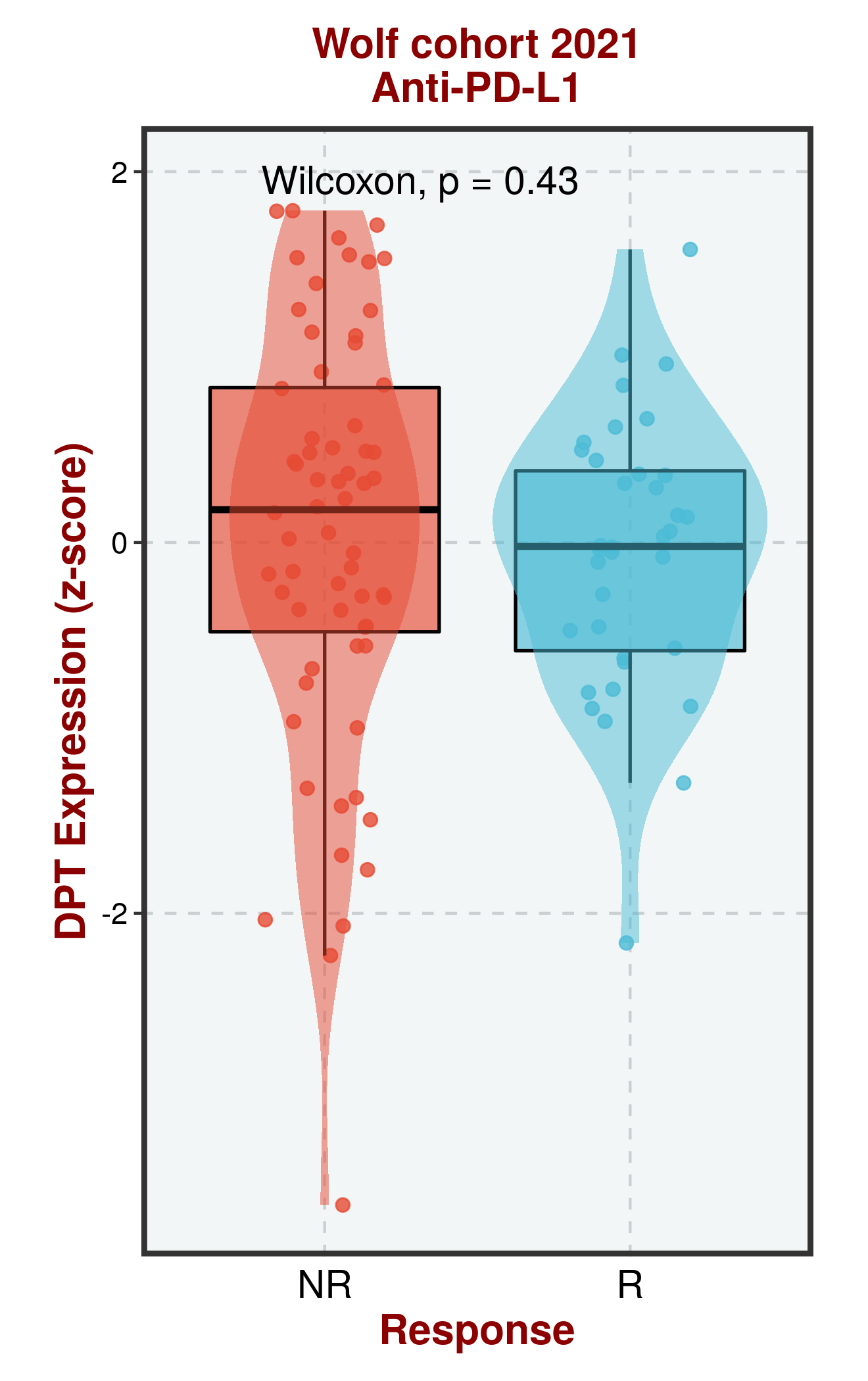

Supplement: Supplementary file 1 — Supplementary file1 (ZIP 153266 KB) [file 432_2023_5532_MOESM1_ESM.zip › Websites/BEST/Differential expression analysis/BEST_SingleGene_Immunotherapy_Expression_DPT_hgbkrK6Ffi/Plot_GSE173839.png]

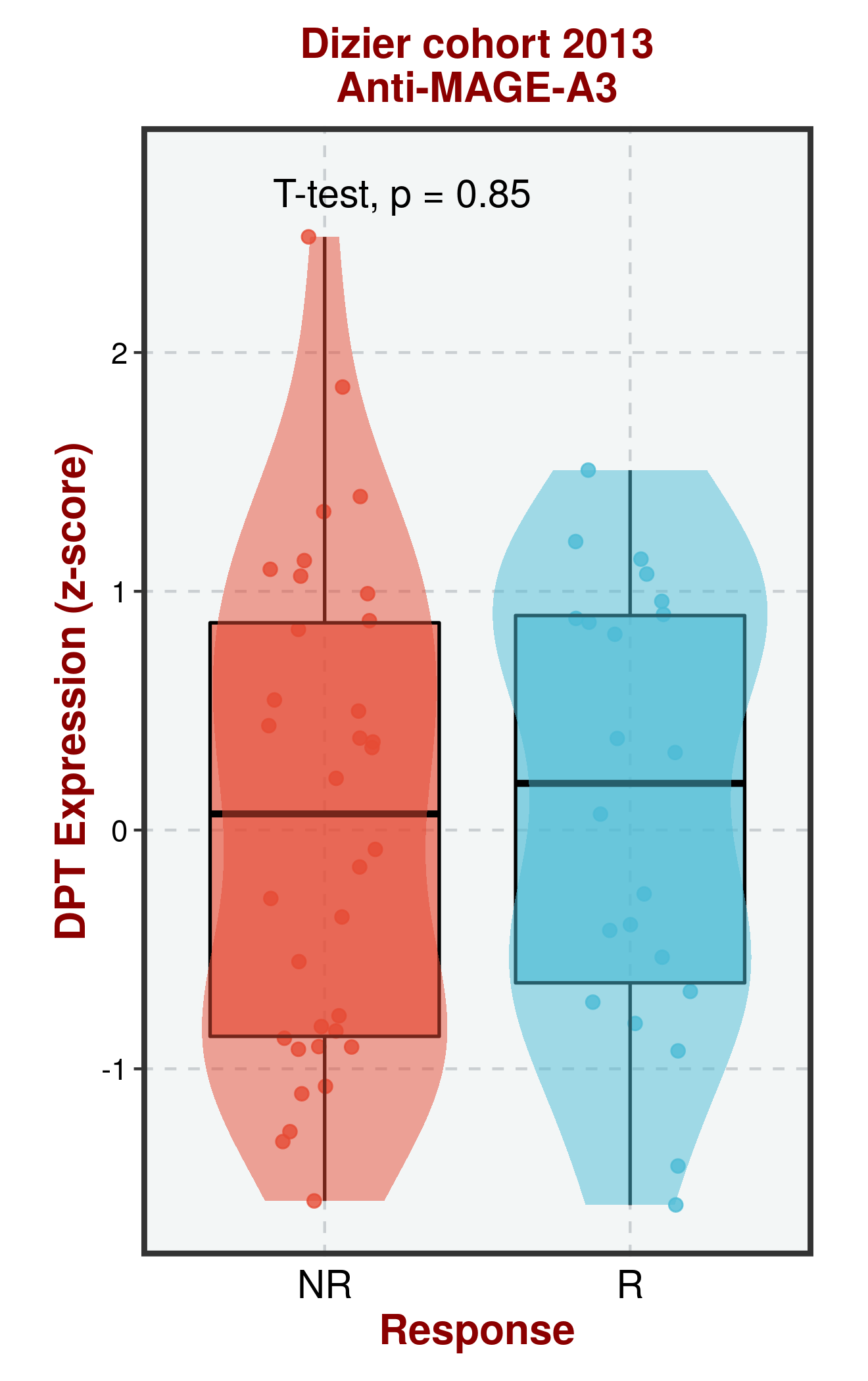

Supplement: Supplementary file 1 — Supplementary file1 (ZIP 153266 KB) [file 432_2023_5532_MOESM1_ESM.zip › Websites/BEST/Differential expression analysis/BEST_SingleGene_Immunotherapy_Expression_DPT_hgbkrK6Ffi/Plot_GSE35640.png]

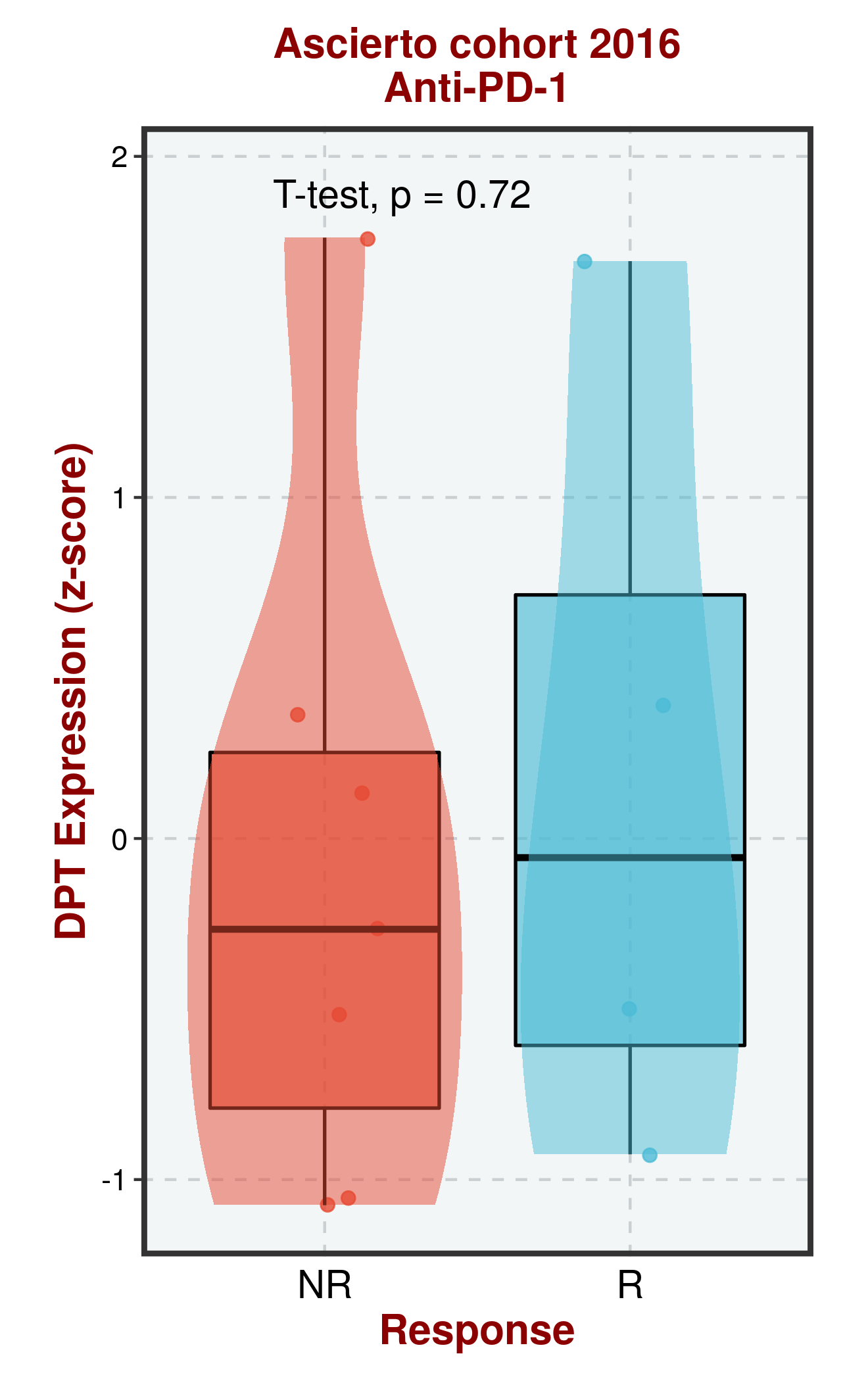

Supplement: Supplementary file 1 — Supplementary file1 (ZIP 153266 KB) [file 432_2023_5532_MOESM1_ESM.zip › Websites/BEST/Differential expression analysis/BEST_SingleGene_Immunotherapy_Expression_DPT_hgbkrK6Ffi/Plot_GSE67501.png]

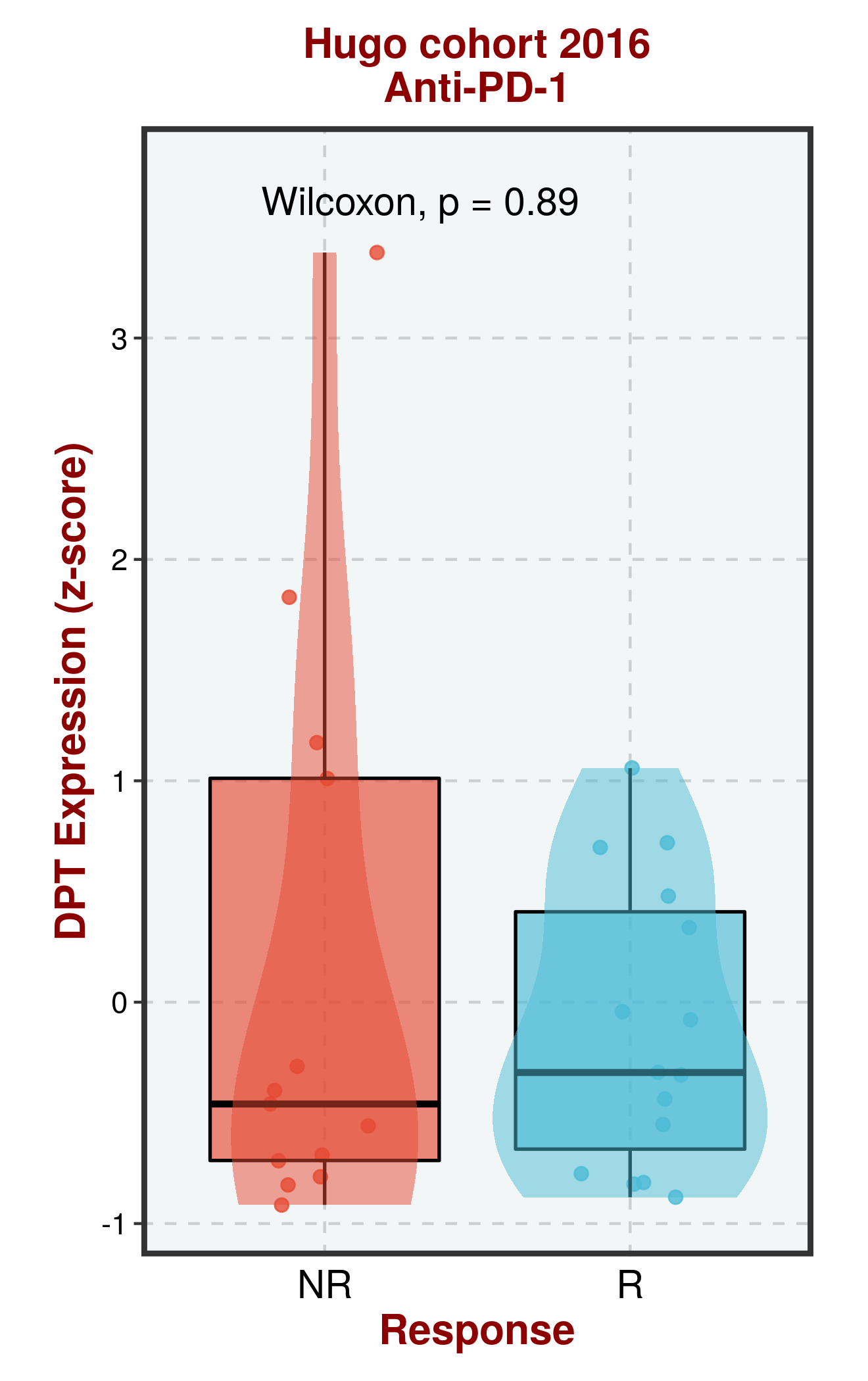

Supplement: Supplementary file 1 — Supplementary file1 (ZIP 153266 KB) [file 432_2023_5532_MOESM1_ESM.zip › Websites/BEST/Differential expression analysis/BEST_SingleGene_Immunotherapy_Expression_DPT_hgbkrK6Ffi/Plot_GSE78220.png]

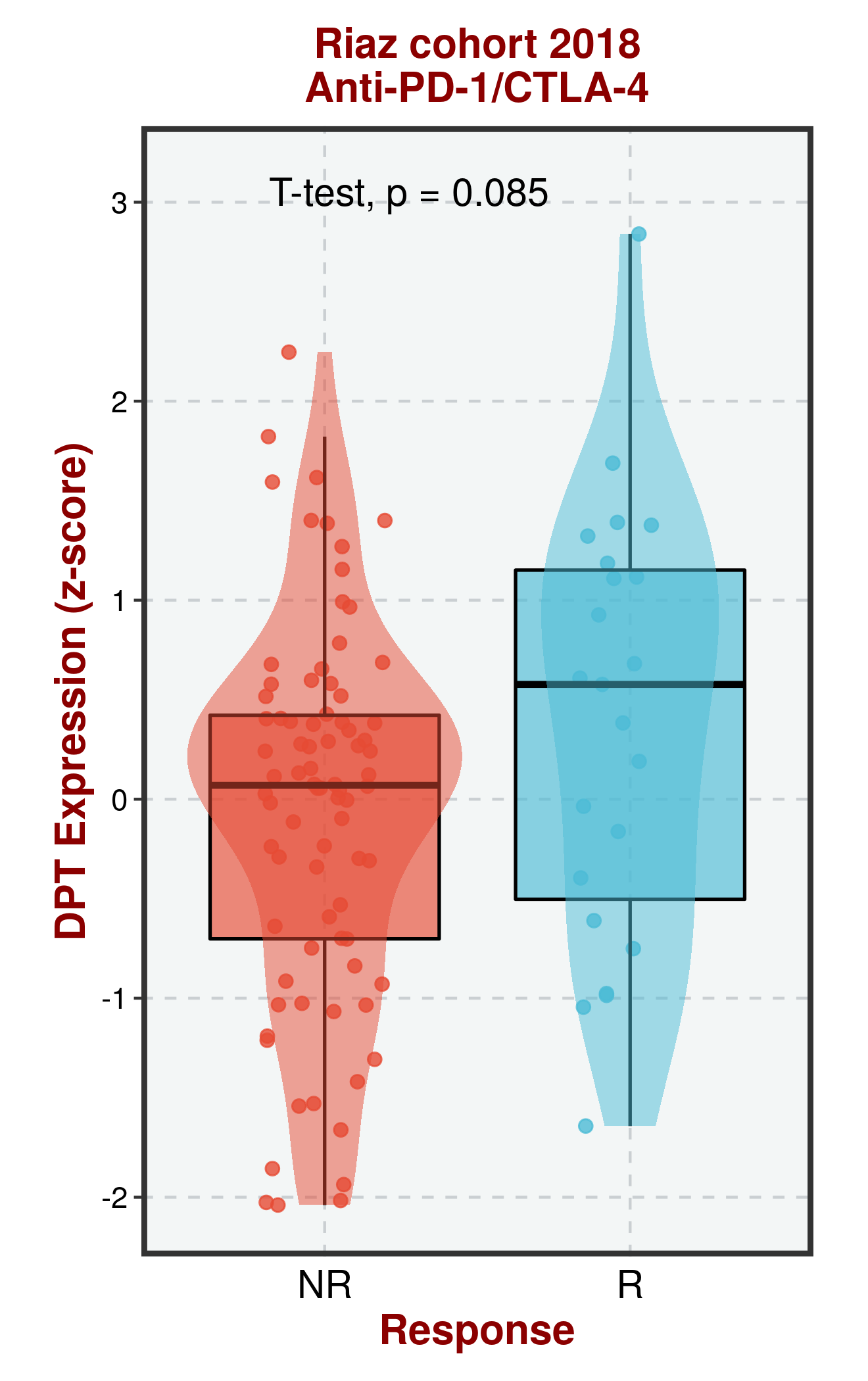

Supplement: Supplementary file 1 — Supplementary file1 (ZIP 153266 KB) [file 432_2023_5532_MOESM1_ESM.zip › Websites/BEST/Differential expression analysis/BEST_SingleGene_Immunotherapy_Expression_DPT_hgbkrK6Ffi/Plot_GSE91061.png]

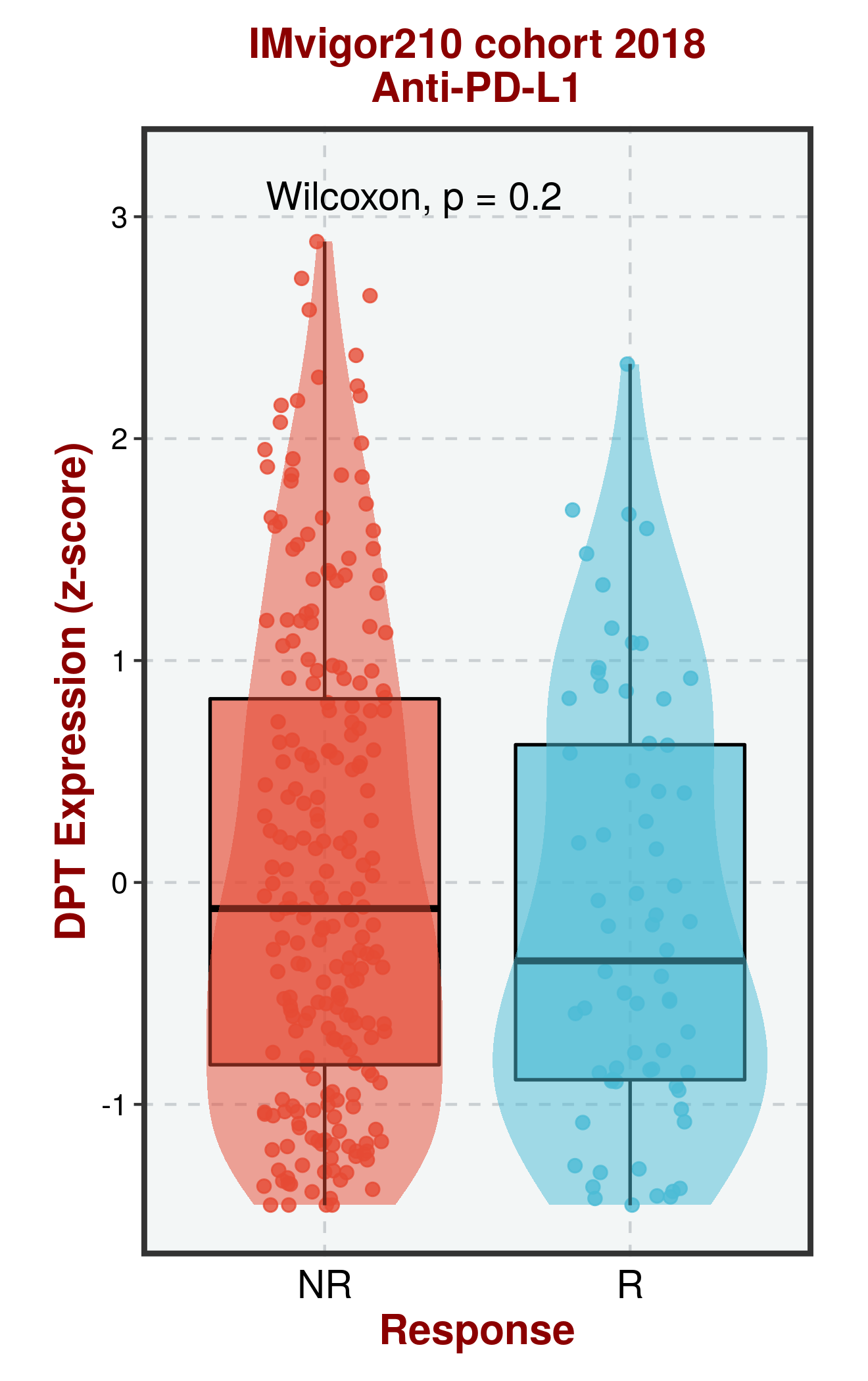

Supplement: Supplementary file 1 — Supplementary file1 (ZIP 153266 KB) [file 432_2023_5532_MOESM1_ESM.zip › Websites/BEST/Differential expression analysis/BEST_SingleGene_Immunotherapy_Expression_DPT_hgbkrK6Ffi/Plot_IMvigor210.png]

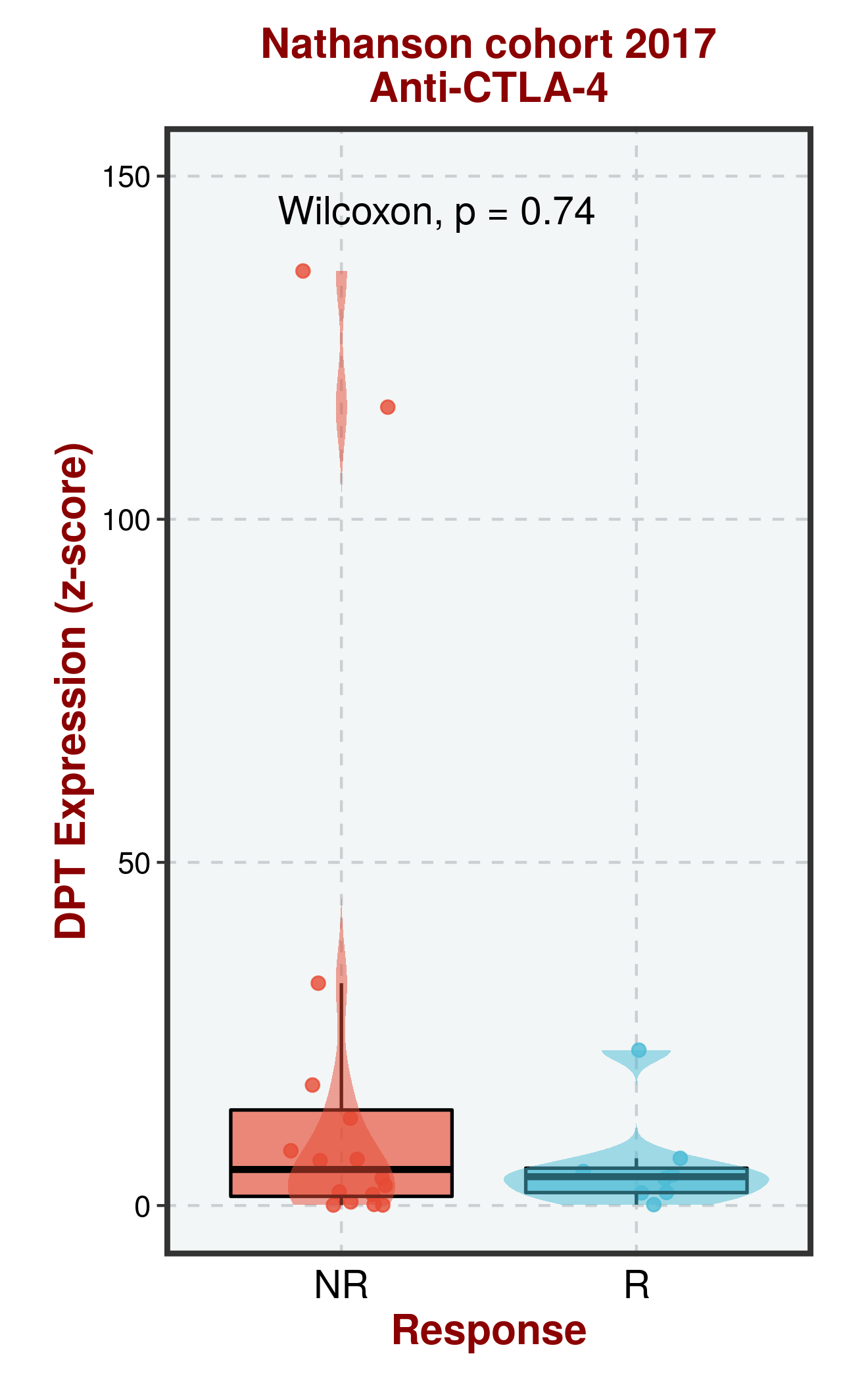

Supplement: Supplementary file 1 — Supplementary file1 (ZIP 153266 KB) [file 432_2023_5532_MOESM1_ESM.zip › Websites/BEST/Differential expression analysis/BEST_SingleGene_Immunotherapy_Expression_DPT_hgbkrK6Ffi/Plot_Nathanson.png]

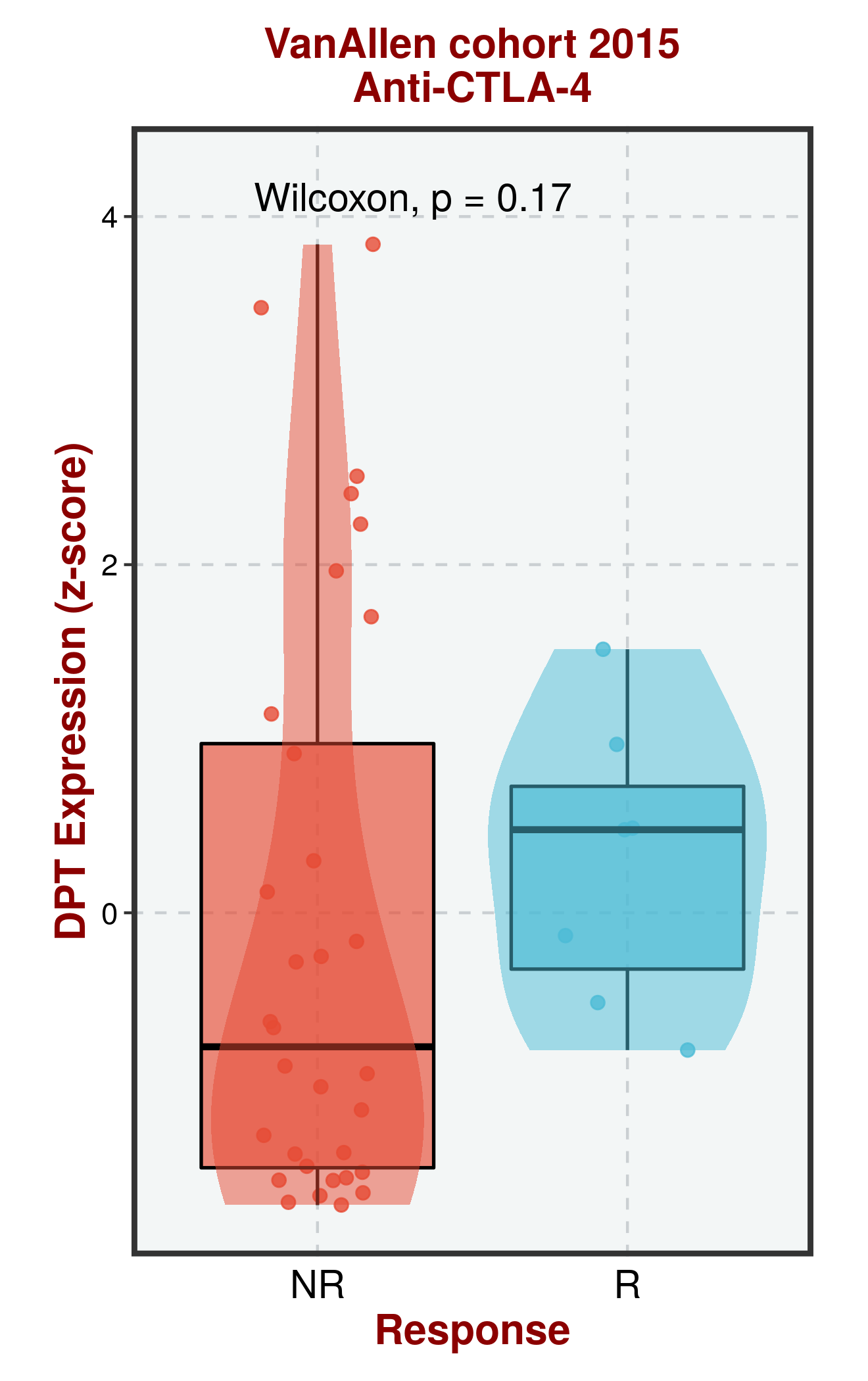

Supplement: Supplementary file 1 — Supplementary file1 (ZIP 153266 KB) [file 432_2023_5532_MOESM1_ESM.zip › Websites/BEST/Differential expression analysis/BEST_SingleGene_Immunotherapy_Expression_DPT_hgbkrK6Ffi/Plot_VanAllen.png]

# Lauss cohort 2017 CAR-T

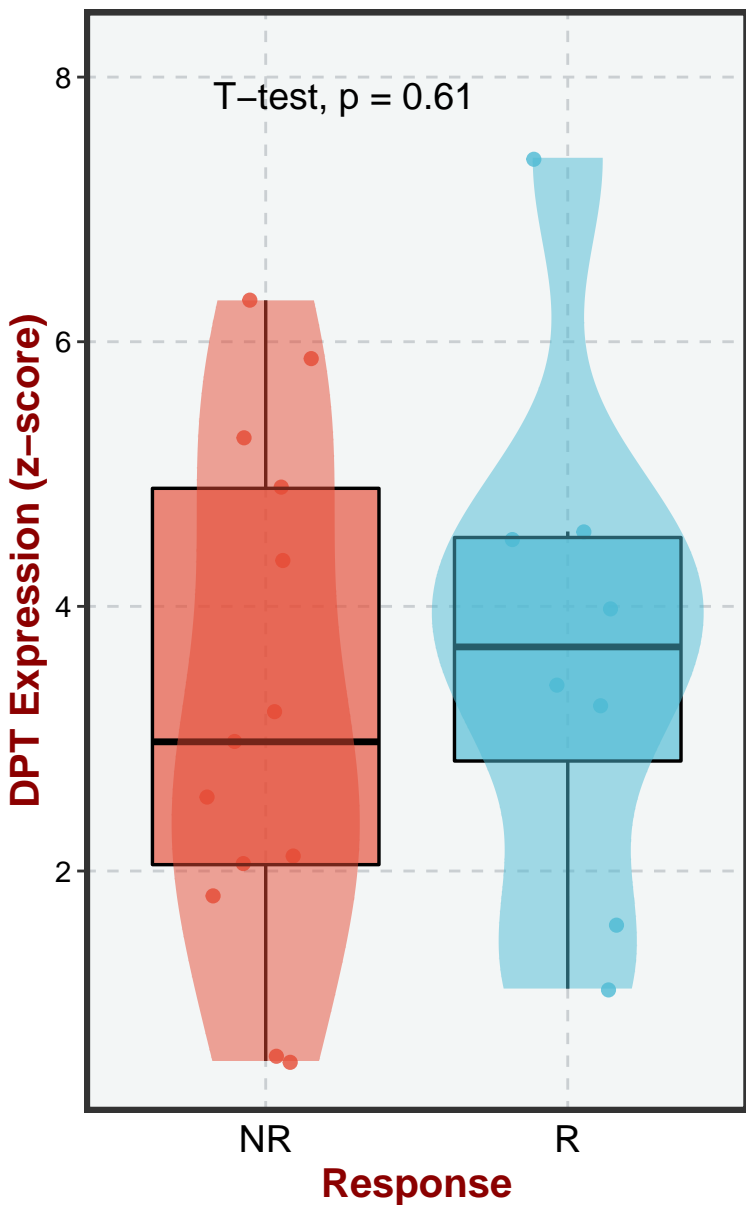

Supplement: Supplementary file 1 — Supplementary file1 (ZIP 153266 KB) [file 432_2023_5532_MOESM1_ESM.zip › Websites/BEST/Differential expression analysis/BEST_SingleGene_Immunotherapy_Expression_DPT_qaRU2h9pMf/Plot_GSE100797.pdf]

# Homet cohort 2019

## Anti-PD-1

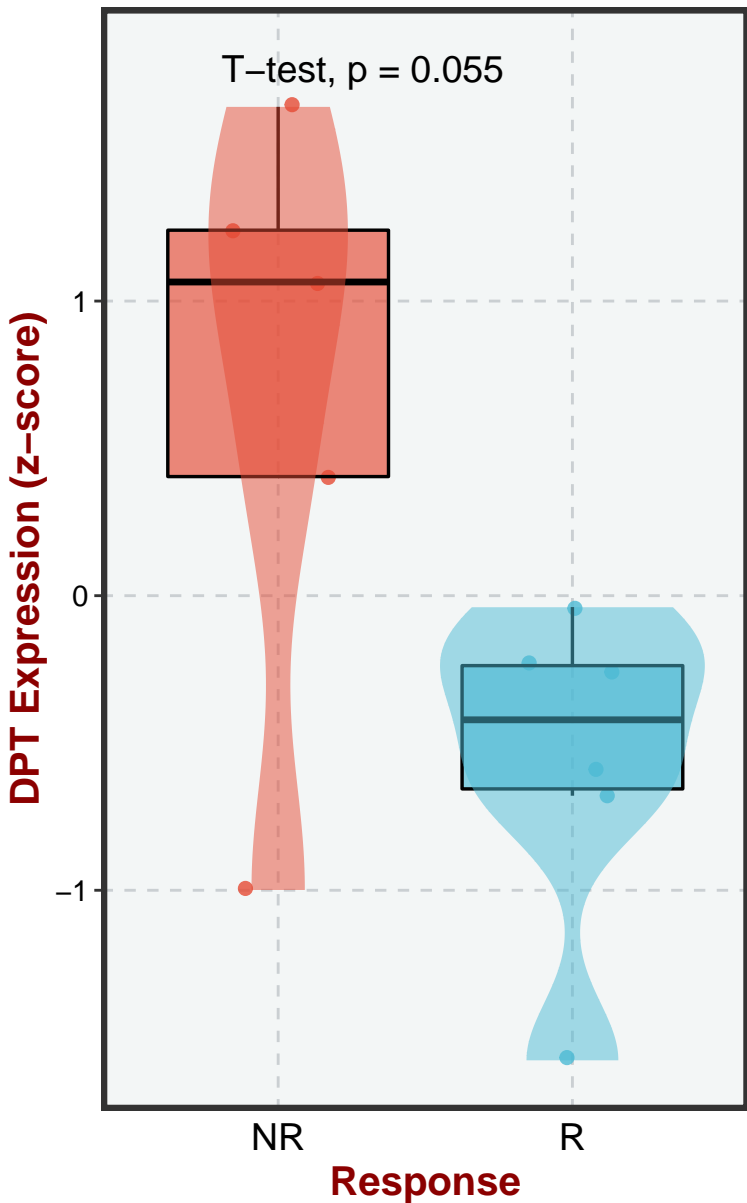

Supplement: Supplementary file 1 — Supplementary file1 (ZIP 153266 KB) [file 432_2023_5532_MOESM1_ESM.zip › Websites/BEST/Differential expression analysis/BEST_SingleGene_Immunotherapy_Expression_DPT_qaRU2h9pMf/Plot_GSE111636.pdf]

# Gao cohort 2018 Anti-PD-1/CTLA-4

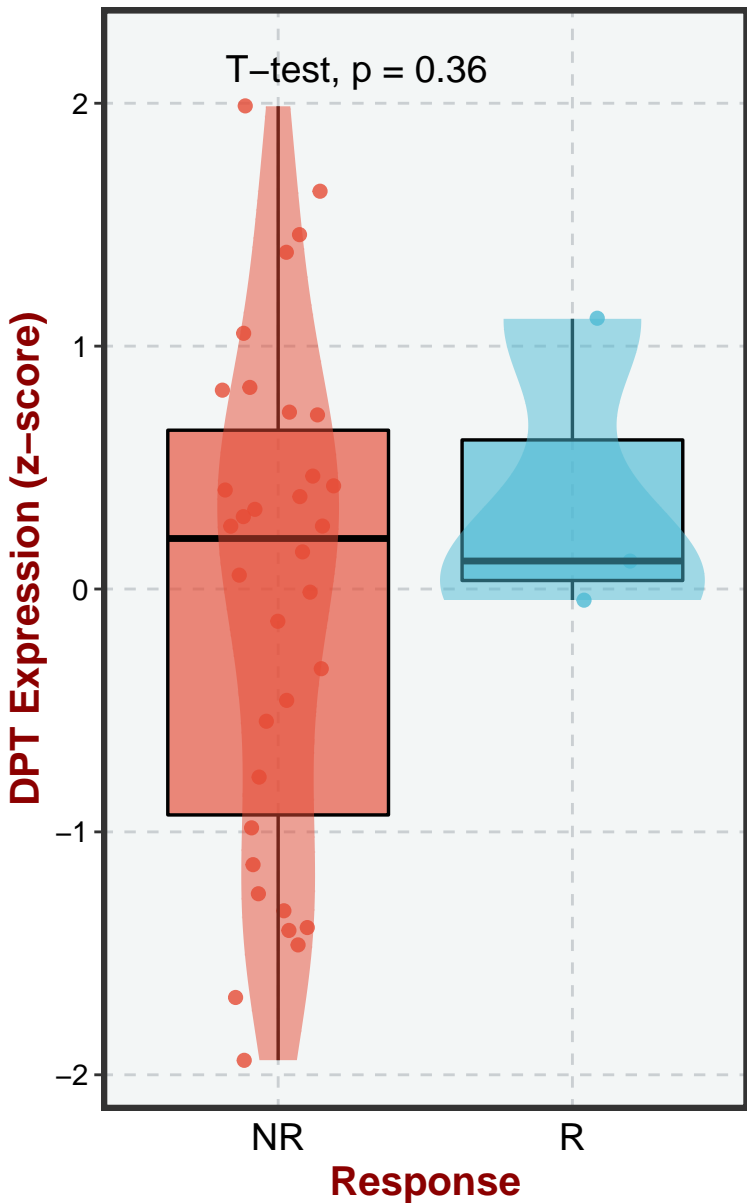

Supplement: Supplementary file 1 — Supplementary file1 (ZIP 153266 KB) [file 432_2023_5532_MOESM1_ESM.zip › Websites/BEST/Differential expression analysis/BEST_SingleGene_Immunotherapy_Expression_DPT_qaRU2h9pMf/Plot_GSE115821.pdf]

# Cho cohort 2020 Anti-PD-1/PD-L1

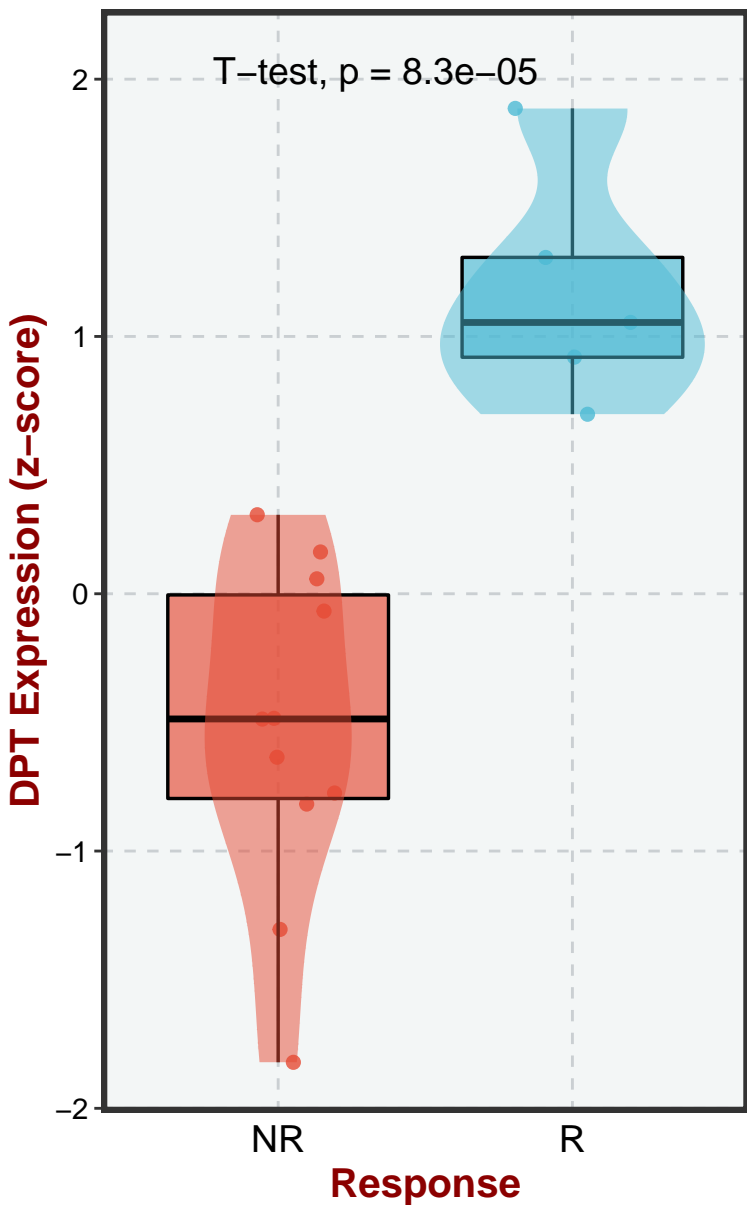

Supplement: Supplementary file 1 — Supplementary file1 (ZIP 153266 KB) [file 432_2023_5532_MOESM1_ESM.zip › Websites/BEST/Differential expression analysis/BEST_SingleGene_Immunotherapy_Expression_DPT_qaRU2h9pMf/Plot_GSE126044.pdf]

# Kim cohort 2019 Anti-PD-1/PD-L1

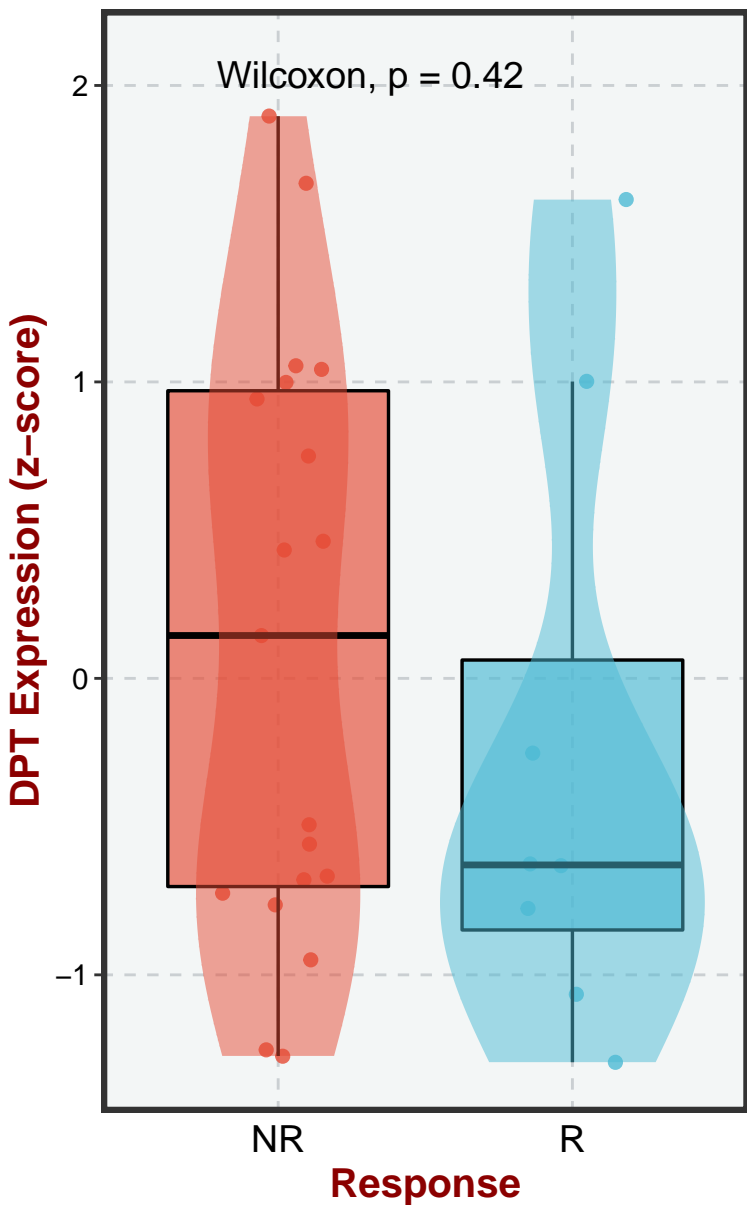

Supplement: Supplementary file 1 — Supplementary file1 (ZIP 153266 KB) [file 432_2023_5532_MOESM1_ESM.zip › Websites/BEST/Differential expression analysis/BEST_SingleGene_Immunotherapy_Expression_DPT_qaRU2h9pMf/Plot_GSE135222.pdf]

# Amato cohort 2020 Anti-PD-1

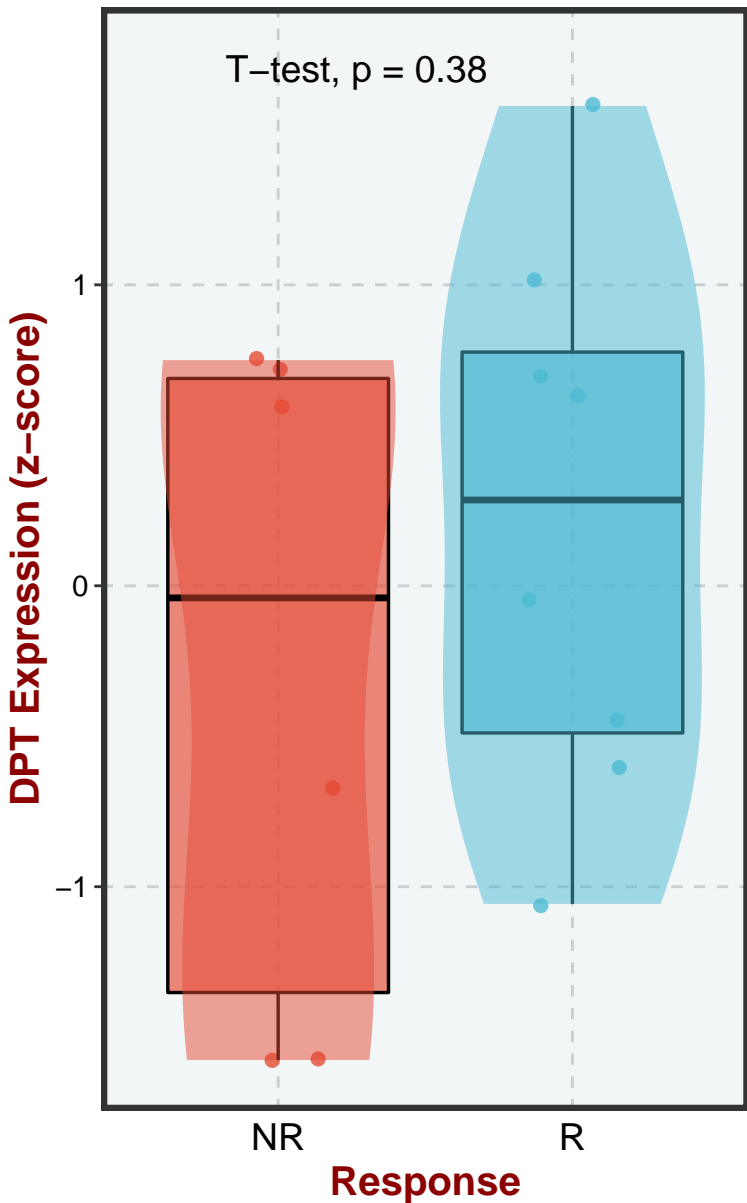

Supplement: Supplementary file 1 — Supplementary file1 (ZIP 153266 KB) [file 432_2023_5532_MOESM1_ESM.zip › Websites/BEST/Differential expression analysis/BEST_SingleGene_Immunotherapy_Expression_DPT_qaRU2h9pMf/Plot_GSE145996.pdf]

# Van cohort 2021 Anti-PD-L1

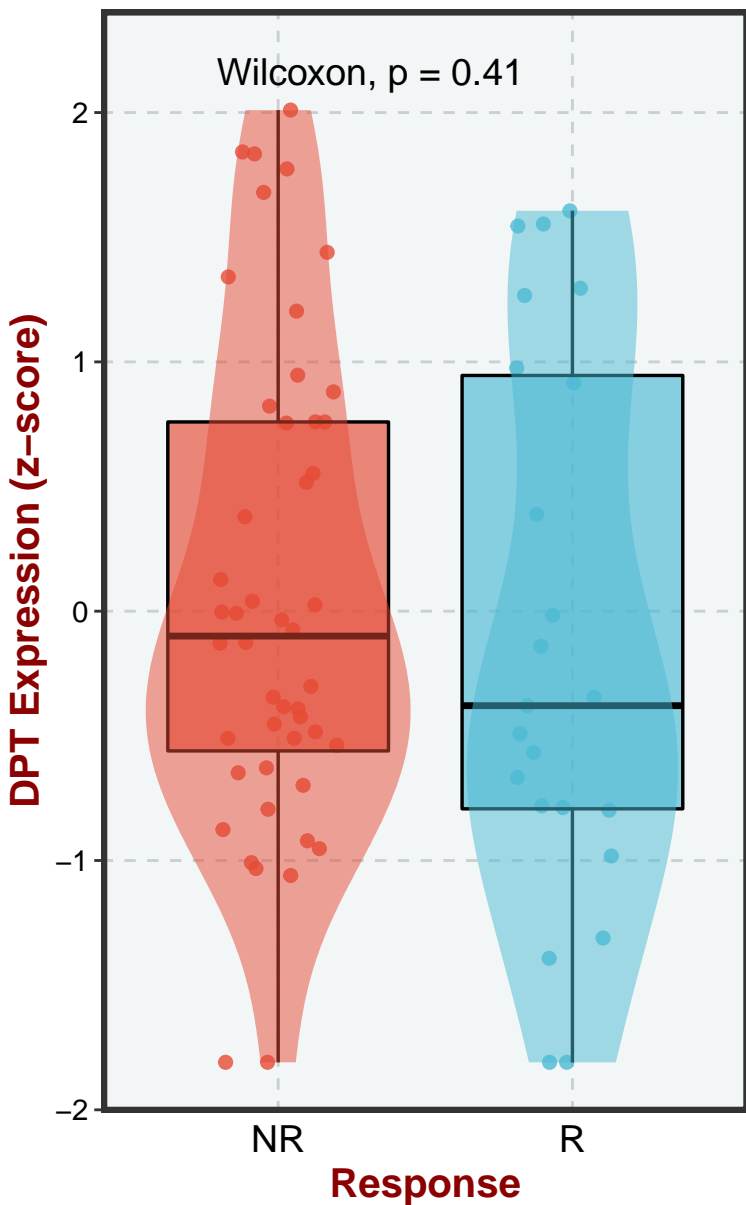

Supplement: Supplementary file 1 — Supplementary file1 (ZIP 153266 KB) [file 432_2023_5532_MOESM1_ESM.zip › Websites/BEST/Differential expression analysis/BEST_SingleGene_Immunotherapy_Expression_DPT_qaRU2h9pMf/Plot_GSE165252.pdf]

# Wolf cohort 2021 Anti-PD-L1

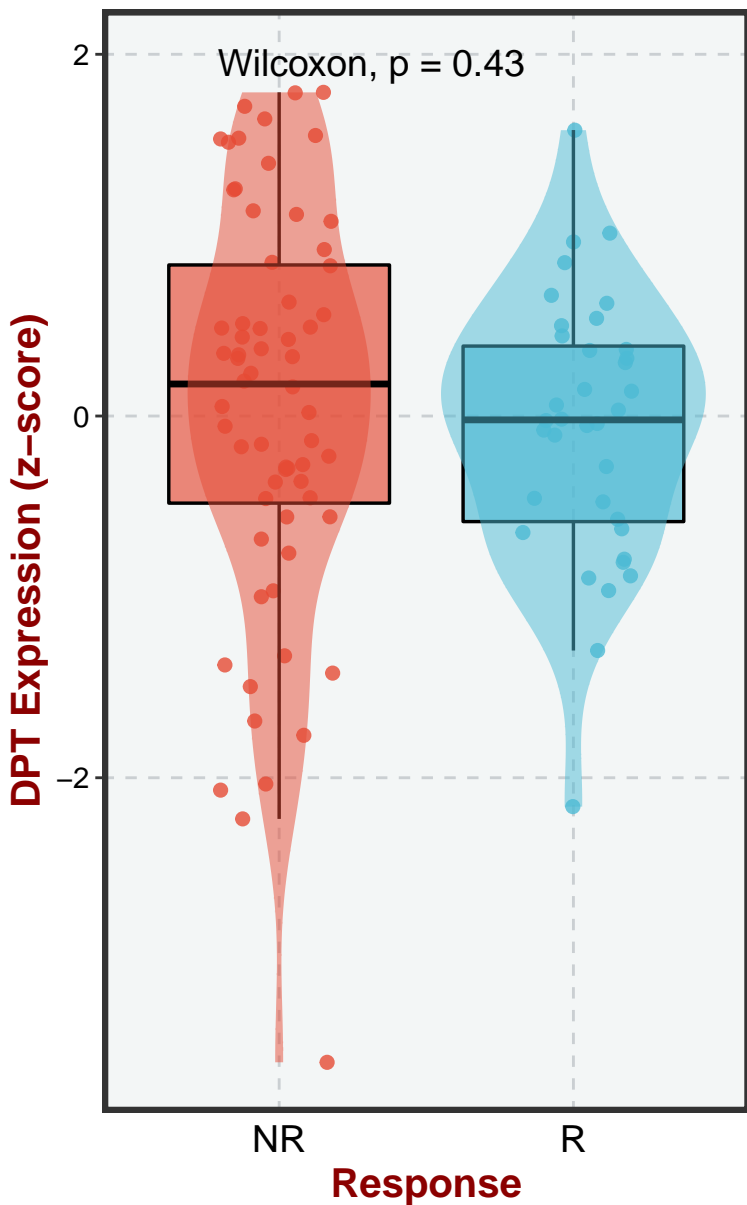

Supplement: Supplementary file 1 — Supplementary file1 (ZIP 153266 KB) [file 432_2023_5532_MOESM1_ESM.zip › Websites/BEST/Differential expression analysis/BEST_SingleGene_Immunotherapy_Expression_DPT_qaRU2h9pMf/Plot_GSE173839.pdf]

# Dizier cohort 2013 Anti-MAGE-A3

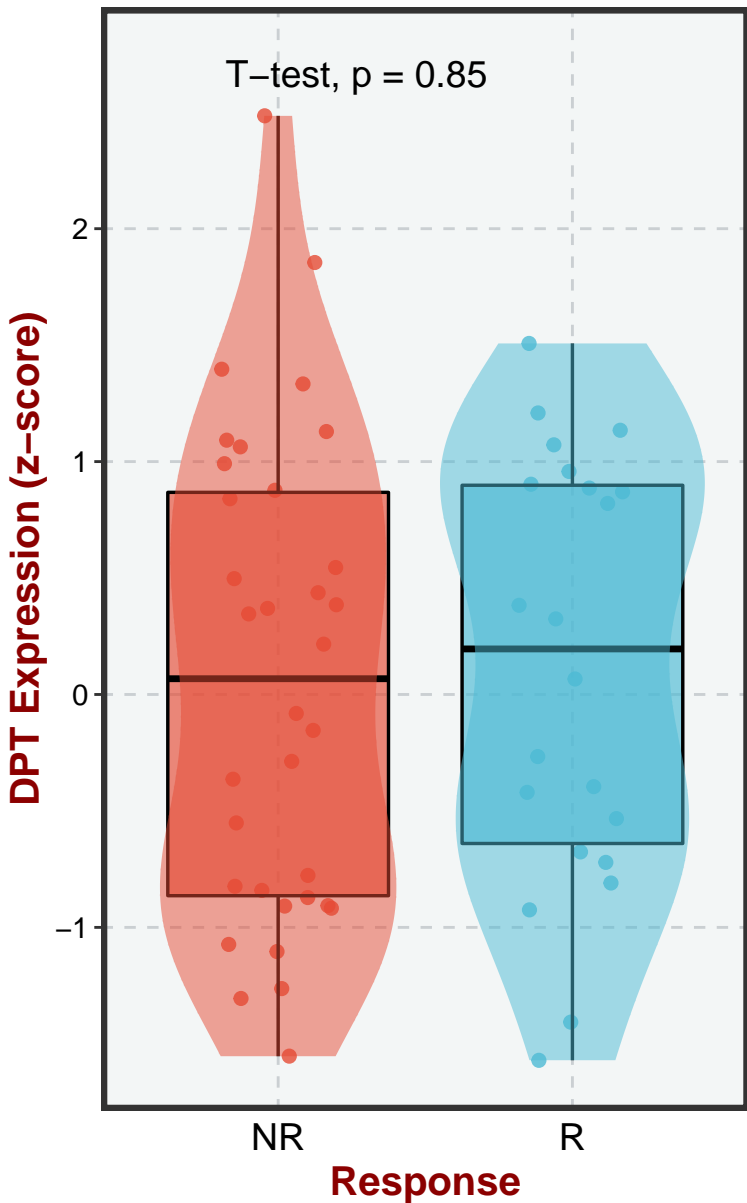

Supplement: Supplementary file 1 — Supplementary file1 (ZIP 153266 KB) [file 432_2023_5532_MOESM1_ESM.zip › Websites/BEST/Differential expression analysis/BEST_SingleGene_Immunotherapy_Expression_DPT_qaRU2h9pMf/Plot_GSE35640.pdf]

# Ascierto cohort 2016 Anti-PD-1

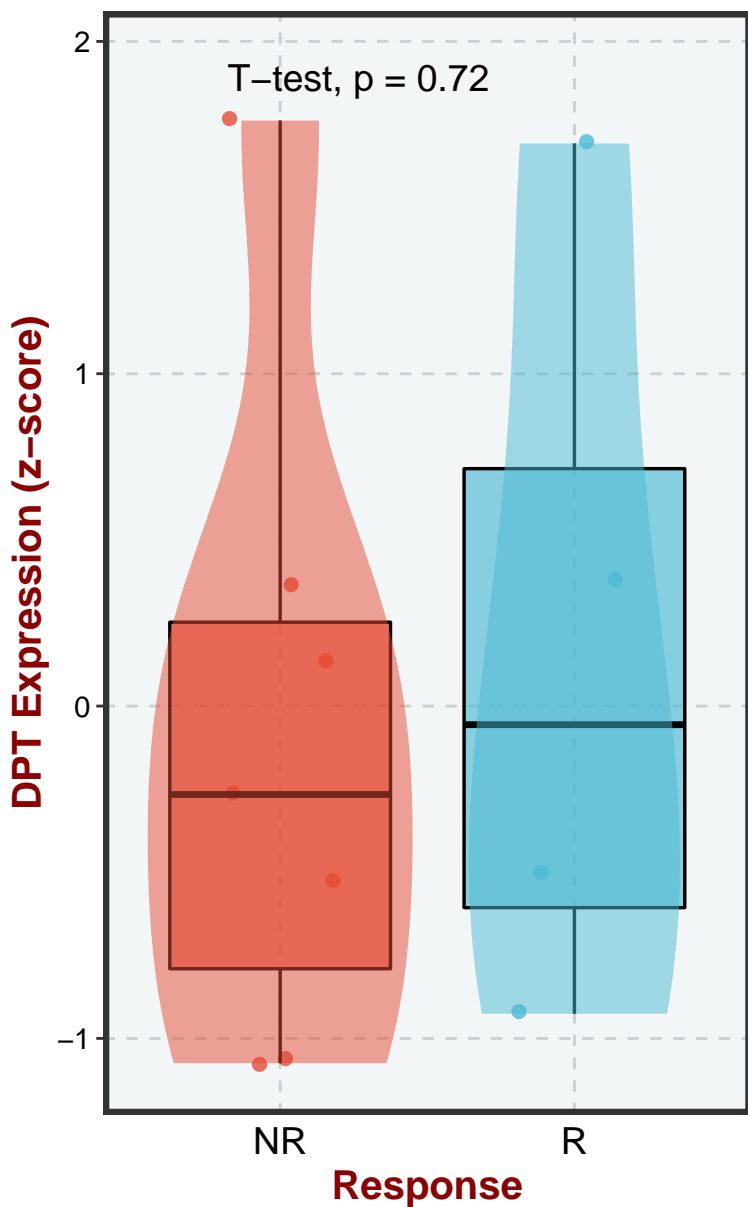

Supplement: Supplementary file 1 — Supplementary file1 (ZIP 153266 KB) [file 432_2023_5532_MOESM1_ESM.zip › Websites/BEST/Differential expression analysis/BEST_SingleGene_Immunotherapy_Expression_DPT_qaRU2h9pMf/Plot_GSE67501.pdf]

# Hugo cohort 2016

## Anti-PD-1

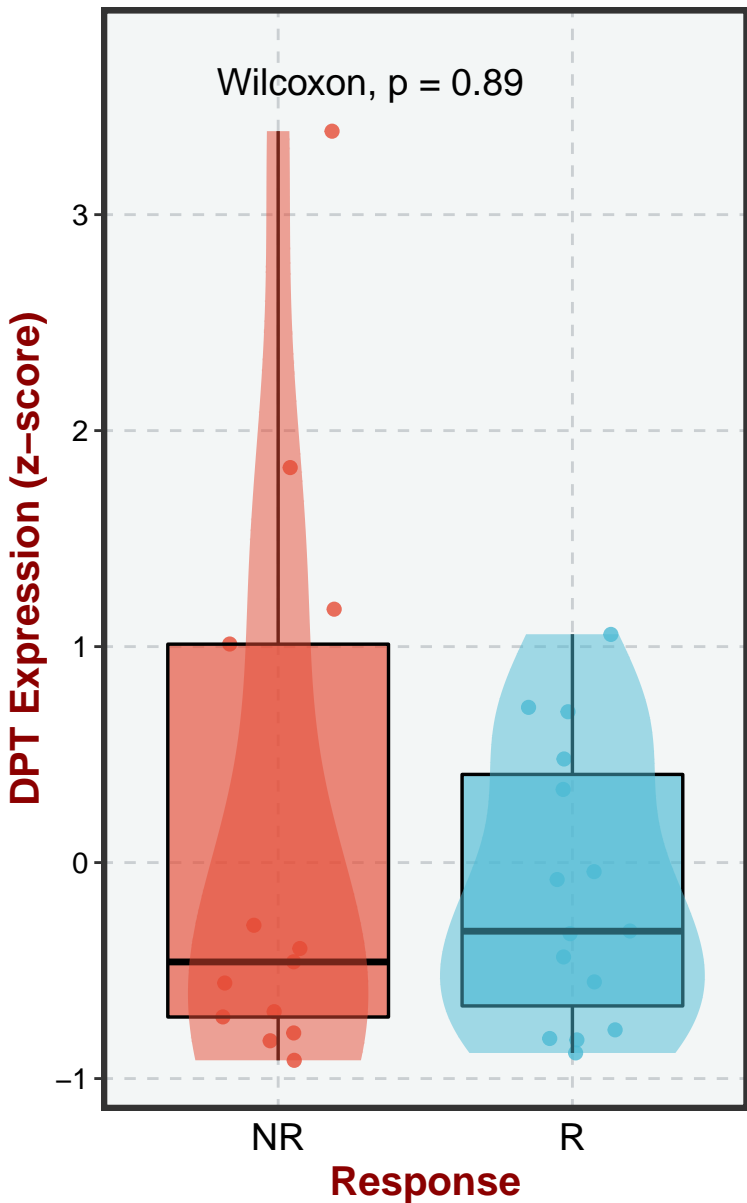

Supplement: Supplementary file 1 — Supplementary file1 (ZIP 153266 KB) [file 432_2023_5532_MOESM1_ESM.zip › Websites/BEST/Differential expression analysis/BEST_SingleGene_Immunotherapy_Expression_DPT_qaRU2h9pMf/Plot_GSE78220.pdf]

# Riaz cohort 2018

## Anti-PD-1/CTLA-4

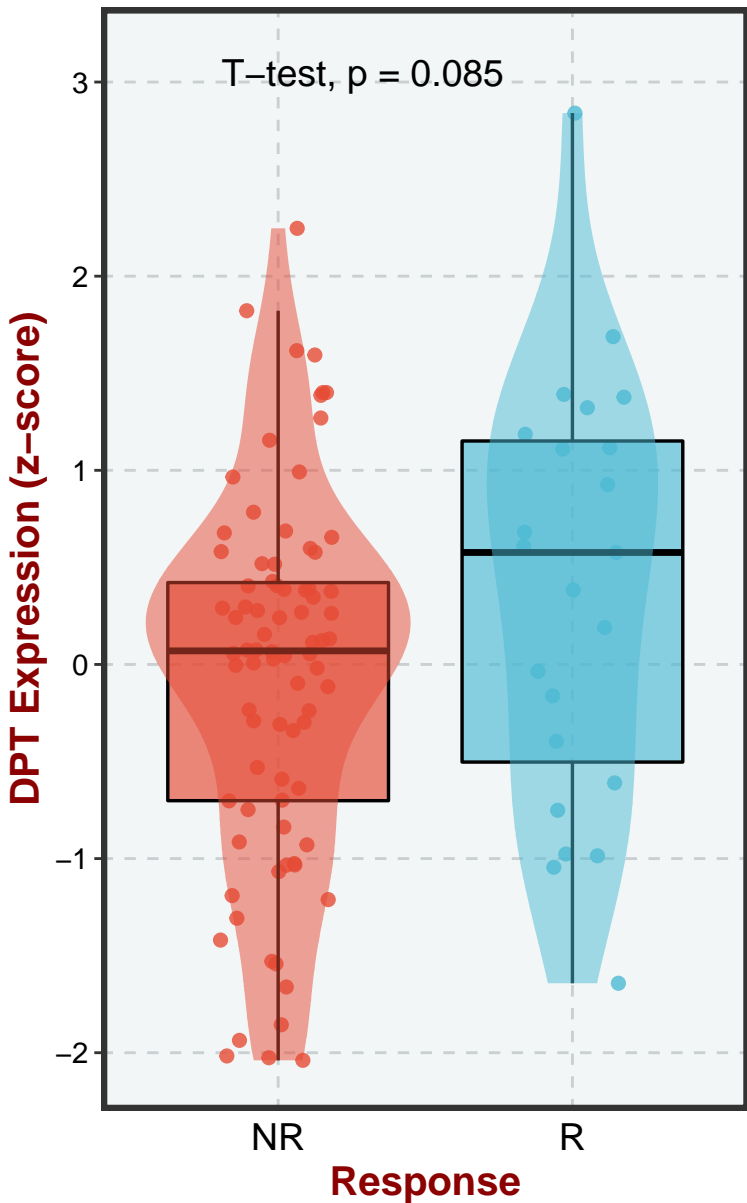

Supplement: Supplementary file 1 — Supplementary file1 (ZIP 153266 KB) [file 432_2023_5532_MOESM1_ESM.zip › Websites/BEST/Differential expression analysis/BEST_SingleGene_Immunotherapy_Expression_DPT_qaRU2h9pMf/Plot_GSE91061.pdf]

# IMvigor210 cohort 2018

## Anti-PD-L1

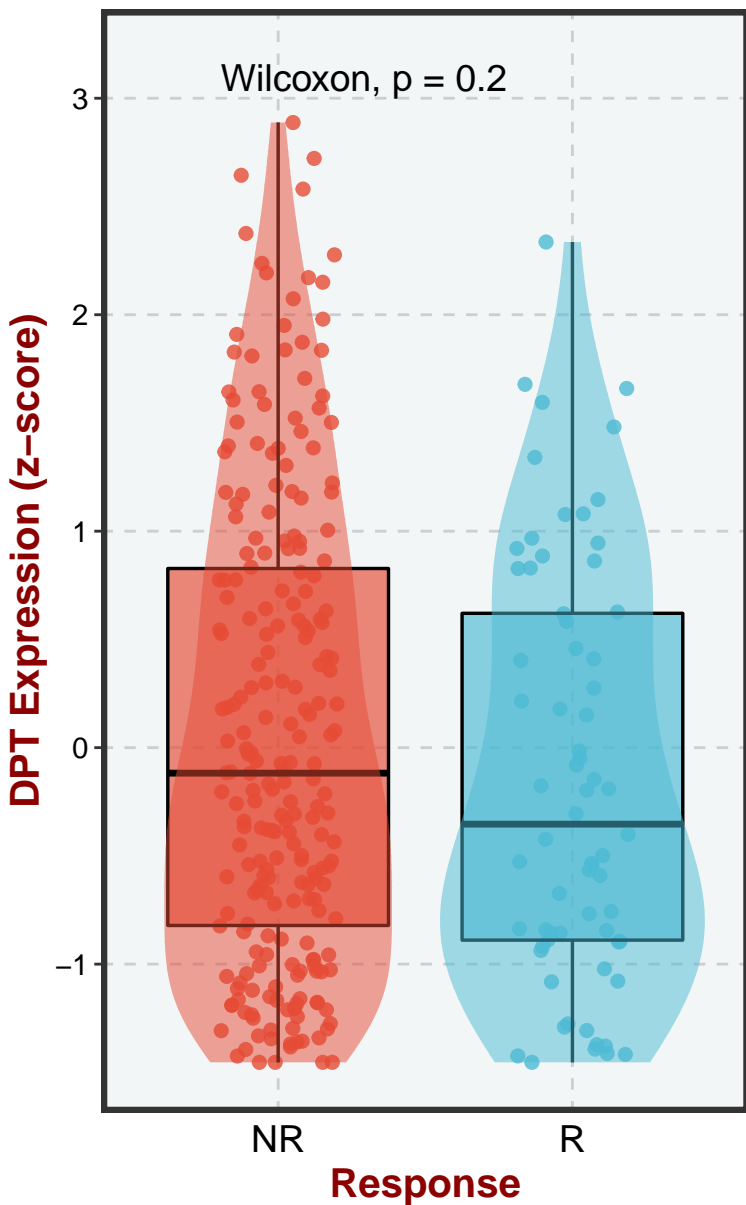

Supplement: Supplementary file 1 — Supplementary file1 (ZIP 153266 KB) [file 432_2023_5532_MOESM1_ESM.zip › Websites/BEST/Differential expression analysis/BEST_SingleGene_Immunotherapy_Expression_DPT_qaRU2h9pMf/Plot_IMvigor210.pdf]

# Nathanson cohort 2017

## Anti-CTLA-4

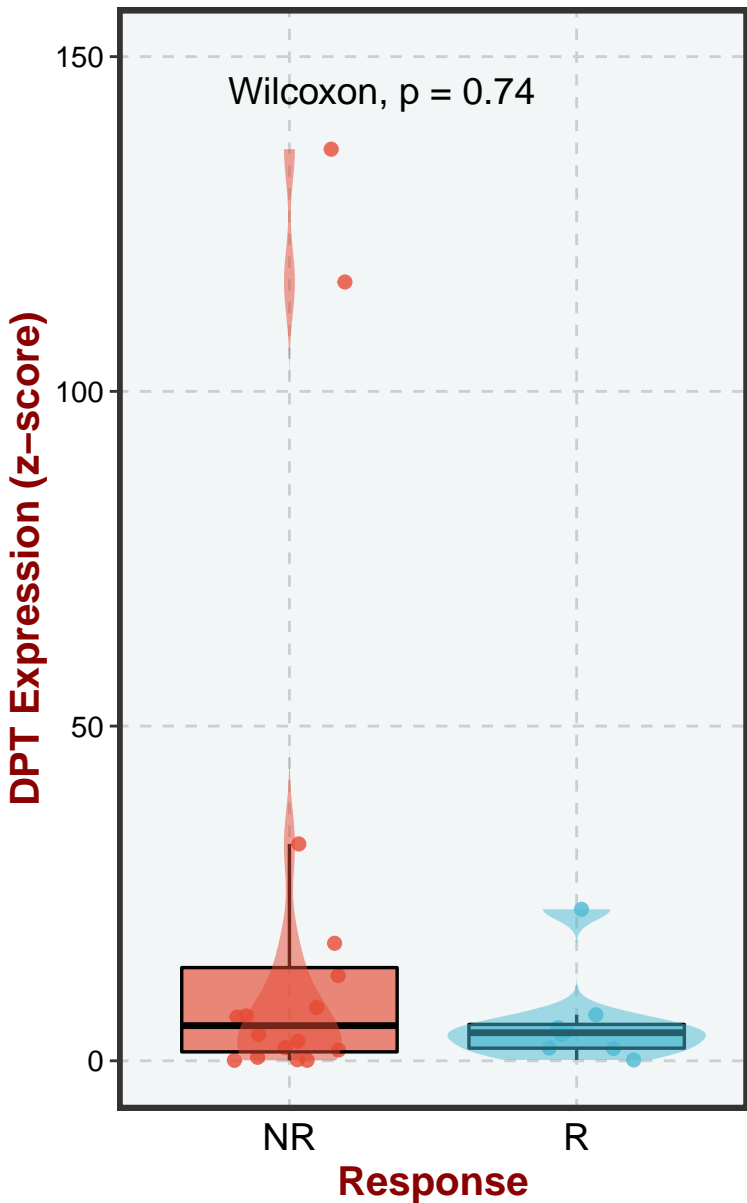

Supplement: Supplementary file 1 — Supplementary file1 (ZIP 153266 KB) [file 432_2023_5532_MOESM1_ESM.zip › Websites/BEST/Differential expression analysis/BEST_SingleGene_Immunotherapy_Expression_DPT_qaRU2h9pMf/Plot_Nathanson.pdf]

# VanAllen cohort 2015 Anti-CTLA-4

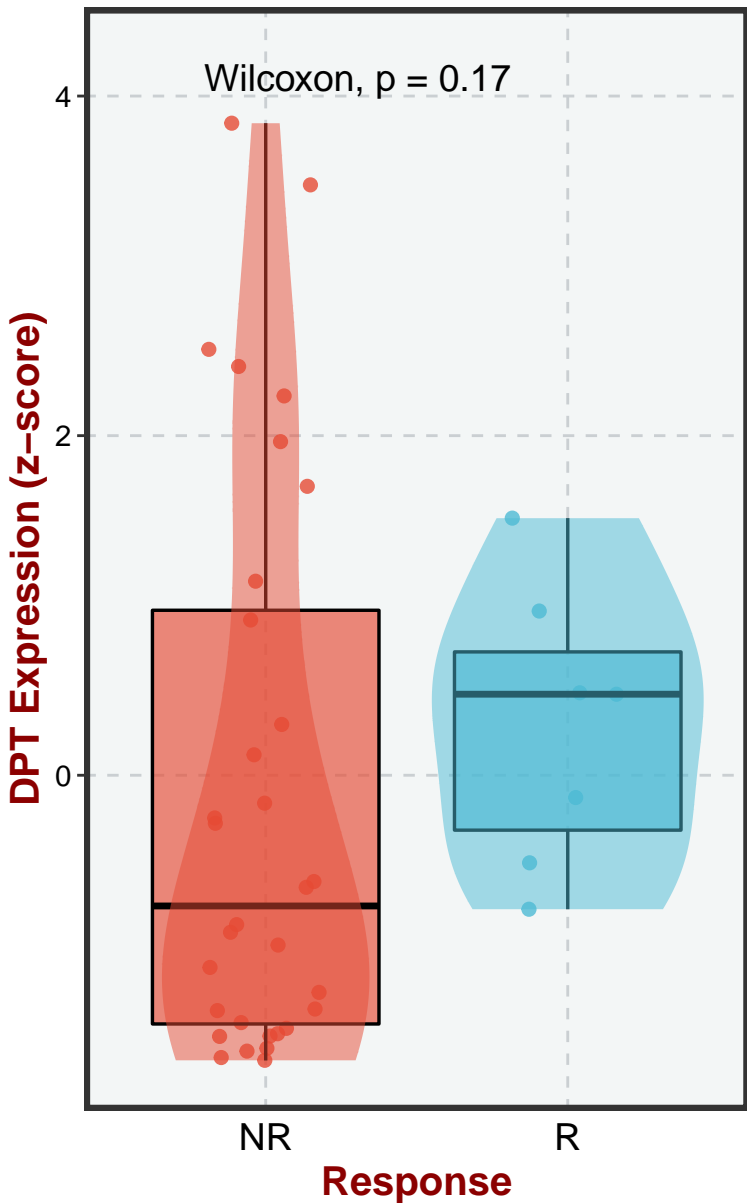

Supplement: Supplementary file 1 — Supplementary file1 (ZIP 153266 KB) [file 432_2023_5532_MOESM1_ESM.zip › Websites/BEST/Differential expression analysis/BEST_SingleGene_Immunotherapy_Expression_DPT_qaRU2h9pMf/Plot_VanAllen.pdf]

# Lauss cohort 2017 (CAR-T)

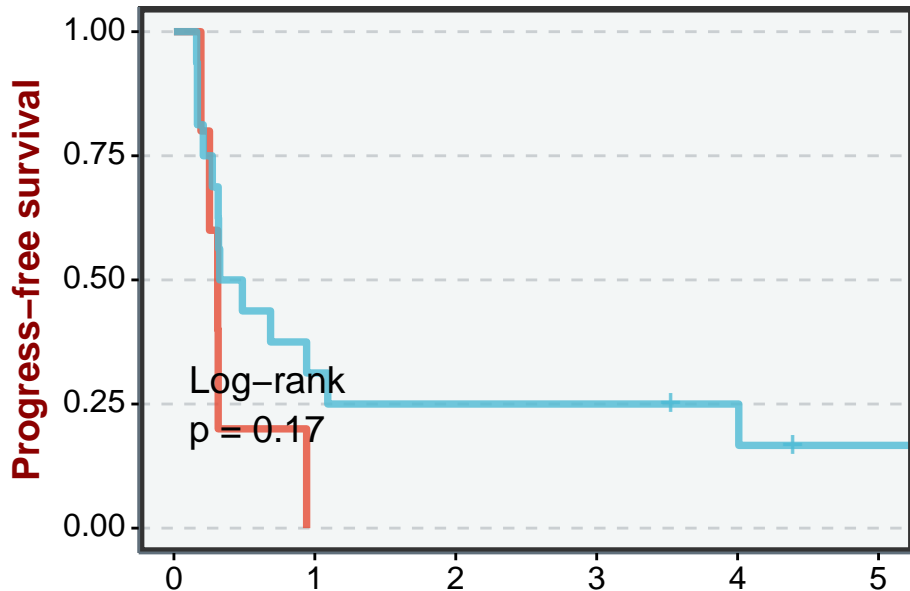

## Number at risk

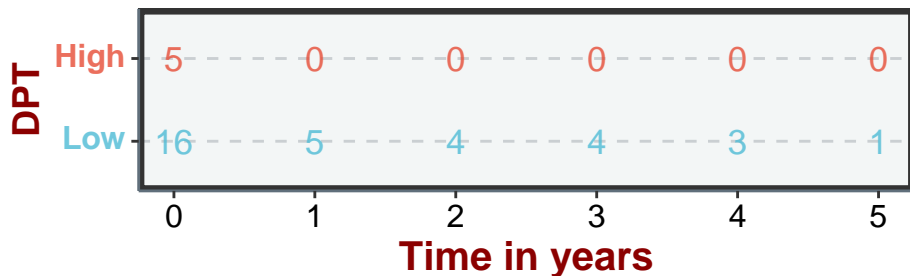

Supplement: Supplementary file 1 — Supplementary file1 (ZIP 153266 KB) [file 432_2023_5532_MOESM1_ESM.zip › Websites/BEST/immunotherapy prognosis/BEST_SingleGene_Immunotherapy_Survival_DPT_YkIfJQSPjo/Plot_GSE100797.pdf]

# Cho cohort 2020 (Anti-PD-1/PD-L1)

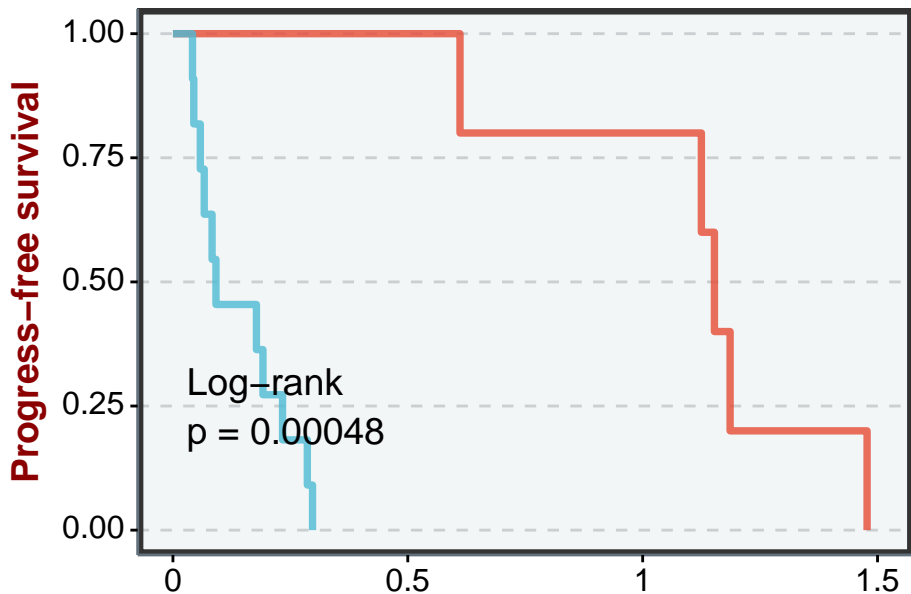

## Number at risk

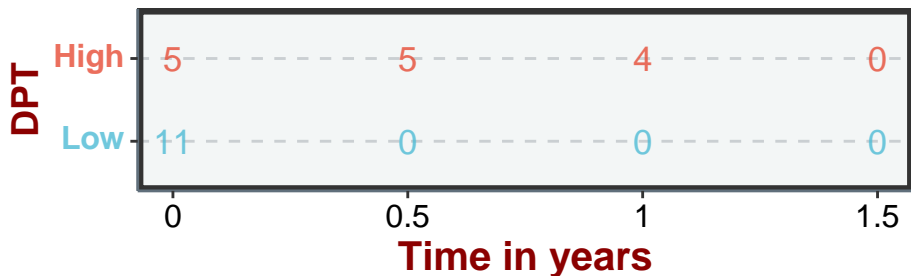

Supplement: Supplementary file 1 — Supplementary file1 (ZIP 153266 KB) [file 432_2023_5532_MOESM1_ESM.zip › Websites/BEST/immunotherapy prognosis/BEST_SingleGene_Immunotherapy_Survival_DPT_YkIfJQSPjo/Plot_GSE126044.pdf]

# Kim cohort 2019 (Anti-PD-1/PD-L1)

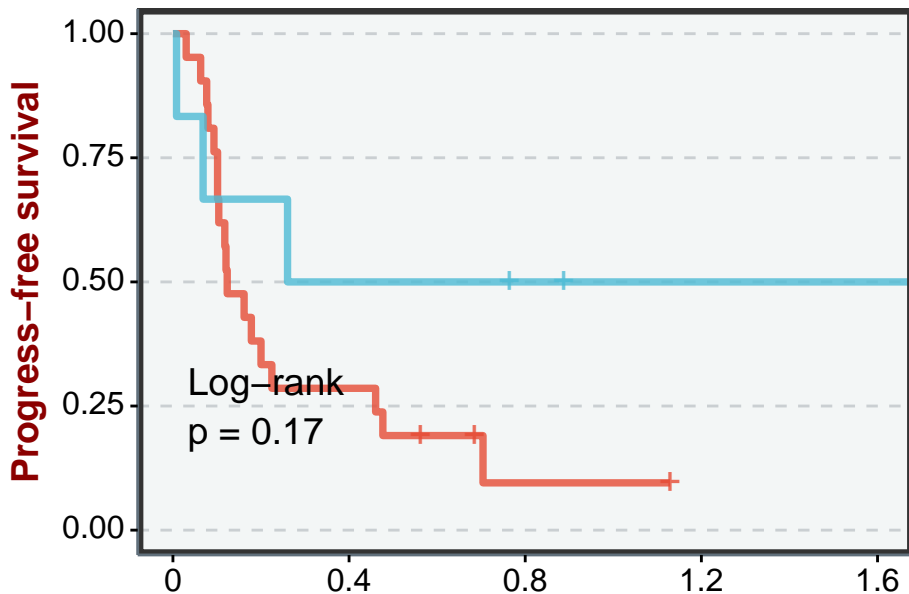

## Number at risk

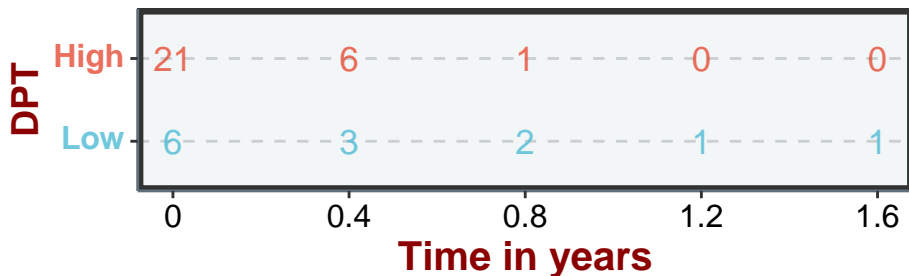

Supplement: Supplementary file 1 — Supplementary file1 (ZIP 153266 KB) [file 432_2023_5532_MOESM1_ESM.zip › Websites/BEST/immunotherapy prognosis/BEST_SingleGene_Immunotherapy_Survival_DPT_YkIfJQSPjo/Plot_GSE135222.pdf]

# Hugo cohort 2016 (Anti-PD-1)

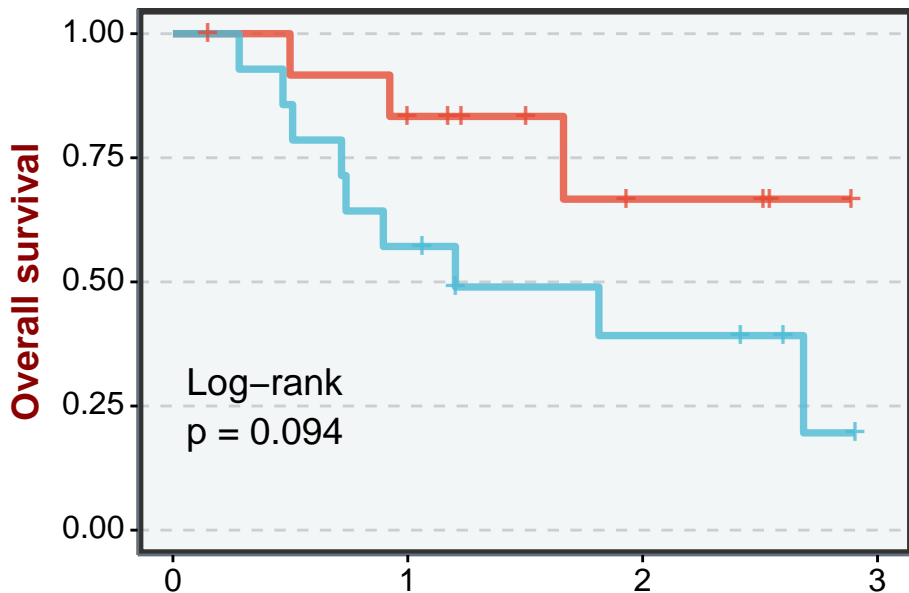

## Number at risk

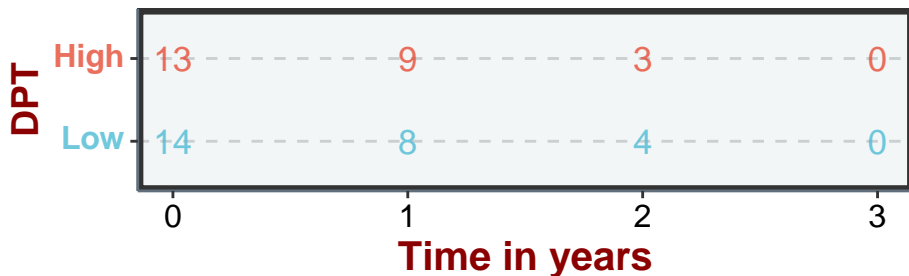

Supplement: Supplementary file 1 — Supplementary file1 (ZIP 153266 KB) [file 432_2023_5532_MOESM1_ESM.zip › Websites/BEST/immunotherapy prognosis/BEST_SingleGene_Immunotherapy_Survival_DPT_YkIfJQSPjo/Plot_GSE78220.pdf]

# IMvigor210 cohort 2018 (Anti-PD-L1)

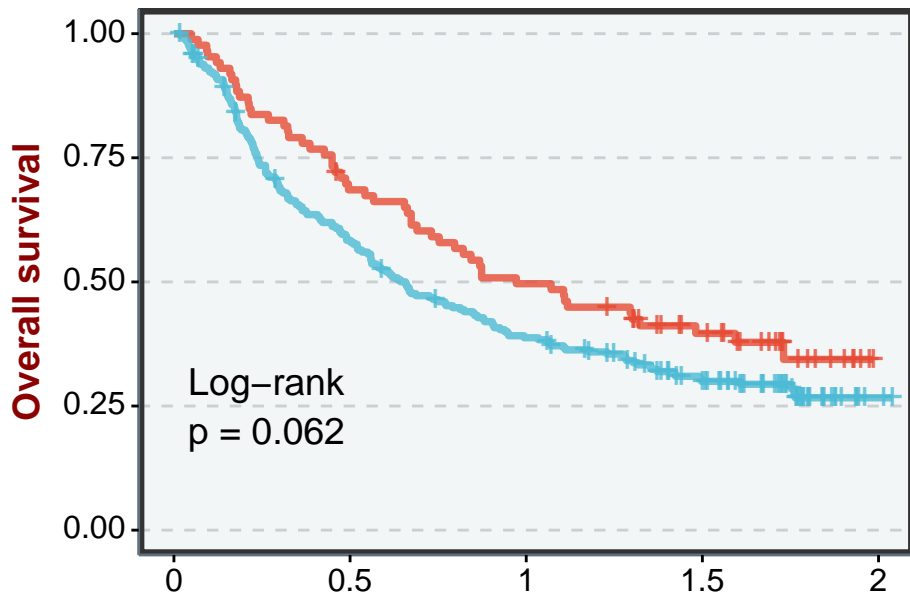

## Number at risk

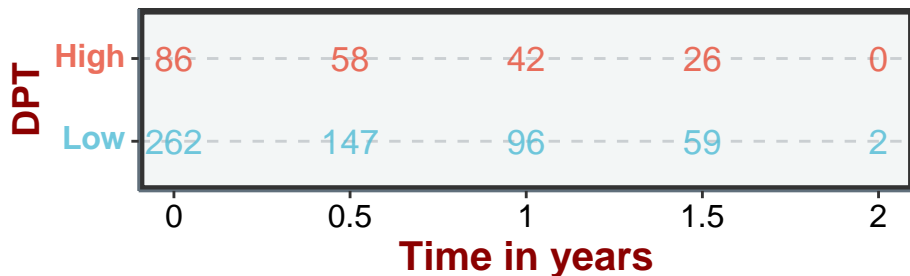

Supplement: Supplementary file 1 — Supplementary file1 (ZIP 153266 KB) [file 432_2023_5532_MOESM1_ESM.zip › Websites/BEST/immunotherapy prognosis/BEST_SingleGene_Immunotherapy_Survival_DPT_YkIfJQSPjo/Plot_IMvigor210.pdf]

# Nathanson cohort 2017 (Anti-CTLA-4)

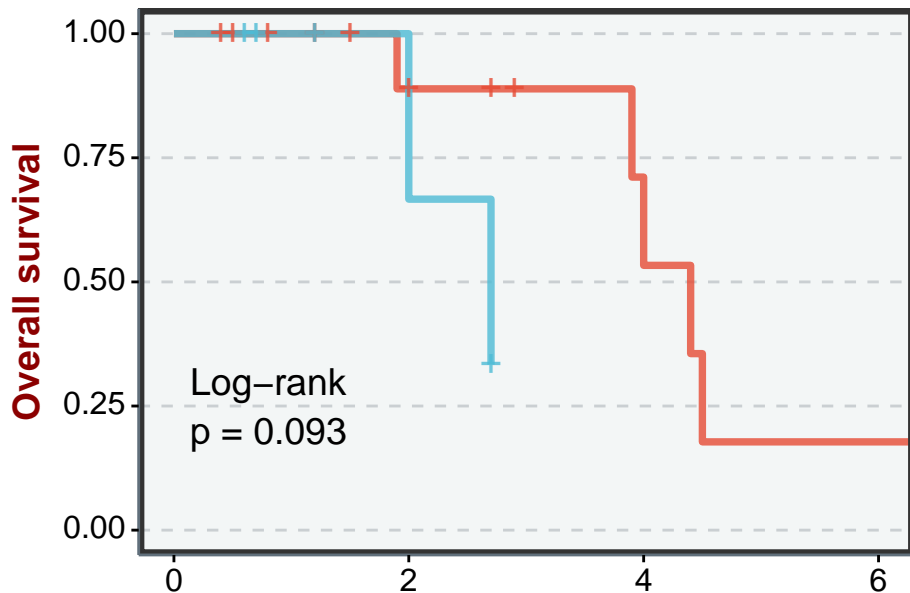

## Number at risk

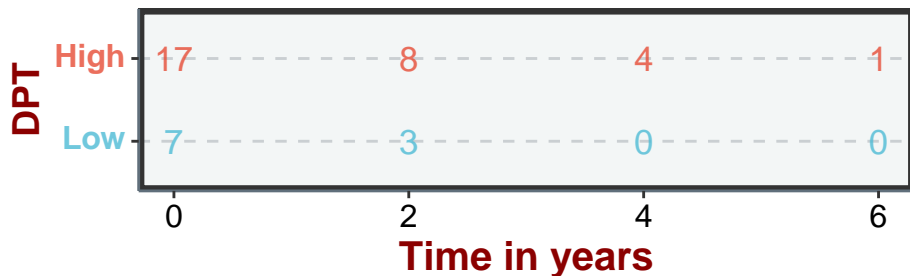

Supplement: Supplementary file 1 — Supplementary file1 (ZIP 153266 KB) [file 432_2023_5532_MOESM1_ESM.zip › Websites/BEST/immunotherapy prognosis/BEST_SingleGene_Immunotherapy_Survival_DPT_YkIfJQSPjo/Plot_Nathanson.pdf]

# VanAllen cohort 2015 (Anti-CTLA-4)

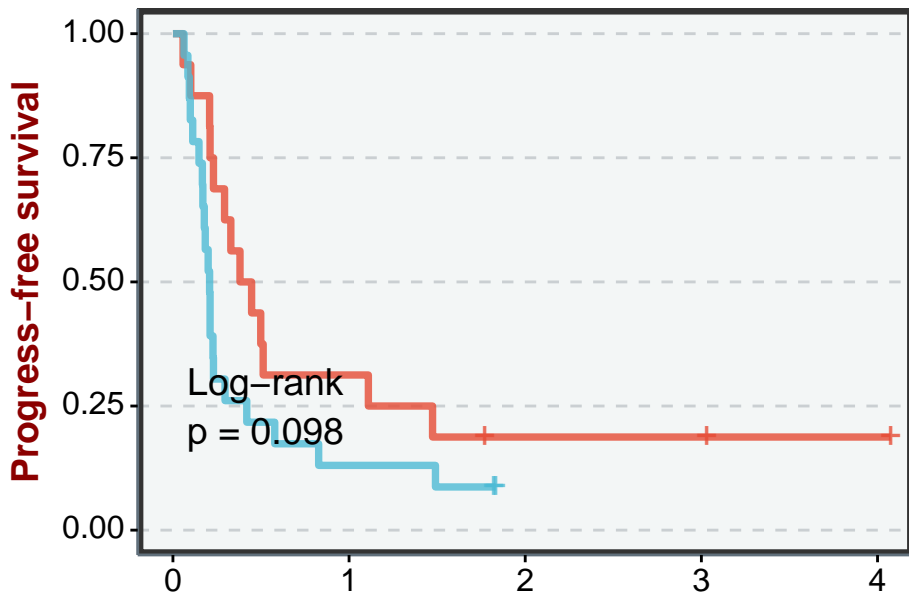

## Number at risk

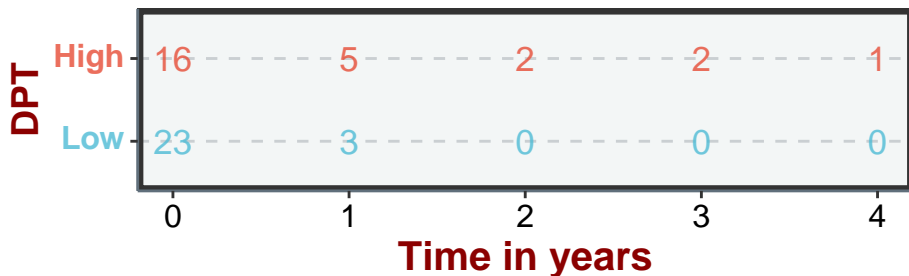

Supplement: Supplementary file 1 — Supplementary file1 (ZIP 153266 KB) [file 432_2023_5532_MOESM1_ESM.zip › Websites/BEST/immunotherapy prognosis/BEST_SingleGene_Immunotherapy_Survival_DPT_YkIfJQSPjo/Plot_VanAllen.pdf]

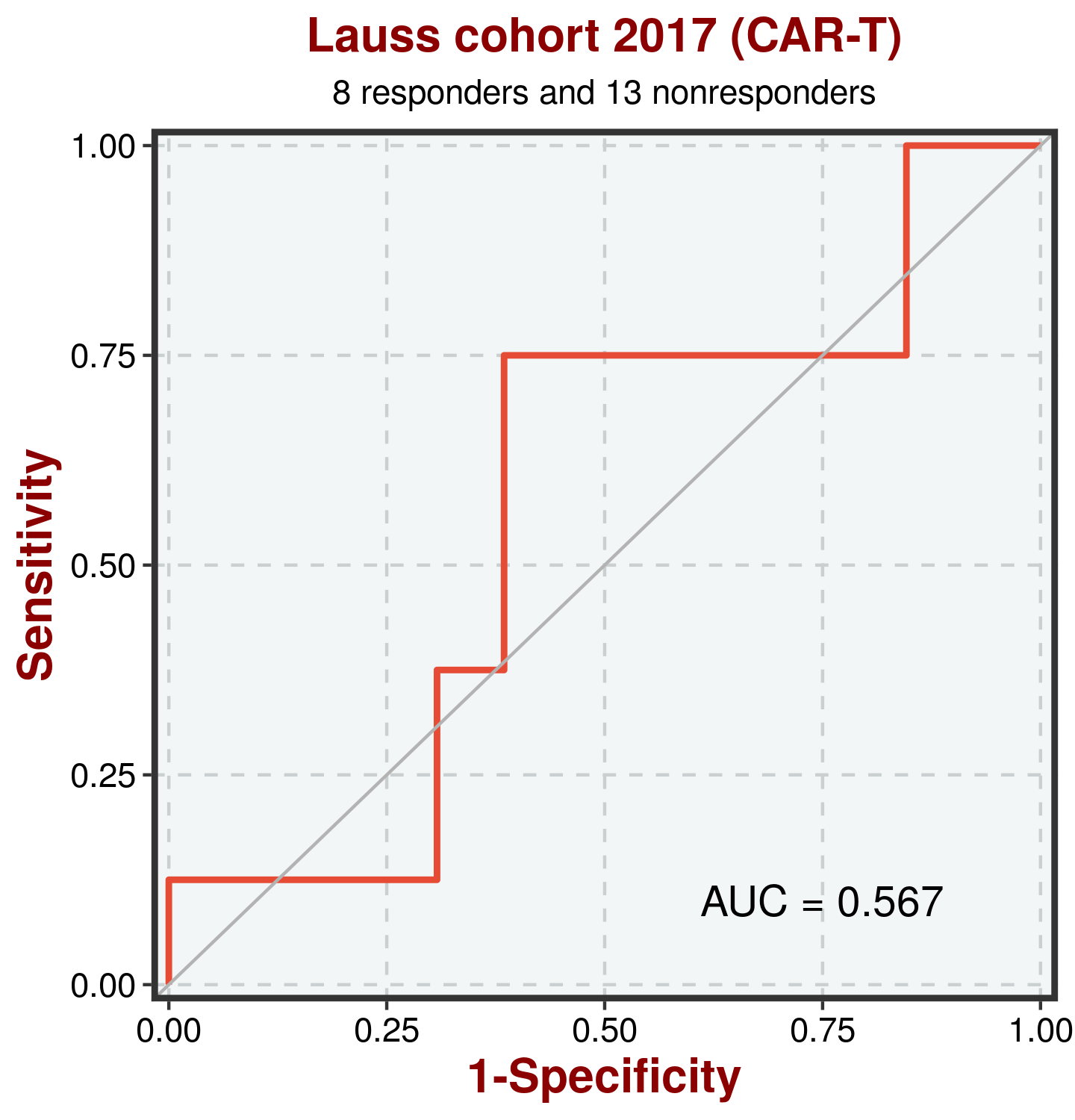

Supplement: Supplementary file 1 — Supplementary file1 (ZIP 153266 KB) [file 432_2023_5532_MOESM1_ESM.zip › Websites/BEST/response prediction/BEST_SingleGene_Immunotherapy_ROC_DPT_WTdaIe2Umg/Plot_GSE100797.png]

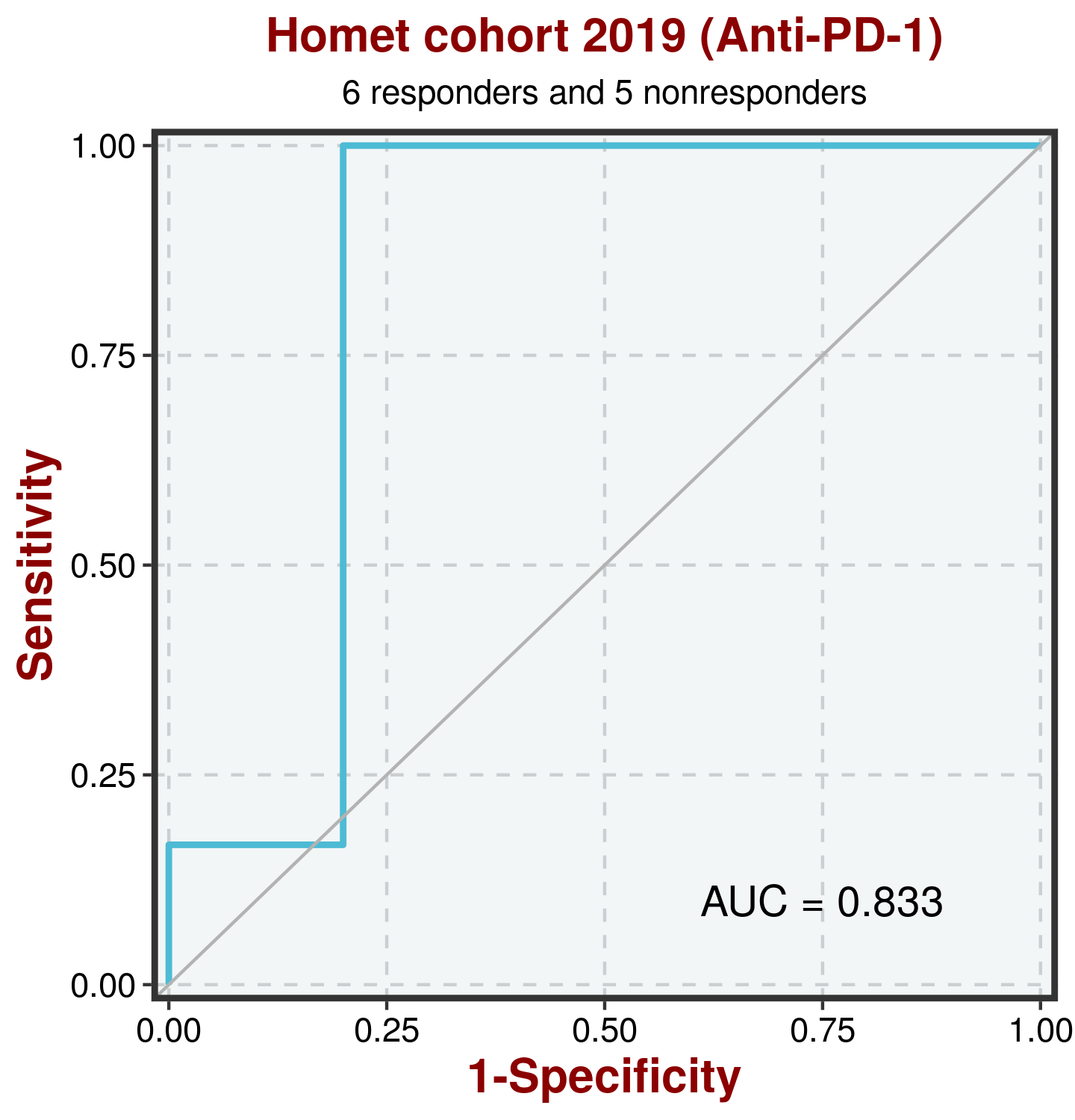

Supplement: Supplementary file 1 — Supplementary file1 (ZIP 153266 KB) [file 432_2023_5532_MOESM1_ESM.zip › Websites/BEST/response prediction/BEST_SingleGene_Immunotherapy_ROC_DPT_WTdaIe2Umg/Plot_GSE111636.png]

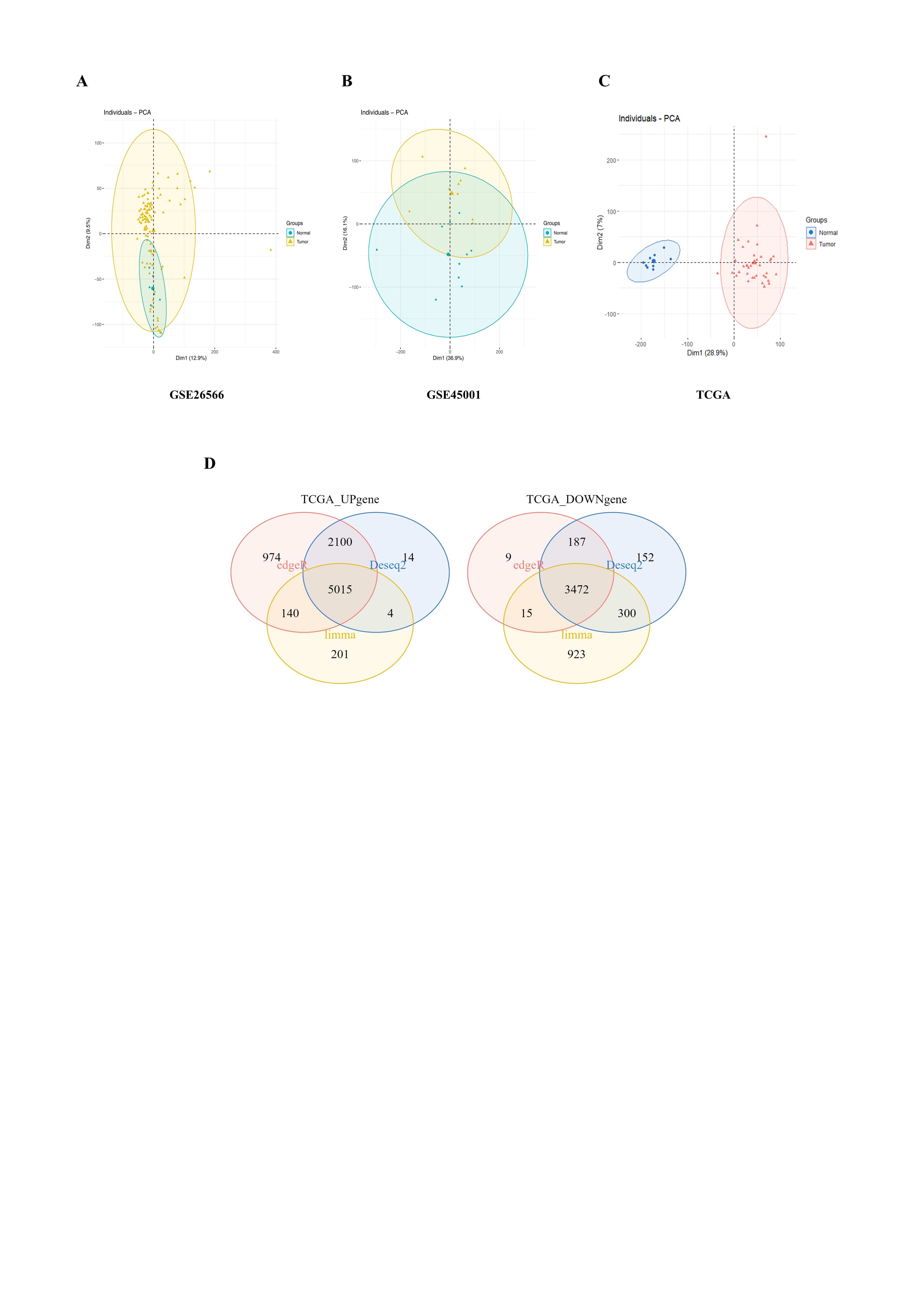

Supplement: Supplementary file 2 — Supplementary file2 (ZIP 4862 KB) [file 432_2023_5532_MOESM2_ESM.zip › SupplementaryFig. 1.jpg]

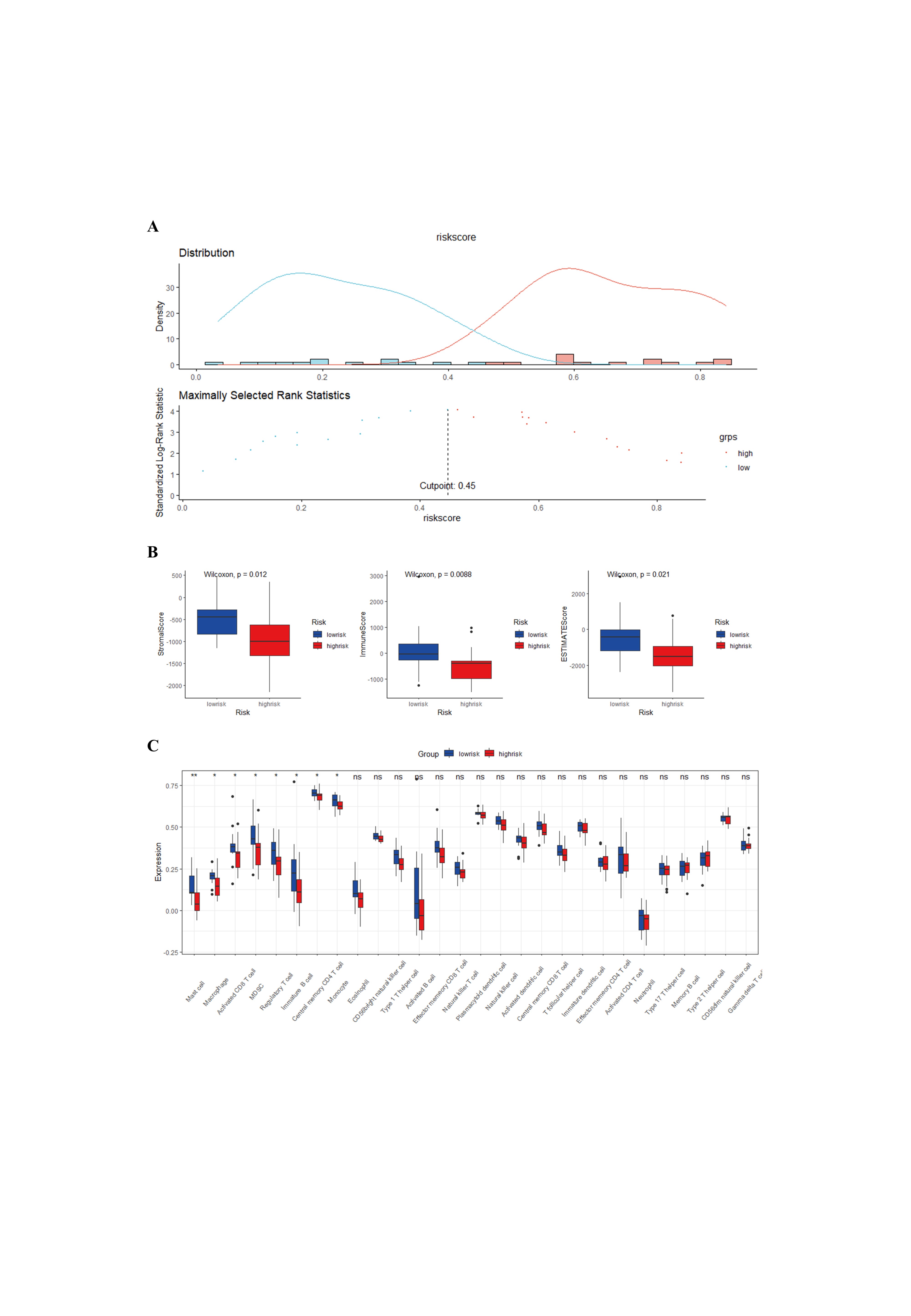

Supplement: Supplementary file 2 — Supplementary file2 (ZIP 4862 KB) [file 432_2023_5532_MOESM2_ESM.zip › SupplementaryFig. 2.jpg]

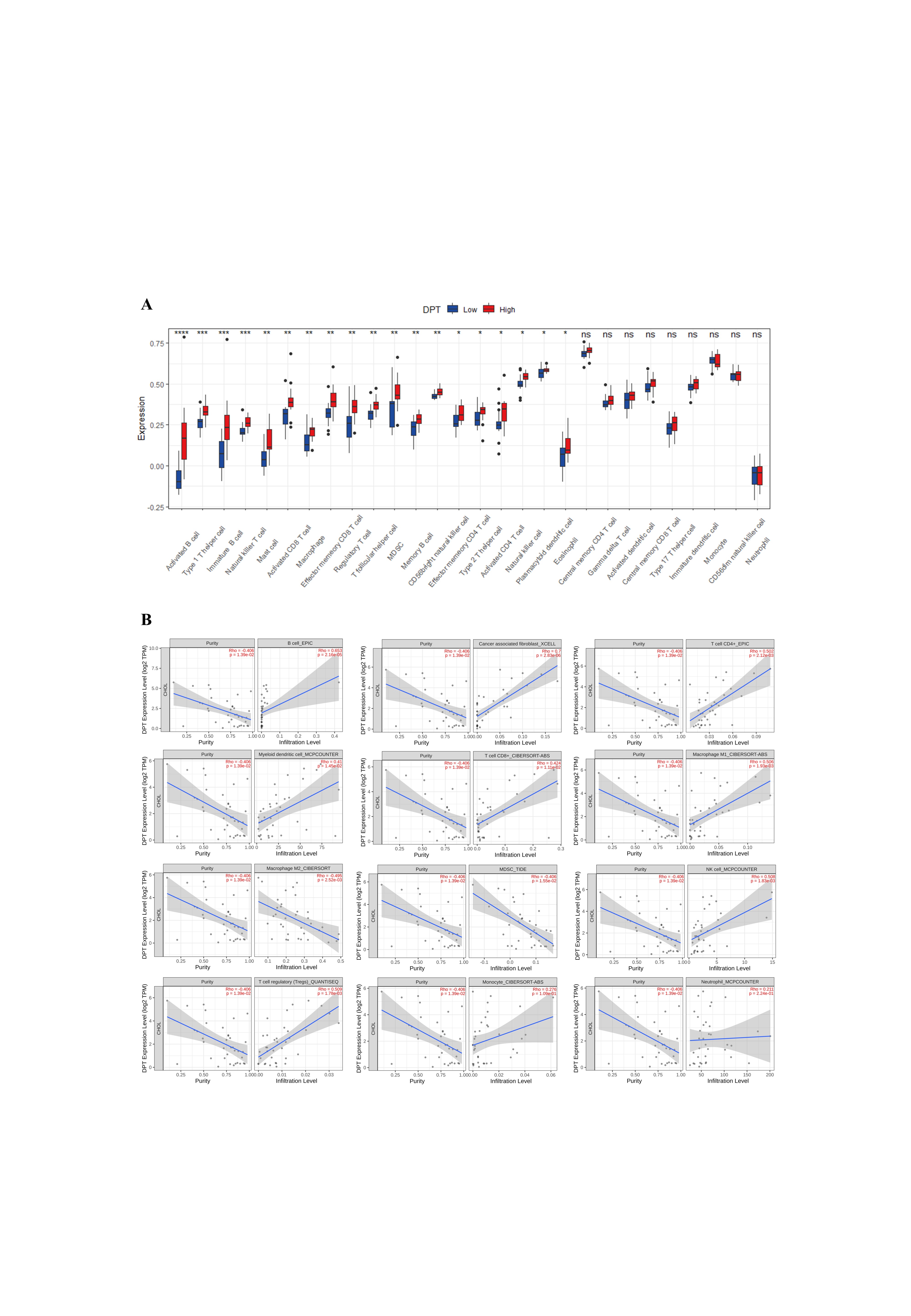

Supplement: Supplementary file 2 — Supplementary file2 (ZIP 4862 KB) [file 432_2023_5532_MOESM2_ESM.zip › SupplementaryFig. 3.jpg]

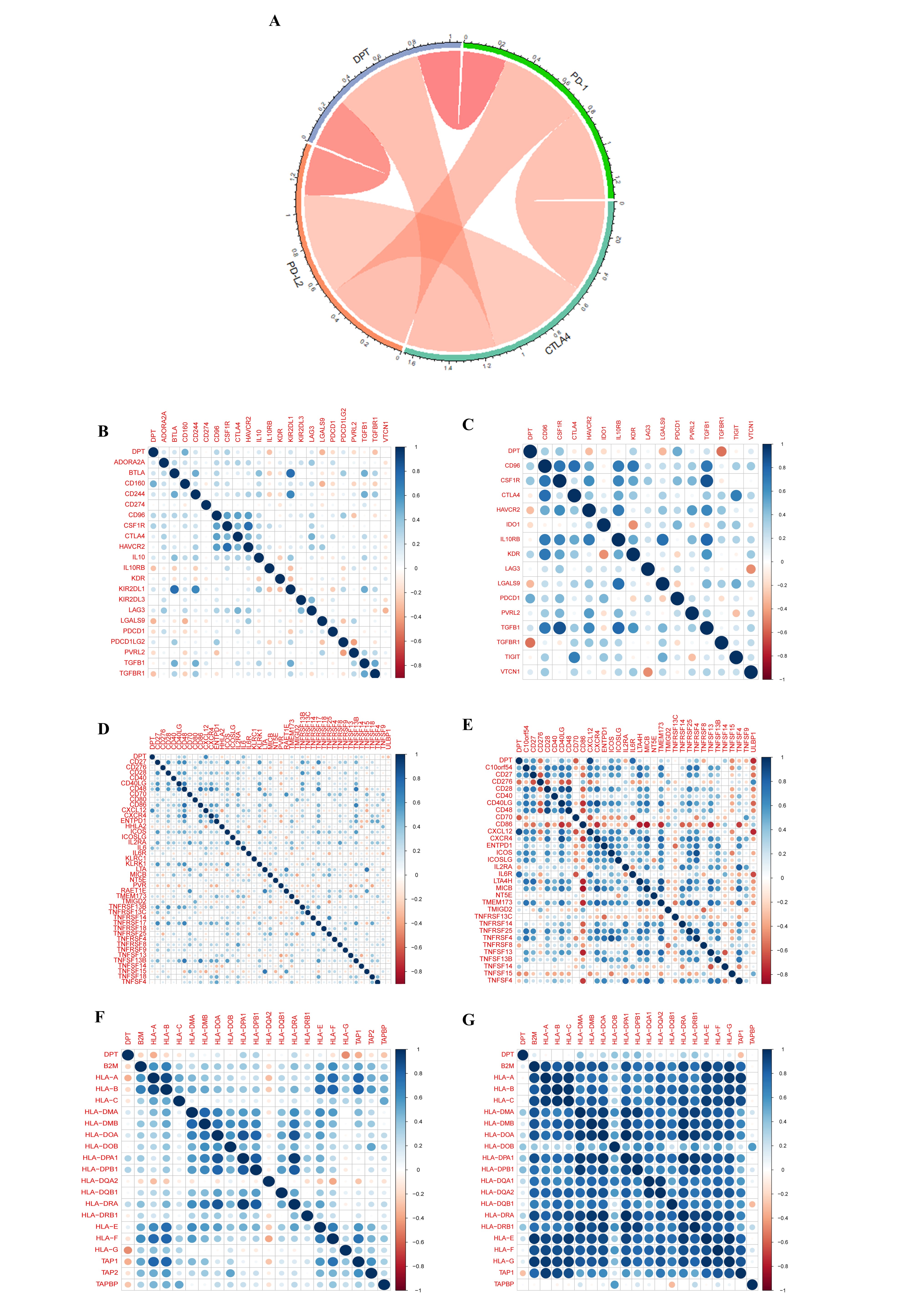

Supplement: Supplementary file 2 — Supplementary file2 (ZIP 4862 KB) [file 432_2023_5532_MOESM2_ESM.zip › SupplementaryFig. 4.jpg]

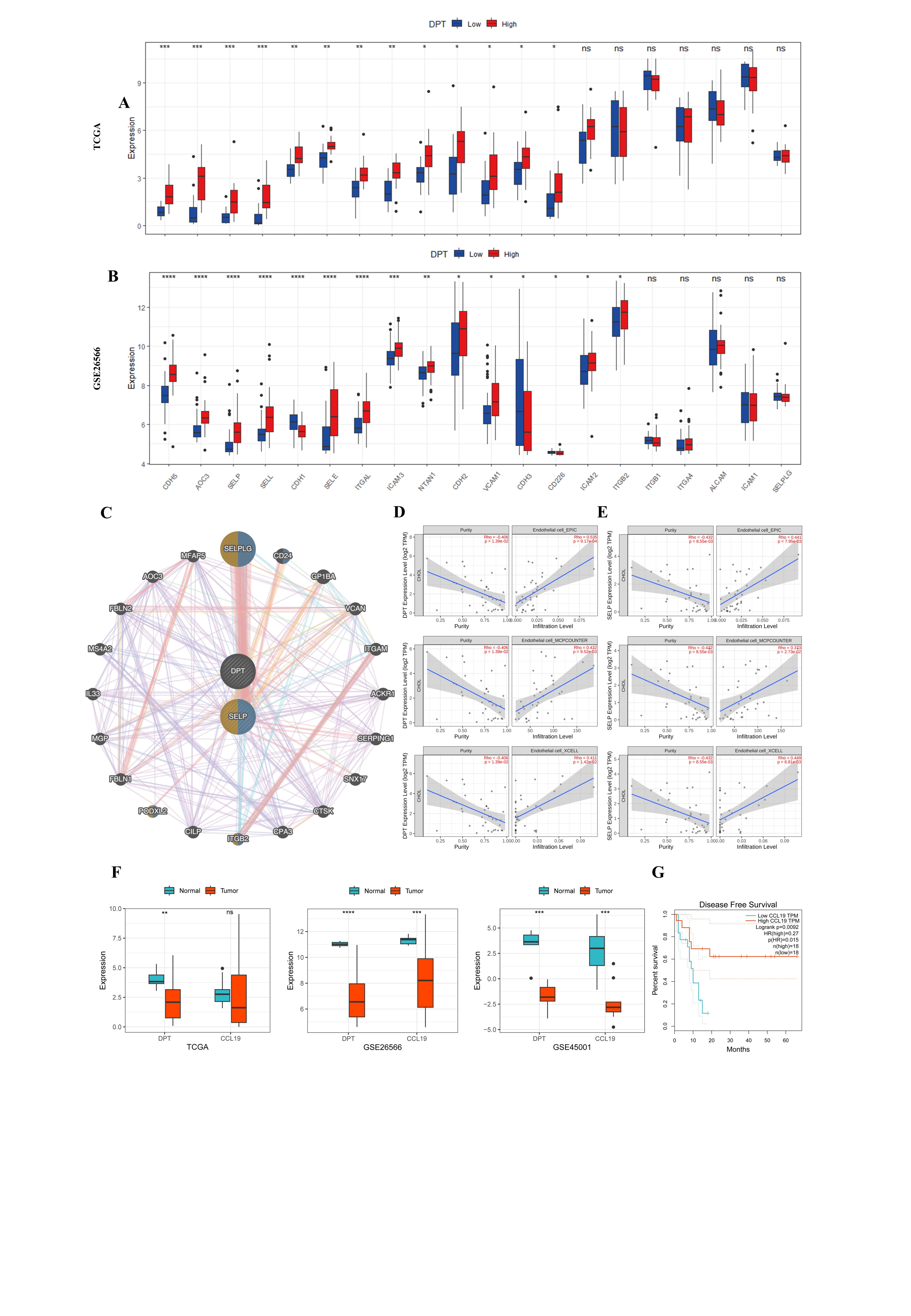

Supplement: Supplementary file 2 — Supplementary file2 (ZIP 4862 KB) [file 432_2023_5532_MOESM2_ESM.zip › SupplementaryFig. 5.jpg]

**Figure 9B**

**Figure 9E**

**Figure 9G**

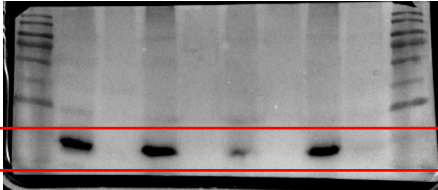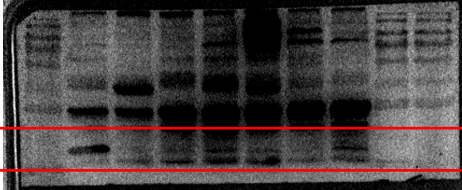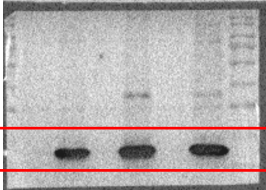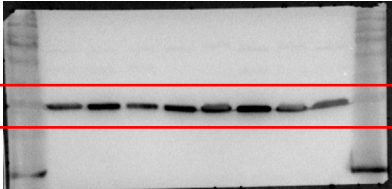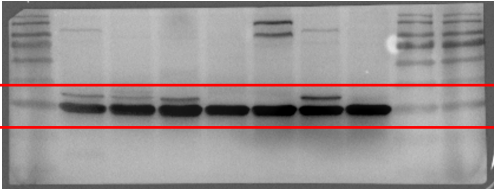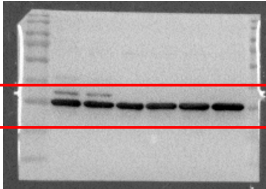

DPT: 22KD

GAPDH: 37KD

Supplement: Supplementary file 3 — Supplementary file3 (PDF 1117 KB) [file 432_2023_5532_MOESM3_ESM.pdf]
