# Supplementary material for: Downregulation of dermatopontin in cholangiocarcinoma cells suppresses CCL19 secretion of macrophages and immune infiltration
Source: J Cancer Res Clin Oncol. 2024 Feb 1;150(2):66. doi: 10.1007/s00432-023-05532-1 (PMC10834663; doi:10.1007/s00432-023-05532-1)
Supplement: Supplementary file 2 — Supplementary file2 (ZIP 4862 KB) [file 432_2023_5532_MOESM2_ESM.zip › Fugure Legend (revised).docx]

## Figure Legend

**Supplementary Fig. 1** Datasets from GEO and TCGA databases were dimensionality reduced. **(A-C)** Principal Component Analysis (PCA) of datasets from GEO and TCGA databases. (D) Intersection of TCGA-DEGs obtained by the three algorithms.

**Supplementary Fig. 2** The infiltration levels of immune cells in the low-risk group were significantly higher. **(A)** Using the ‘survminer’ package, calculate the riskscore cut point. **(B)** Investigation of the relationship between scores (stromal score, immune score, and estimate score) and model risk. **(C)** Immune infiltration level in high- and low-risk groups.

**Supplementary Fig. 3** Most types of immune cells exhibited a strong positive correlation with DPT expression. **(A-B)** Evaluation of the correlation between DPT and immune infiltration with TCGA and Timer 2.0 database.

**Supplementary Fig. 4 (A)** A chord diagram was used to assess the association between DPT and immune checkpoint molecules such as PD-1, PD-L2, and CTLA4. **(B-C)** Recognition of the correlation between DPT and immunoinhibitors in GSE26566 and GSE45001 datasets. **(D-E)** Detection of the correlation between DPT and immunostimulators in GSE26566 and GSE45001 datasets. **(F-G)** Identification of the correlation between DPT and MHCs in GSE26566 and GSE45001 datasets.

**Supplementary Fig. 5** DPT may target SELP to promote immune cell infiltration in CHOL. **(A-B)** The expression levels of adhesion molecules were detected between high- and low-DPT groups in the TCGA and GSE26566 CHOL datasets. **(C)** Analysis of the correlation between DPT and adhesion molecule SELP using GeneMANIA website. **(D-E)** Expression of DPT and CCL19 were positively correlated with endothelial with 3 algorithms (EPIC, MCPCOUNTER, and XCELL). Blue and brown represent adhesion molecules. **(F)** The expression of CCL19 in GSE26566, GSE45001, and TCGA-CHOL datasets. **(G)** Evaluation of the relationship between CCL19 and prognosis of CHOL patients using the GEPIA website.
