# Supplementary material for: Downregulation of dermatopontin in cholangiocarcinoma cells suppresses CCL19 secretion of macrophages and immune infiltration
Source: J Cancer Res Clin Oncol. 2024 Feb 1;150(2):66. doi: 10.1007/s00432-023-05532-1 (PMC10834663; doi:10.1007/s00432-023-05532-1)
Supplement: Supplementary file 1 — Supplementary file1 (ZIP 153266 KB) [file 432_2023_5532_MOESM1_ESM.zip › Websites/GeneMANIA/DPT CCL19.pdf]

GeneMANIA report

Created on : 6 June 2023 19:28:21
Last database update : 13 August 2021 00:00:00
Application version : 3.6.0

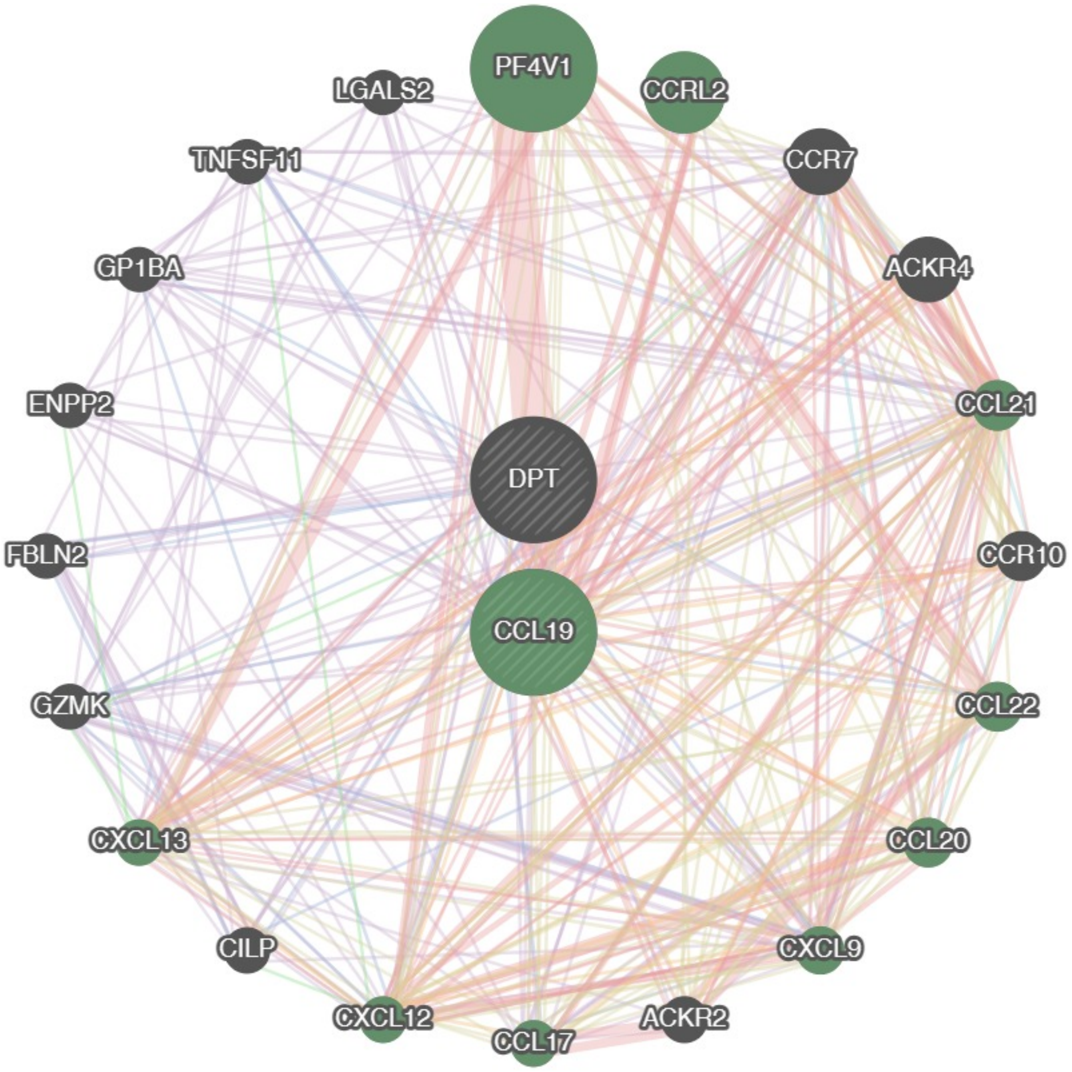

Networks

- Physical Interactions
- Co-expression
- Predicted
- Co-localization
- Genetic Interactions
- Pathway
- Shared protein domains

Functions

- chemokine receptor binding

Search parameters

Organism Homo sapiens (human)

Genes DPT , CCL19

Network weighting Automatically selected weighting method

Networks

A

Abbasi-Schild-Poulter-2019 , Abu-Odeh-Aqeilan-2014 , Achuthankutty-Mailand-2019 , Agrawal-Sedivy-2010 , Ahn-Lee-2008 , Albers-Koegl-2005 , Alexander-Wang-2018 , Alexandru-Deshaies-2008 , Alizadeh-Staudt-2000 , Alsulami-Cagney-2019 , An-Sun-2017 , Andresen-Flores-Morales-2014 , Arbogast-Gros-2019 , Arijs-Rutgeerts-2009 , Arroyo-Aloy-2014 , Arroyo-Aloy-2015 , Asadi-Dhanvantari-2018

B

Bailey-Hieter-2015 , Bandyopadhyay-Ideker-2010 , Banks-Washburn-2016 , Bantscheff-Drewes-2011 , Barr-Knapp-2009 , Barreiro-Alonso-Cerdán-2018 , Barrios-Rodiles-Wrana-2005 , Behrends-Harper-2010 , Behzadnia-Lührmann-2007 , Benleulmi-Chaachoua-Jockers-2016 A , Benleulmi-Chaachoua-Jockers-2016 B , Bennett-Harper-2010 , Benzinger-Hermeking-2005 , Berggård-James-2006 , Bett-Hay-2013 , Beyer-Boldt-2018 , Bhatnagar-Attie-2014 , Bild-Nevins-2006 B , BIOGRID-SMALL-SCALE-STUDIES , BIOGRID-SMALL-SCALE-STUDIES , Bishof-Seyfried-2018 , Blandin-Richard-2013 , Blomen-Brummelkamp-2015 , Blomen-Brummelkamp-2015 , Bogachek-Weigel-2014 , Boldrick-Relman-2002 , Boldt-Roepman-2016 , Botham-Schimmer-2019 , Bouwmeester-Superti-Furga-2004 , Brady-Omary-2018 , Brajenovic-Drewes-2004 , Brehme-Superti-Furga-2009 , Burington-Shaughnessy-2008 , Butland-Hayden-2014 , Byron-Humphries-2012

C

Cai-Conaway-2007 , Camargo-Brandon-2007 , Campos-Reinberg-2015 , Cao-Chinnaiyan-2014 , Carmon-Liu-2014 , Caron-van Attikum-2019 , CELL_MAP , Chen-Brown-2002 , Chen-Ge-2013 A , Chen-Ge-2013 B , Chen-Guan-2018 , Chen-Huang-2014 , Chen-Krogan-2018 , Chen-Yu-2018 , Chen-Zhang-2013 , Chen-Zhou-2019 , Cheng-DeCaprio-2017 , Chi-Reed-2018 , Chitale-Richly-2017 , Choi-Beutler-2019 , Choi-Busino-2018 , Choudhury-Michlewski-2017 , Christianson-Kopito-2011 , Cloutier-Coulombe-2013 , Cloutier-Coulombe-2017 , Colicelli-2010 , Colland-Gauthier-2004 , Conte-Perez-Oliva-2018 , Cooper-Green-2015 , Corominas-Iakoucheva-2014 , Couzens-Gingras-2013 , Cox-Rizzino-2013 , Coyaud-Raught-2015 , Crow-Cristea-2017

D

Daakour-Twizere-2016 , Dabbaghizadeh-Tanguay-2018 , Dart-Wells-2015 , Das-Broemer-2019 , Davis-Glaunsinger-2015 , de Hoog-Mann-2004 , Devarajan-Ketha-Kumar-2012 , Diner-Cristea-2015 , Dittmer-Misteli-2014 , Dobbin-Giordano-2005 ,

D

Douanne-Bidère-2019 , Drissi-Boisvert-2015 , Du-Krogan-2017

E

Elliott-Gyrd-Hansen-2016 , Emdal-Olsen-2015 , Enzo-Dupont-2015 , Ertych-Bastians-2016 , Ewing-Figeys-2007

F

Fang-Lin-2011 , Faust-Frankel-2018 , Fenner-Prehn-2010 , Floyd-Pagliarini-2016 , Foerster-Ritter-2013 , Fogeron-Lange-2013 , Fonseca-Damgaard-2015 , Foster-Marshall-2013 , Fragoza-Yu-2019 , Freibaum-Taylor-2010

G

Gabriel-Baumgrass-2016 , Gallardo-Vara-Bernabeu-2019 , Galligan-Howley-2015 , Gao-Reinberg-2012 , Gao-Vaziri-2016 , Garzia-Sonenberg-2017 , Gautier-Hall-2009 , Giannone-Liu-2010 , Gilmore-Washburn-2016 , Giurato-Tarallo-2018 , Glatter-Gstaiger-2009 , Gloeckner-Ueffing-2007 , Goehler-Wanker-2004 , Gordon-Krogan-2020 , Goudreault-Gingras-2009 , Greco-Cristea-2011 , Grossmann-Stelzl-2015 , Guarani-Harper-2014 , Guard-Old-2019 , Guardia-Laguarta-Przedborski-2019 , Guderian-Grimmler-2011 , Gupta-Pelletier-2015

H

Han-Bassik-2017 A , Han-Bassik-2017 B , Hanson-Clayton-2014 , Hauri-Beisel-2016 , Hauri-Gstaiger-2013 , Havrylov-Redowicz-2009 , Havugimana-Emili-2012 , Hayes-Urbé-2012 , Hegele-Stelzl-2012 A , Hegele-Stelzl-2012 B , Heidelberger-Beli-2018 , Hein-Mann-2015 , Hermjakob-Apweiler-2004 , Herr-Helleday-2015 , Hoffmeister-Längst-2017 , Horlbeck-Gilbert-2018 A , Horlbeck-Gilbert-2018 B , Hosp-Selbach-2015 , Hou-Chen-2018 , Hou-Huang-2017 , Hu-Woods-2019 , Hu-Yin-2019 , Hubel-Pichlmair-2019 , Huber-Hoelz-2017 , HUMANCYC , Humphries-Humphries-2009 , Hussain-Aldaz-2018 , Hutchins-Peters-2010 , Huttlin-Gygi-2015 , Huttlin-Harper-2017 , Hüttenhain-Krogan-2019

I

I2D-BIND-Fly2Human , I2D-BIND-Mouse2Human , I2D-BIND-Rat2Human , I2D-BIND-Worm2Human , I2D-BIND-Yeast2Human , I2D-BioGRID-Fly2Human , I2D-BioGRID-Mouse2Human , I2D-BioGRID-Rat2Human , I2D-BioGRID-Worm2Human , I2D-BioGRID-Yeast2Human , I2D-Chen-Pawson-2009-PiwiScreen-Mouse2Human , I2D-Formstecher-Daviet-2005-Embryo-Fly2Human , I2D-Formstecher-Daviet-2005-Head-Fly2Human , I2D-Giot-Rothbert-2003-High-Fly2Human , I2D-Giot-Rothbert-2003-Low-Fly2Human , I2D-INNATEDB-Mouse2Human , I2D-IntAct-Fly2Human , I2D-IntAct-Mouse2Human , I2D-IntAct-Rat2Human , I2D-IntAct-Worm2Human , I2D-IntAct-Yeast2Human , I2D-Krogan-Greenblatt-2006-Core-Yeast2Human , I2D-Krogan-Greenblatt-2006-NonCore-Yeast2Human , I2D-Li-Vidal-2004-CE-DATA-Worm2Human , I2D-Li-Vidal-2004-CORE-1-Worm2Human , I2D-Li-Vidal-2004-CORE-2-Worm2Human , I2D-Li-Vidal-2004-interolog-Worm2Human , I2D-Li-Vidal-2004-literature-Worm2Human , I2D-Li-

I

Vidal-2004-non-core-Worm2Human , I2D-Manual-Mouse2Human , I2D-Manual-Rat2Human , I2D-MGI-Mouse2Human , I2D-MINT-Fly2Human , I2D-MINT-Mouse2Human , I2D-MINT-Rat2Human , I2D-MINT-Worm2Human , I2D-MINT-Yeast2Human , I2D-MIPS-Yeast2Human , I2D-Ptacek-Snyder-2005-Yeast2Human , I2D-Stanyon-Finley-2004-CellCycle-Fly2Human , I2D-Tarassov-PCA-Yeast2Human , I2D-Tewari-Vidal-2004-TGFb-Worm2Human , I2D-vonMering-Bork-2002-High-Yeast2Human , I2D-vonMering-Bork-2002-Low-Yeast2Human , I2D-vonMering-Bork-2002-Medium-Yeast2Human , I2D-Wang-Orkin-2006-EScmplx-Mouse2Human , I2D-Wang-Orkin-2006-EScmplxIP-Mouse2Human , I2D-Wang-Orkin-2006-EScmplxlow-Mouse2Human , I2D-Yu-Vidal-2008-GoldStd-Yeast2Human , IMID , Ingham-Pawson-2005 , Innocenti-Brown-2011 , INTERPRO , Iradi-Borchelt-2018 , IREF-bhf-ucl , IREF-bind , IREF-bind-translation , IREF-biogrid , IREF-corum , IREF-dip , IREF-hpidb , IREF-hprd , IREF-huri , IREF-innatedb , IREF-intact , IREF-intcomplex , IREF-matrixdb , IREF-mbinfo , IREF-mint , IREF-mppi , IREF-quickgo , IREF-reactome , IREF-SMALL-SCALE-STUDIES , IREF-SMALL-SCALE-STUDIES , IREF-spike , IREF-uniprotpp , IREF-virushost , Ivanochko-Arrowsmith-2019

J

Jain-Parker-2016 , Jang-Trono-2018 , Jeronimo-Coulombe-2007 , Jiang-de Kok-2017 , Jin-Pawson-2004 , Jirawatnotai-Sicinski-2011 , Johnson-Kerner-Wichterle-2015 , Johnson-Shoemaker-2003 , Jones-MacBeath-2006 , Joshi-Cristea-2013 , Jozwik-Carroll-2016 , Jäger-Krogan-2011

K

Kahle-Zoghbi-2011 , Kaltenbach-Hughes-2007 , Kang-Shin-2015 , Karras-Soengas-2019 , Kato-Sternberg-2014 , Katsogiannou-Rocchi-2014 , Kawahara-Paes Leme-2017 , Keller-Lee-2014 , Kennedy-Kolch-2020 A , Kennedy-Kolch-2020 B , Khanna-Parnaik-2018 , Kim-Major-2015 , Kneissl-Grummt-2003 , Koch-Hermeking-2007 , Kotlyar-Jurisica-2015 , Kristensen-Foster-2012 , Kumar-Maddika-2017 , Kumar-Vertegaal-2017 , Kupka-Walczak-2016 , Kärblane-Sarmiento-2015 , Kırlı-Görlich-2015

L

Lambert-Gingras-2015 , Lampert-Peter-2018 , Lau-Ronai-2012 , Lee-Choi-2016 , Lee-Choi-2017 , Lee-Jeong-2017 , Lee-Jou-2019 , Lee-Mayr-2019 , Lee-Songyang-2011 , Lehner-Sanderson-2004 A , Lehner-Sanderson-2004 B , Leung-Jones-2014 , Leung-Miller-2017 , Li-Chen-2015 , Li-Dorf-2011 A , Li-Dorf-2011 B , Li-Dorf-2014 , Li-Fu-2017 , Li-Haura-2013 , Li-Hung-2019 , Li-Lu-2018 , Li-Wang-2016 , Li-Zhou-2017 , Liebelt-Vertegaal-2020 , Lim-Zoghbi-2006 , Lin-Smith-2010 , Lipp-Guthrie-2015 , Liu-Chen-2019 , Liu-Sun-2019 , Liu-Takahashi-2017 , Liu-Tan-2018 , Liu-Varjosalo-2018 , Liu-Wang-2012 , Liu-Xu-2018 , Liu-Yang-2019 , Llères-Lamond-2010 , Loch-Strickler-2012 , Low-Heck-2014 , Lu-Bohr-2017 , Lu-Zhang-2013 , Luck-Calderwood-2020 , Lum-Cristea-2018 , Luo-Elledge-2009

M

Mak-Moffat-2010 , Malinová-Verheggen-2017 , Mallon-McKay-2013 , Malovannaya-Qin-2010 , Malty-Babu-2017 , Markson-Sanderson-2009 , Martin-Elledge-2017 , Maréchal-Zou-2014 , Matsumoto-Nakayama-2005 , Matsuoka-Elledge-2007 , McCracken-Blencowe-2005 , McFarland-Nussbaum-2008 , McNamara-D'Orso-2016 , Meek-Piwnica-Worms-2004 , Menon-Litovchick-2019 , Milev-Mouland-2012 , Miyamoto-Sato-Yanagawa-2010 , Mohammed-Carroll-2013 , Moon-Kim-2014 , Moutaoufik-Babu-2019 , Mugabo-Lim-2018 , Muller-Demeret-2012 , Murakawa-Landthaler-2015

N

Nakamura-Groth-2019 , Nakayama-Ohara-2002 , Napolitano-Meroni-2011 , Narayan-Bennett-2012 , Nassa-Weisz-2019 , Nathan-Goldberg-2013 , NCI_NATURE , Neganova-Lako-2011 , Newman-Keating-2003 , Noguchi-Kawahara-2018 , Nowak-Sommer-2019

O

Oliviero-Cagney-2015 , Oliviero-Cagney-2016 , Olma-Pintard-2009 , Oláh-Ovádi-2011 , Ouyang-Gill-2009

P

Panigrahi-Pati-2012 , Pankow-Yates-2015 , Pao-Virdee-2018 , Papp-Lamia-2015 , Pech-Settleman-2019 , Perez-Hernandez-Yáñez-Mó-2013 , Perez-Perri-Espinosa-2016 , Perou-Botstein-1999 , Perou-Botstein-2000 , Persaud-Rotin-2009 A , Persaud-Rotin-2009 B , Petschnigg-Stagljar-2014 , PFAM , Phillips-Corn-2013 , Pichlmair-Superti-Furga-2011 , Pichlmair-Superti-Furga-2012 , Pilling-Cooper-2017 , Pladevall-Morera-Lopez-Contreras-2019 , Ptushkina-Ray-2017

R

Raisner-Gascoigne-2018 , Ramachandran-LaBaer-2004 , Raman-Harper-2015 , Ramaswamy-Golub-2001 , Ravasi-Hayashizaki-2010 , REACTOME , Reinke-Keating-2010 , Reinke-Keating-2013 , Rengasamy-Walsh-2017 , Reyniers-Taymans-2014 , Richter-Chrzanowska-Lightowlers-2010 , Rieger-Chu-2004 , Rivera-Paes Leme-2018 , Rodriguez-von Kriegsheim-2016 , Roewenstrunk-de la Luna-2019 , Rolland-Vidal-2014 , Rosenbluh-Hahn-2016 , Rosenwald-Staudt-2001 , Ross-Perou-2001 , Roth-Zlotnik-2006 , Rowbotham-Mermoud-2011 , Roy-Pardo-2014 , Roy-Parent-2013 , Rual-Vidal-2005

S

Saez-Vilchez-2018 , Sahni-Vidal-2015 , Saito-Kobarg-2017 , Sala-Ampe-2017 , Salvetti-Greco-2016 , Sang-Jackson-2011 , Sato-Conaway-2004 , Savidis-Brass-2016 , Schadt-Shoemaker-2004 , Schiza-Diamandis-2018 , Scholz-Taylor-2016 , Scifo-Lalowski-2015 , Scott-Guy-2017 , Scott-Schulman-2016 , Shami Shah-Baskin-2019 , Shen-Chen-2019 , Shen-Mali-2017 , Sherman-Teitell-2010 , Simabuco-Zanchin-2019 , Singh-Moore-2012 , So-Colwill-2015 , Sokolina-Stagljar-2017 , Soler-López-Aloy-2011 , Sowa-Harper-2009 , Srivas-Ideker-2016 , St-Denis-Gingras-2015 , St-Denis-

S

Gingras-2016 , Stehling-Lill-2012 , Stehling-Lill-2013 , Stelzl-Wanker-2005 , Stuart-Kim-2003 , Sundell-Ivarsson-2018 , Suter-Wanker-2013 , Swayampakula-Dedhar-2017

T

Taipale-Lindquist-2012 , Taipale-Lindquist-2014 , Takahashi-Conaway-2011 , Tang-Wang-2019 , Tarallo-Weisz-2011 , Teixeira-Gomes-2010 , Teixeira-Laman-2016 A , Teixeira-Laman-2016 B , Thalappilly-Dusetti-2008 , Thompson-Luchansky-2014 , Tiemann-Kani-2019 , Tomkins-Manzoni-2018 , Tong-Moran-2014 , Toyoshima-Grandori-2012 , Trepte-Wanker-2018 A , Trepte-Wanker-2018 B , Tsai-Cristea-2012

U

Ugidos-Vandenbroeck-2019

V

Van Acker-Dewilde-2019 , Van Alstyne-Pellizzoni-2018 , Van Quickelberghe-Gevaert-2018 , van Wijk-Timmers-2009 , Vandamme-Angrand-2011 , Varier-Vermeulen-2016 , Varjosalo-Gstaiger-2013 A , Varjosalo-Gstaiger-2013 B , Varjosalo-Superti-Furga-2013 , Vastrik-Stein-2007 , Venkatesan-Vidal-2009 , Viita-Vartiainen-2019 , Vinayagam-Wanker-2011 , Virok-Fülöp-2011 , Vizeacoumar-Moffat-2013 , von Hundelshausen-Weber-2017

W

Wallach-Kramer-2013 , Wan-Emili-2015 , Wang-Balch-2006 , Wang-Cheung-2015 , Wang-He-2008 , Wang-Huang-2017 , Wang-Liu-2019 , Wang-Maris-2006 , Wang-Xiong-2019 , Wang-Xu-2015 , Wang-Yang-2011 , Watanabe-Fujita-2018 , Weimann-Stelzl-2013 A , Weimann-Stelzl-2013 B , Weinmann-Meister-2009 , Weishäupl-Schmidt-2019 , Weith-Meyer-2018 , Whisenant-Salomon-2015 , Wilkinson-Coba-2019 , Willingham-Muchowski-2003 , Winczura-Jensen-2018 , Wong-O'Bryan-2012 , Woods-Monteiro-2012 A , Woods-Monteiro-2012 B , Woodsmith-Sanderson-2012 , Wu-Garvey-2007 , Wu-Li-2007 , Wu-Ma-2012 , Wu-Stein-2010 , Wu-Stein-2010

X

Xiao-Brown-2018 , Xiao-Lefkowitz-2007 , Xie-Cong-2013 , Xie-Green-2012 , Xie-Zhang-2017 , Xu-Ye-2012 , Xu-Zetter-2016

Y

Yachie-Roth-2016 , Yadav-Varjosalo-2017 , Yamauchi-Maeda-2018 , Yang-Brasier-2015 , Yang-Chen-2010 , Yang-Maurer-2018 , Yang-Vidal-2016 , Yang-Wang-2018 , Yao-Stagljar-2017 A , Yao-Stagljar-2017 B , Yatim-Benkirane-2012 , Yeung-Dougan-2019 , Yu-Chow-2013 , Yu-Engel-2018 , Yu-Vidal-2011 , Yue-Liu-2018

Z

Zanon-Pichler-2013 , Zeller-Wei-2006 , Zhang-Shang-2006 , Zhang-Vermeulen-2017 , Zhang-Wang-2018 , Zhang-Wheeler-2014 , Zhang-Xu-2018 , Zhang-Zou-2011 , Zhao-Krug-2005 , Zhao-Yang-2011 , Zhong-Vidal-2016 , Zhou-Conrads-2004 , Zhou-

Z

Hanemann-2016 , Zhu-Liu-2018

Genes

Gene	Description	Rank
DPT	dermatopontin [Source:HGNC Symbol;Acc:HGNC:3011]	N/A
CCL19	C-C motif chemokine ligand 19 [Source:HGNC Symbol;Acc:HGNC:10617]	N/A
PF4V1	platelet factor 4 variant 1 [Source:HGNC Symbol;Acc:HGNC:8862]	1
CCRL2	C-C motif chemokine receptor like 2 [Source:HGNC Symbol;Acc:HGNC:1612]	2
CCR7	C-C motif chemokine receptor 7 [Source:HGNC Symbol;Acc:HGNC:1608]	3
ACKR4	atypical chemokine receptor 4 [Source:HGNC Symbol;Acc:HGNC:1611]	4
CCL21	C-C motif chemokine ligand 21 [Source:HGNC Symbol;Acc:HGNC:10620]	5
CCR10	C-C motif chemokine receptor 10 [Source:HGNC Symbol;Acc:HGNC:4474]	6
CCL22	C-C motif chemokine ligand 22 [Source:HGNC Symbol;Acc:HGNC:10621]	7
CCL20	C-C motif chemokine ligand 20 [Source:HGNC Symbol;Acc:HGNC:10619]	8
CXCL9	C-X-C motif chemokine ligand 9 [Source:HGNC Symbol;Acc:HGNC:7098]	9
ACKR2	atypical chemokine receptor 2 [Source:HGNC Symbol;Acc:HGNC:1565]	10
CCL17	C-C motif chemokine ligand 17 [Source:HGNC Symbol;Acc:HGNC:10615]	11
CXCL12	C-X-C motif chemokine ligand 12 [Source:HGNC Symbol;Acc:HGNC:10672]	12
CILP	cartilage intermediate layer protein [Source:HGNC Symbol;Acc:HGNC:1980]	13
CXCL13	C-X-C motif chemokine ligand 13 [Source:HGNC Symbol;Acc:HGNC:10639]	14
GZMK	granzyme K [Source:HGNC Symbol;Acc:HGNC:4711]	15
FBLN2	fibulin 2 [Source:HGNC Symbol;Acc:HGNC:3601]	16
ENPP2	ectonucleotide pyrophosphatase/phosphodiesterase 2 [Source:HGNC Symbol;Acc:HGNC:3357]	17
GP1BA	glycoprotein Ib platelet subunit alpha [Source:HGNC Symbol;Acc:HGNC:4439]	18
TNFSF11	TNF superfamily member 11 [Source:HGNC Symbol;Acc:HGNC:11926]	19
LGALS2	galectin 2 [Source:HGNC Symbol;Acc:HGNC:6562]	20

Networks

Physical Interactions 77.64%

Wang-Huang-2017 4.41%

Molecular Details Underlying Dynamic Structures and Regulation of the Human 26S Proteasome. Wang et al (2017). *Mol Cell Proteomics*

Physical Interactions with 73 interactions from BioGRID

von Hundelshausen-Weber-2017 2.33%

Chemokine interactome mapping enables tailored intervention in acute and chronic inflammation. von Hundelshausen et al (2017). *Sci Transl Med*

Physical Interactions with 213 interactions from iRefIndex

McFarland-Nussbaum-2008 2.21%

Proteomics analysis identifies phosphorylation-dependent alpha-synuclein protein interactions. McFarland et al (2008). *Mol Cell Proteomics*

Physical Interactions with 159 interactions from BioGRID

Guardia-Laguarta-Przedborski-2019 1.66%

PINK1 Content in Mitochondria is Regulated by ER-Associated Degradation. Guardia-Laguarta et al (2019). *J Neurosci*

Physical Interactions with 468 interactions from BioGRID

Maréchal-Zou-2014 1.46%

PRP19 transforms into a sensor of RPA-ssDNA after DNA damage and drives ATR activation via a ubiquitin-mediated circuitry. Maréchal et al (2014). *Mol Cell*

Physical Interactions with 996 interactions from BioGRID

Singh-Moore-2012 1.40%

The cellular EJC interactome reveals higher-order mRNP structure and an EJC-SR protein nexus. Singh et al (2012). *Cell*

Physical Interactions with 235 interactions from iRefIndex

Whisenant-Salomon-2015 1.39%

The Activation-Induced Assembly of an RNA/Protein Interactome Centered on the Splicing Factor U2AF2 Regulates Gene Expression in Human CD4 T Cells. Whisenant et al (2015). *PLoS One*

Physical Interactions with 237 interactions from BioGRID

Tsai-Cristea-2012 1.30%

Functional proteomics establishes the interaction of SIRT7 with chromatin remodeling complexes and expands its role in regulation of RNA polymerase I transcription. Tsai et al (2012). *Mol Cell Proteomics*

Physical Interactions with 651 interactions from BioGRID

Kneissl-Grummt-2003 1.26%

Interaction and assembly of murine pre-replicative complex proteins in yeast and mouse cells. Kneissl et al (2003). *J Mol Biol*

Physical Interactions with 81 interactions from BioGRID

Zhang-Vermeulen-2017 1.20%

An Interaction Landscape of Ubiquitin Signaling. Zhang et al (2017). *Mol Cell*

Physical Interactions with 242 interactions from BioGRID

Narayan-Bennett-2012 1.16%

Short-chain 3-hydroxyacyl-coenzyme A dehydrogenase associates with a protein super-complex integrating multiple metabolic

Physical Interactions 77.64%

Narayan-Bennett-2012

pathways. Narayan et al (2012). *PLoS One*

Physical Interactions with 109 interactions from BioGRID

Li-Fu-2017 1.13%

The OncoPPi network of cancer-focused protein-protein interactions to inform biological insights and therapeutic strategies. Li et al (2017). *Nat Commun*

Physical Interactions with 749 interactions from BioGRID

Li-Hung-2019 1.09%

MET Inhibitors Promote Liver Tumor Evasion of the Immune Response by Stabilizing PDL1. Li et al (2019). *Gastroenterology*

Physical Interactions with 179 interactions from BioGRID

Richter-Chrzanowska-Lightowlers-2010 1.07%

A functional peptidyl-tRNA hydrolase, ICT1, has been recruited into the human mitochondrial ribosome. Richter et al (2010). *EMBO J*

Physical Interactions with 204 interactions from BioGRID

Ertych-Bastians-2016 1.07%

CHK2-BRCA1 tumor-suppressor axis restrains oncogenic Aurora-A kinase to ensure proper mitotic microtubule assembly. Ertych et al (2016). *Proc Natl Acad Sci U S A*

Physical Interactions with 486 interactions from BioGRID

Yu-Chow-2013 1.01%

VCP phosphorylation-dependent interaction partners prevent apoptosis in Helicobacter pylori-infected gastric epithelial cells. Yu et al (2013). *PLoS One*

Physical Interactions with 280 interactions from BioGRID

Lee-Jou-2019 0.98%

Parkinson's disease-associated LRRK2-G2019S mutant acts through regulation of SERCA activity to control ER stress in astrocytes. Lee et al (2019). *Acta Neuropathol Commun*

Physical Interactions with 218 interactions from BioGRID

Colicelli-2010 0.95%

ABL tyrosine kinases: evolution of function, regulation, and specificity. Colicelli (2010). *Sci Signal*

Physical Interactions with 147 interactions from iRefIndex

Matsumoto-Nakayama-2005 0.94%

Large-scale analysis of the human ubiquitin-related proteome. Matsumoto et al (2005). *Proteomics*

Physical Interactions with 311 interactions from BioGRID

Kotlyar-Jurisica-2015 0.94%

In silico prediction of physical protein interactions and characterization of interactome orphans. Kotlyar et al (2015). *Nat Methods*

Physical Interactions with 121 interactions from BioGRID

Petschnigg-Stagljar-2014 0.92%

The mammalian-membrane two-hybrid assay (MaMTH) for probing membrane-protein interactions in human cells. Petschnigg et al (2014). *Nat Methods*

Physical Interactions with 116 interactions from iRefIndex

Guarani-Harper-2014 0.89%

TIMMDC1/C3orf1 functions as a membrane-embedded mitochondrial complex I assembly factor through association with the

Physical Interactions 77.64%

Guarani-Harper-2014

MCIA complex. Guarani et al (2014). *Mol Cell Biol*

Physical Interactions with 323 interactions from BioGRID

Lu-Zhang-2013 0.81%

The HECT type ubiquitin ligase NEDL2 is degraded by anaphase-promoting complex/cyclosome (APC/C)-Cdh1, and its tight regulation maintains the metaphase to anaphase transition. Lu et al (2013). *J Biol Chem*

Physical Interactions with 186 interactions from iRefIndex

Berggård-James-2006 0.78%

140 mouse brain proteins identified by Ca2+-calmodulin affinity chromatography and tandem mass spectrometry. Berggård et al (2006). *J Proteome Res*

Physical Interactions with 151 interactions from BioGRID

Kupka-Walczak-2016 0.75%

SPATA2-Mediated Binding of CYLD to HOIP Enables CYLD Recruitment to Signaling Complexes. Kupka et al (2016). *Cell Rep*

Physical Interactions with 411 interactions from BioGRID

Freibaum-Taylor-2010 0.72%

Global analysis of TDP-43 interacting proteins reveals strong association with RNA splicing and translation machinery. Freibaum et al (2010). *J Proteome Res*

Physical Interactions with 160 interactions from iRefIndex

Perez-Perri-Espinosa-2016 0.65%

The TIP60 Complex Is a Conserved Coactivator of HIF1A. Perez-Perri et al (2016). *Cell Rep*

Physical Interactions with 118 interactions from BioGRID

Jirawatnotai-Sicinski-2011 0.65%

A function for cyclin D1 in DNA repair uncovered by protein interactome analyses in human cancers. Jirawatnotai et al (2011). *Nature*

Physical Interactions with 122 interactions from BioGRID

Kırlı-Görlich-2015 0.65%

A deep proteomics perspective on CRM1-mediated nuclear export and nucleocytoplasmic partitioning. Kırlı et al (2015). *Elife*

Physical Interactions with 1,034 interactions from BioGRID

Leung-Jones-2014 0.62%

Enhanced prediction of Src homology 2 (SH2) domain binding potentials using a fluorescence polarization-derived c-Met, c-Kit, ErbB, and androgen receptor interactome. Leung et al (2014). *Mol Cell Proteomics*

Physical Interactions with 199 interactions from iRefIndex

Yatim-Benkirane-2012 0.60%

NOTCH1 nuclear interactome reveals key regulators of its transcriptional activity and oncogenic function. Yatim et al (2012). *Mol Cell*

Physical Interactions with 127 interactions from BioGRID

Mak-Moffat-2010 0.60%

A lentiviral functional proteomics approach identifies chromatin remodeling complexes important for the induction of pluripotency. Mak et al (2010). *Mol Cell Proteomics*

Physical Interactions with 110 interactions from BioGRID

Chen-Ge-2013 B 0.60%

Physical Interactions 77.64%

Chen-Ge-2013 B

Bcl2-associated athanogene 3 interactome analysis reveals a new role in modulating proteasome activity. Chen et al (2013). *Mol Cell Proteomics*

Physical Interactions with 173 interactions from BioGRID

Zanon-Pichler-2013 0.59%

Profiling of Parkin-binding partners using tandem affinity purification. Zanon et al (2013). *PLoS One*

Physical Interactions with 195 interactions from BioGRID

Raisner-Gascoigne-2018 0.57%

Enhancer Activity Requires CBP/P300 Bromodomain-Dependent Histone H3K27 Acetylation. Raisner et al (2018). *Cell Rep*

Physical Interactions with 174 interactions from BioGRID

Jang-Trono-2018 0.57%

KAP1 facilitates reinstatement of heterochromatin after DNA replication. Jang et al (2018). *Nucleic Acids Res*

Physical Interactions with 699 interactions from BioGRID

Ahn-Lee-2008 0.56%

The tumour suppressor PTEN mediates a negative regulation of the E3 ubiquitin-protein ligase Nedd4. Ahn et al (2008). *Biochem J*

Physical Interactions with 105 interactions from BioGRID

IREF-reactome 0.55%

Physical Interactions with 111,926 interactions from iRefIndex

Vastrik-Stein-2007 0.55%

Reactome: a knowledge base of biologic pathways and processes. Vastrik et al (2007). *Genome Biol*

Physical Interactions with 111,926 interactions from iRefIndex

Liu-Varjosalo-2018 0.50%

An AP-MS- and BioID-compatible MAC-tag enables comprehensive mapping of protein interactions and subcellular localizations. Liu et al (2018). *Nat Commun*

Physical Interactions with 825 interactions from BioGRID

Wang-Liu-2019 0.48%

POH1 contributes to hyperactivation of TGF- signaling and facilitates hepatocellular carcinoma metastasis through deubiquitinating TGF- receptors and caveolin-1. Wang et al (2019). *EBioMedicine*

Physical Interactions with 180 interactions from iRefIndex

Murakawa-Landthaler-2015 0.47%

RC3H1 post-transcriptionally regulates A20 mRNA and modulates the activity of the IKK/NF- Bpathway. Murakawa et al (2015). *Nat Commun*

Physical Interactions with 155 interactions from BioGRID

Elliott-Gyrd-Hansen-2016 0.47%

SPATA2 Links CYLD to LUBAC, Activates CYLD, and Controls LUBAC Signaling. Elliott et al (2016). *Mol Cell*

Physical Interactions with 543 interactions from BioGRID

Lee-Choi-2016 0.46%

Interactomic analysis of REST/NRSF and implications of its functional links with the transcription suppressor TRIM28 during neuronal differentiation. Lee et al (2016). *Sci Rep*

Physical Interactions with 199 interactions from BioGRID

Physical Interactions 77.64%

Hüttenhain-Krogan-2019 0.46%

ARIH2 Is a Vif-Dependent Regulator of CUL5-Mediated APOBEC3G Degradation in HIV Infection. Hüttenhain et al (2019). *Cell Host Microbe*

Physical Interactions with 384 interactions from BioGRID

IREF-bhf-ucl 0.46%

Physical Interactions with 1,155 interactions from iRefIndex

Leung-Miller-2017 0.45%

ZMYM3 regulates BRCA1 localization at damaged chromatin to promote DNA repair. Leung et al (2017). *Genes Dev*

Physical Interactions with 247 interactions from BioGRID

Scifo-Lalowski-2015 0.45%

Quantitative analysis of PPT1 interactome in human neuroblastoma cells. Scifo et al (2015). *Data Brief*

Physical Interactions with 199 interactions from BioGRID

Koch-Hermeking-2007 0.45%

Large-scale identification of c-MYC-associated proteins using a combined TAP/MudPIT approach. Koch et al (2007). *Cell Cycle*

Physical Interactions with 207 interactions from BioGRID

Chen-Zhang-2013 0.43%

Quantitative study of the interactome of PKC involved in the EGF-induced tumor cell chemotaxis. Chen et al (2013). *J Proteome Res*

Physical Interactions with 182 interactions from BioGRID

Neganova-Lako-2011 0.42%

An important role for CDK2 in G1 to S checkpoint activation and DNA damage response in human embryonic stem cells. Neganova et al (2011). *Stem Cells*

Physical Interactions with 307 interactions from iRefIndex

Taipale-Lindquist-2012 0.42%

Quantitative analysis of HSP90-client interactions reveals principles of substrate recognition. Taipale et al (2012). *Cell*

Physical Interactions with 390 interactions from iRefIndex

Heidelberger-Beli-2018 0.41%

Proteomic profiling of VCP substrates links VCP to K6-linked ubiquitylation and c-Myc function. Heidelberger et al (2018). *EMBO Rep*

Physical Interactions with 1,347 interactions from BioGRID

Persaud-Rotin-2009 A 0.39%

Comparison of substrate specificity of the ubiquitin ligases Nedd4 and Nedd4-2 using proteome arrays. Persaud et al (2009). *Mol Syst Biol*

Physical Interactions with 139 interactions from BioGRID

McNamara-D'Orso-2016 0.38%

KAP1 Recruitment of the 7SK snRNP Complex to Promoters Enables Transcription Elongation by RNA Polymerase II. McNamara et al (2016). *Mol Cell*

Physical Interactions with 276 interactions from BioGRID

Xie-Cong-2013 0.38%

Deubiquitinase FAM/USP9X interacts with the E3 ubiquitin ligase SMURF1 protein and protects it from ligase activity-dependent self-degradation. Xie et al (2013). *J Biol Chem*

Physical Interactions 77.64%

Xie-Cong-2013

Physical Interactions with 170 interactions from BioGRID

Zhou-Conrads-2004 0.38%

"An investigation into the human serum ""interactome""." Zhou et al (2004). *Electrophoresis*

Physical Interactions with 112 interactions from iRefIndex

Alsulami-Cagney-2019 0.34%

SETD1A Methyltransferase Is Physically and Functionally Linked to the DNA Damage Repair Protein RAD18. Alsulami et al (2019). *Mol Cell Proteomics*

Physical Interactions with 288 interactions from BioGRID

Beyer-Boldt-2018 0.34%

CRISPR/Cas9-mediated Genomic Editing of Cluap1/IFT38 Reveals a New Role in Actin Arrangement. Beyer et al (2018). *Mol Cell Proteomics*

Physical Interactions with 213 interactions from BioGRID

Fogeron-Lange-2013 0.34%

LGALS3BP regulates centriole biogenesis and centrosome hypertrophy in cancer cells. Fogeron et al (2013). *Nat Commun*

Physical Interactions with 1,492 interactions from BioGRID

Phillips-Corn-2013 0.33%

Conformational dynamics control ubiquitin-deubiquitinase interactions and influence in vivo signaling. Phillips et al (2013). *Proc Natl Acad Sci U S A*

Physical Interactions with 142 interactions from BioGRID

IREF-hpidb 0.33%

Physical Interactions with 166 interactions from iRefIndex

Diner-Cristea-2015 0.33%

Interactions of the Antiviral Factor Interferon Gamma-Inducible Protein 16 (IFI16) Mediate Immune Signaling and Herpes Simplex Virus-1 Immunosuppression. Diner et al (2015). *Mol Cell Proteomics*

Physical Interactions with 334 interactions from BioGRID

Malovannaya-Qin-2010 0.32%

Streamlined analysis schema for high-throughput identification of endogenous protein complexes. Malovannaya et al (2010). *Proc Natl Acad Sci U S A*

Physical Interactions with 299 interactions from BioGRID

Barr-Knapp-2009 0.32%

Large-scale structural analysis of the classical human protein tyrosine phosphatome. Barr et al (2009). *Cell*

Physical Interactions with 173 interactions from iRefIndex

Faust-Frankel-2018 0.32%

The HIV-1 Tat protein recruits a ubiquitin ligase to reorganize the 7SK snRNP for transcriptional activation. Faust et al (2018). *Elife*

Physical Interactions with 2,200 interactions from BioGRID

Pladevall-Morera-Lopez-Contreras-2019 0.32%

Proteomic characterization of chromosomal common fragile site (CFS)-associated proteins uncovers ATRX as a regulator of CFS stability. Pladevall-Morera et al (2019). *Nucleic Acids Res*

Physical Interactions with 621 interactions from BioGRID

Physical Interactions 77.64%

Fenner-Prehn-2010 0.30%

Expanding the substantial interactome of NEMO using protein microarrays. Fenner et al (2010). *PLoS One*

Physical Interactions with 103 interactions from iRefIndex

Keller-Lee-2014 0.29%

SAICAR induces protein kinase activity of PKM2 that is necessary for sustained proliferative signaling of cancer cells. Keller et al (2014). *Mol Cell*

Physical Interactions with 147 interactions from iRefIndex

Persaud-Rotin-2009 B 0.29%

Comparison of substrate specificity of the ubiquitin ligases Nedd4 and Nedd4-2 using proteome arrays. Persaud et al (2009). *Mol Syst Biol*

Physical Interactions with 155 interactions from BioGRID

Oliviero-Cagney-2016 0.29%

Dynamic Protein Interactions of the Polycomb Repressive Complex 2 during Differentiation of Pluripotent Cells. Oliviero et al (2016). *Mol Cell Proteomics*

Physical Interactions with 638 interactions from BioGRID

Nassa-Weisz-2019 0.29%

The RNA-mediated estrogen receptor   interactome of hormone-dependent human breast cancer cell nuclei. Nassa et al (2019). *Sci Data*

Physical Interactions with 1,490 interactions from BioGRID

Weinmann-Meister-2009 0.28%

Importin 8 is a gene silencing factor that targets argonaute proteins to distinct mRNAs. Weinmann et al (2009). *Cell*

Physical Interactions with 96 interactions from BioGRID

Yang-Brasier-2015 0.28%

Systematic Determination of Human Cyclin Dependent Kinase (CDK)-9 Interactome Identifies Novel Functions in RNA Splicing Mediated by the DEAD Box (DDX)-5/17 RNA Helicases. Yang et al (2015). *Mol Cell Proteomics*

Physical Interactions with 195 interactions from iRefIndex

Kristensen-Foster-2012 0.28%

A high-throughput approach for measuring temporal changes in the interactome. Kristensen et al (2012). *Nat Methods*

Physical Interactions with 7,044 interactions from BioGRID

Behzadnia-Lührmann-2007 0.27%

Composition and three-dimensional EM structure of double affinity-purified, human prespliceosomal A complexes. Behzadnia et al (2007). *EMBO J*

Physical Interactions with 107 interactions from iRefIndex

Llères-Lamond-2010 0.26%

Direct interaction between hnRNP-M and CDC5L/PLRG1 proteins affects alternative splice site choice. Llères et al (2010). *EMBO Rep*

Physical Interactions with 848 interactions from BioGRID

Weishäupl-Schmidt-2019 0.26%

Physiological and pathophysiological characteristics of ataxin-3 isoforms. Weishäupl et al (2019). *J Biol Chem*

Physical Interactions with 202 interactions from iRefIndex

Varjosalo-Gstaiger-2013 B 0.25%

Varjosalo-Gstaiger-2013 B

The protein interaction landscape of the human CMGC kinase group. Varjosalo et al (2013). *Cell Rep*

Physical Interactions with 308 interactions from BioGRID

Hein-Mann-2015 0.24%

A human interactome in three quantitative dimensions organized by stoichiometries and abundances. Hein et al (2015). *Cell*

Physical Interactions with 27,015 interactions from BioGRID

Hosp-Selbach-2015 0.24%

Quantitative interaction proteomics of neurodegenerative disease proteins. Hosp et al (2015). *Cell Rep*

Physical Interactions with 365 interactions from BioGRID

Hutchins-Peters-2010 0.24%

Systematic analysis of human protein complexes identifies chromosome segregation proteins. Hutchins et al (2010). *Science*

Physical Interactions with 1,783 interactions from BioGRID

Greco-Cristea-2011 0.24%

Nuclear import of histone deacetylase 5 by requisite nuclear localization signal phosphorylation. Greco et al (2011). *Mol Cell Proteomics*

Physical Interactions with 256 interactions from BioGRID

Thompson-Luchansky-2014 0.24%

Quantitative Lys- -Gly-Gly (diGly) proteomics coupled with inducible RNAi reveals ubiquitin-mediated proteolysis of DNA damage-inducible transcript 4 (DDIT4) by the E3 ligase HUWE1. Thompson et al (2014). *J Biol Chem*

Physical Interactions with 244 interactions from iRefIndex

Xiao-Lefkowitz-2007 0.24%

Functional specialization of beta-arrestin interactions revealed by proteomic analysis. Xiao et al (2007). *Proc Natl Acad Sci U S A*

Physical Interactions with 404 interactions from BioGRID

Gloeckner-Ueffing-2007 0.23%

A novel tandem affinity purification strategy for the efficient isolation and characterisation of native protein complexes. Gloeckner et al (2007). *Proteomics*

Physical Interactions with 100 interactions from BioGRID

Brehme-Superti-Furga-2009 0.23%

Charting the molecular network of the drug target Bcr-Abl. Brehme et al (2009). *Proc Natl Acad Sci U S A*

Physical Interactions with 626 interactions from BioGRID

Jones-MacBeath-2006 0.23%

A quantitative protein interaction network for the ErbB receptors using protein microarrays. Jones et al (2006). *Nature*

Physical Interactions with 158 interactions from iRefIndex

Scott-Schulman-2016 0.22%

Two Distinct Types of E3 Ligases Work in Unison to Regulate Substrate Ubiquitylation. Scott et al (2016). *Cell*

Physical Interactions with 111 interactions from BioGRID

Yang-Maurer-2018 0.22%

rec-YnH enables simultaneous many-by-many detection of direct protein-protein and protein-RNA interactions. Yang et al (2018). *Nat Commun*

Physical Interactions with 325 interactions from BioGRID

Physical Interactions 77.64%

Xu-Zetter-2016 0.22%

Prohibitin 1 regulates tumor cell apoptosis via the interaction with X-linked inhibitor of apoptosis protein. Xu et al (2016). *J Mol Cell Biol*

Physical Interactions with 719 interactions from BioGRID

IREF-quickgo 0.22%

Physical Interactions with 9,985 interactions from iRefIndex

Van Quickelberghe-Gevaert-2018 0.22%

A protein-protein interaction map of the TNF-induced NF- Bsignal transduction pathway. Van Quickelberghe et al (2018). *Sci Data*

Physical Interactions with 525 interactions from BioGRID

Mohammed-Carroll-2013 0.22%

Endogenous purification reveals GREB1 as a key estrogen receptor regulatory factor. Mohammed et al (2013). *Cell Rep*

Physical Interactions with 112 interactions from BioGRID

IREF-intcomplex 0.22%

Physical Interactions with 212 interactions from iRefIndex

IREF-dip 0.21%

Physical Interactions with 5,037 interactions from iRefIndex

Yang-Wang-2018 0.21%

E3 Ligase Trim21 Ubiquitylates and Stabilizes Keratin 17 to Induce STAT3 Activation in Psoriasis. Yang et al (2018). *J Invest Dermatol*

Physical Interactions with 103 interactions from iRefIndex

Tiemann-Kani-2019 0.21%

Loss of ER retention motif of AGR2 can impact mTORC signaling and promote cancer metastasis. Tiemann et al (2019). *Oncogene*

Physical Interactions with 339 interactions from BioGRID

IREF-mbinfo 0.21%

Physical Interactions with 113 interactions from iRefIndex

Wan-Emili-2015 0.21%

Panorama of ancient metazoan macromolecular complexes. Wan et al (2015). *Nature*

Physical Interactions with 16,627 interactions from BioGRID

Herr-Helleday-2015 0.20%

A genome-wide IR-induced RAD51 foci RNAi screen identifies CDC73 involved in chromatin remodeling for DNA repair. Herr et al (2015). *Cell Discov*

Physical Interactions with 117 interactions from iRefIndex

Gupta-Pelletier-2015 0.20%

A Dynamic Protein Interaction Landscape of the Human Centrosome-Cilium Interface. Gupta et al (2015). *Cell*

Physical Interactions with 306 interactions from BioGRID

Havugimana-Emili-2012 0.20%

A census of human soluble protein complexes. Havugimana et al (2012). *Cell*

Physical Interactions with 13,651 interactions from BioGRID

Physical Interactions 77.64%

Kaltenbach-Hughes-2007 0.20%

Huntingtin interacting proteins are genetic modifiers of neurodegeneration. Kaltenbach et al (2007). *PLoS Genet*

Physical Interactions with 101 interactions from iRefIndex

Fonseca-Damgaard-2015 0.19%

La-related Protein 1 (LARP1) Represses Terminal Oligopyrimidine (TOP) mRNA Translation Downstream of mTOR Complex 1 (mTORC1). Fonseca et al (2015). *J Biol Chem*

Physical Interactions with 105 interactions from BioGRID

Devarajan-Ketha-Kumar-2012 0.19%

The sclerostin-bone protein interactome. Devarajan-Ketha et al (2012). *Biochem Biophys Res Commun*

Physical Interactions with 99 interactions from BioGRID

Tang-Wang-2019 0.19%

The p300/YY1/miR-500a-5p/HDAC2 signalling axis regulates cell proliferation in human colorectal cancer. Tang et al (2019). *Nat Commun*

Physical Interactions with 294 interactions from BioGRID

Barrios-Rodiles-Wrana-2005 0.19%

High-throughput mapping of a dynamic signaling network in mammalian cells. Barrios-Rodiles et al (2005). *Science*

Physical Interactions with 596 interactions from iRefIndex

Salvetti-Greco-2016 0.19%

Nuclear Functions of Nucleolin through Global Proteomics and Interactomic Approaches. Salvetti et al (2016). *J Proteome Res*

Physical Interactions with 144 interactions from BioGRID

Malinová-Verheggen-2017 0.19%

Assembly of the U5 snRNP component PRPF8 is controlled by the HSP90/R2TP chaperones. Malinová et al (2017). *J Cell Biol*

Physical Interactions with 2,778 interactions from BioGRID

Jeronimo-Coulombe-2007 0.19%

Systematic analysis of the protein interaction network for the human transcription machinery reveals the identity of the 7SK capping enzyme. Jeronimo et al (2007). *Mol Cell*

Physical Interactions with 699 interactions from BioGRID

Choudhury-Michlewski-2017 0.19%

RNA-binding activity of TRIM25 is mediated by its PRY/SPRY domain and is required for ubiquitination. Choudhury et al (2017). *BMC Biol*

Physical Interactions with 250 interactions from BioGRID

Lu-Bohr-2017 0.19%

Cell cycle-dependent phosphorylation regulates RECQL4 pathway choice and ubiquitination in DNA double-strand break repair. Lu et al (2017). *Nat Commun*

Physical Interactions with 1,312 interactions from BioGRID

Hubel-Pichlmair-2019 0.19%

A protein-interaction network of interferon-stimulated genes extends the innate immune system landscape. Hubel et al (2019). *Nat Immunol*

Physical Interactions with 2,707 interactions from BioGRID

Moon-Kim-2014 0.18%

Interactome analysis of AMP-activated protein kinase (AMPK)- 1and - 1in INS-1 pancreatic beta-cells by affinity purification-

Physical Interactions 77.64%

Moon-Kim-2014

mass spectrometry. Moon et al (2014). *Sci Rep*

Physical Interactions with 171 interactions from BioGRID

Woods-Monteiro-2012 A 0.18%

Charting the landscape of tandem BRCT domain-mediated protein interactions. Woods et al (2012). *Sci Signal*

Physical Interactions with 602 interactions from BioGRID

Rowbotham-Mermoud-2011 0.18%

Maintenance of silent chromatin through replication requires SWI/SNF-like chromatin remodeler SMARCAD1. Rowbotham et al (2011). *Mol Cell*

Physical Interactions with 114 interactions from BioGRID

Loch-Strickler-2012 0.18%

A microarray of ubiquitylated proteins for profiling deubiquitylase activity reveals the critical roles of both chain and substrate. Loch et al (2012). *Biochim Biophys Acta*

Physical Interactions with 145 interactions from BioGRID

Rosenbluh-Hahn-2016 0.18%

Genetic and Proteomic Interrogation of Lower Confidence Candidate Genes Reveals Signaling Networks in -Catenin-Active Cancers. Rosenbluh et al (2016). *Cell Syst*

Physical Interactions with 3,482 interactions from BioGRID

Liebelt-Vertegaal-2020 0.18%

Transcription-coupled nucleotide excision repair is coordinated by ubiquitin and SUMO in response to ultraviolet irradiation. Liebelt et al (2020). *Nucleic Acids Res*

Physical Interactions with 100 interactions from BioGRID

Noguchi-Kawahara-2018 0.18%

ZFP36L2 is a cell cycle-regulated CCCH protein necessary for DNA lesion-induced S-phase arrest. Noguchi et al (2018). *Biol Open*

Physical Interactions with 143 interactions from iRefIndex

Gilmore-Washburn-2016 0.18%

WDR76 Co-Localizes with Heterochromatin Related Proteins and Rapidly Responds to DNA Damage. Gilmore et al (2016). *PLoS One*

Physical Interactions with 944 interactions from BioGRID

Agrawal-Sedivy-2010 0.17%

Proteomic profiling of Myc-associated proteins. Agrawal et al (2010). *Cell Cycle*

Physical Interactions with 105 interactions from BioGRID

Cai-Conaway-2007 0.17%

YY1 functions with INO80 to activate transcription. Cai et al (2007). *Nat Struct Mol Biol*

Physical Interactions with 106 interactions from BioGRID

Lee-Mayr-2019 0.17%

Gain of Additional BIRC3 Protein Functions through 3'-UTR-Mediated Protein Complex Formation. Lee et al (2019). *Mol Cell*

Physical Interactions with 1,759 interactions from BioGRID

Lee-Jeong-2017 0.17%

FGF11 induced by hypoxia interacts with HIF-1 and enhances its stability. Lee et al (2017). *FEBS Lett*

Physical Interactions 77.64%

Lee-Jeong-2017

Physical Interactions with 100 interactions from iRefIndex

Oláh-Ovádi-2011 0.17%

Interactions of pathological hallmark proteins: tubulin polymerization promoting protein/p25, beta-amyloid, and alpha-synuclein. Oláh et al (2011). *J Biol Chem*

Physical Interactions with 1,854 interactions from BioGRID

Roewenstrunk-de la Luna-2019 0.17%

A comprehensive proteomics-based interaction screen that links DYRK1A to RNF169 and to the DNA damage response. Roewenstrunk et al (2019). *Sci Rep*

Physical Interactions with 116 interactions from BioGRID

Mugabo-Lim-2018 0.17%

Elucidation of the 14-3-3 interactome reveals critical roles of RNA-splicing factors during adipogenesis. Mugabo et al (2018). *J Biol Chem*

Physical Interactions with 111 interactions from BioGRID

Shami Shah-Baskin-2019 0.16%

PLEKHA4/kramer Attenuates Dishevelled Ubiquitination to Modulate Wnt and Planar Cell Polarity Signaling. Shami Shah et al (2019). *Cell Rep*

Physical Interactions with 2,927 interactions from BioGRID

Yao-Stagljar-2017 A 0.16%

A Global Analysis of the Receptor Tyrosine Kinase-Protein Phosphatase Interactome. Yao et al (2017). *Mol Cell*

Physical Interactions with 312 interactions from BioGRID

Emdal-Olsen-2015 0.16%

Temporal proteomics of NGF-TrkA signaling identifies an inhibitory role for the E3 ligase Cbl-b in neuroblastoma cell differentiation. Emdal et al (2015). *Sci Signal*

Physical Interactions with 1,917 interactions from BioGRID

Zhang-Wheeler-2014 0.16%

Progesterone receptor membrane component 1 is a functional part of the glucagon-like peptide-1 (GLP-1) receptor complex in pancreatic   cells. Zhang et al (2014). *Mol Cell Proteomics*

Physical Interactions with 93 interactions from BioGRID

Tong-Moran-2014 0.16%

Proteomic analysis of the epidermal growth factor receptor (EGFR) interactome and post-translational modifications associated with receptor endocytosis in response to EGF and stress. Tong et al (2014). *Mol Cell Proteomics*

Physical Interactions with 321 interactions from BioGRID

Yue-Liu-2018 0.16%

VIRMA mediates preferential m⁶A mRNA methylation in 3'UTR and near stop codon and associates with alternative polyadenylation. Yue et al (2018). *Cell Discov*

Physical Interactions with 1,502 interactions from BioGRID

Papp-Lamia-2015 0.15%

DNA damage shifts circadian clock time via Hausp-dependent Cry1 stabilization. Papp et al (2015). *Elife*

Physical Interactions with 158 interactions from iRefIndex

Cheng-DeCaprio-2017 0.15%

Merkel cell polyomavirus recruits MYCL to the EP400 complex to promote oncogenesis. Cheng et al (2017). *PLoS Pathog*

Physical Interactions 77.64%

Cheng-DeCaprio-2017

Physical Interactions with 95 interactions from BioGRID

Ramachandran-LaBaer-2004 0.15%

Self-assembling protein microarrays. Ramachandran et al (2004). *Science*

Physical Interactions with 123 interactions from iRefIndex

Low-Heck-2014 0.15%

A systems-wide screen identifies substrates of the SCF TrCP ubiquitin ligase. Low et al (2014). *Sci Signal*

Physical Interactions with 221 interactions from BioGRID

Liu-Takahashi-2017 0.15%

TTF-1/NKX2-1 binds to DDB1 and confers replication stress resistance to lung adenocarcinomas. Liu et al (2017). *Oncogene*

Physical Interactions with 105 interactions from iRefIndex

Roy-Pardo-2014 0.15%

hnRNPA1 couples nuclear export and translation of specific mRNAs downstream of FGF-2/S6K2 signalling. Roy et al (2014). *Nucleic Acids Res*

Physical Interactions with 386 interactions from BioGRID

Li-Dorf-2011 A 0.15%

Mapping a dynamic innate immunity protein interaction network regulating type I interferon production. Li et al (2011). *Immunity*

Physical Interactions with 400 interactions from BioGRID

Giannone-Liu-2010 0.14%

The protein network surrounding the human telomere repeat binding factors TRF1, TRF2, and POT1. Giannone et al (2010). *PLoS One*

Physical Interactions with 288 interactions from BioGRID

Choi-Busino-2018 0.14%

PTPN14 regulates Roquin2 stability by tyrosine dephosphorylation. Choi et al (2018). *Cell Cycle*

Physical Interactions with 1,087 interactions from BioGRID

Meek-Piwnica-Worms-2004 0.14%

Comprehensive proteomic analysis of interphase and mitotic 14-3-3-binding proteins. Meek et al (2004). *J Biol Chem*

Physical Interactions with 328 interactions from iRefIndex

Wang-Balch-2006 0.14%

Hsp90 cochaperone Aha1 downregulation rescues misfolding of CFTR in cystic fibrosis. Wang et al (2006). *Cell*

Physical Interactions with 200 interactions from BioGRID

Lum-Cristea-2018 0.14%

Interactome and Proteome Dynamics Uncover Immune Modulatory Associations of the Pathogen Sensing Factor cGAS. Lum et al (2018). *Cell Syst*

Physical Interactions with 165 interactions from iRefIndex

IREF-uniprotpp 0.14%

Physical Interactions with 2,470 interactions from iRefIndex

Hu-Yin-2019 0.14%

Poly(ADP-ribosyl)ation of BRD7 by PARP1 confers resistance to DNA-damaging chemotherapeutic agents. Hu et al (2019).

Physical Interactions 77.64%

Hu-Yin-2019

EMBO Rep

Physical Interactions with 479 interactions from BioGRID

BIOGRID-SMALL-SCALE-STUDIES 0.14%

Physical Interactions with 79,201 interactions from BioGRID

Wallach-Kramer-2013 0.14%

Dynamic circadian protein-protein interaction networks predict temporal organization of cellular functions. Wallach et al (2013). *PLoS Genet*

Physical Interactions with 143 interactions from BioGRID

Wu-Li-2007 0.14%

Systematic identification of SH3 domain-mediated human protein-protein interactions by peptide array target screening. Wu et al (2007). *Proteomics*

Physical Interactions with 1,105 interactions from iRefIndex

Floyd-Pagliarini-2016 0.14%

Mitochondrial Protein Interaction Mapping Identifies Regulators of Respiratory Chain Function. Floyd et al (2016). *Mol Cell*

Physical Interactions with 1,508 interactions from BioGRID

Moutaoufik-Babu-2019 0.13%

Rewiring of the Human Mitochondrial Interactome during Neuronal Reprogramming Reveals Regulators of the Respirasome and Neurogenesis. Moutaoufik et al (2019). *iScience*

Physical Interactions with 6,357 interactions from BioGRID

Sowa-Harper-2009 0.13%

Defining the human deubiquitinating enzyme interaction landscape. Sowa et al (2009). *Cell*

Physical Interactions with 1,509 interactions from BioGRID

Woodsmith-Sanderson-2012 0.13%

Systematic analysis of dimeric E3-RING interactions reveals increased combinatorial complexity in human ubiquitination networks. Woodsmith et al (2012). *Mol Cell Proteomics*

Physical Interactions with 206 interactions from iRefIndex

IREF-innatedb 0.13%

Physical Interactions with 2,355 interactions from iRefIndex

IREF-mppi 0.13%

Physical Interactions with 304 interactions from iRefIndex

Bennett-Harper-2010 0.13%

Dynamics of cullin-RING ubiquitin ligase network revealed by systematic quantitative proteomics. Bennett et al (2010). *Cell*

Physical Interactions with 4,362 interactions from BioGRID

Humphries-Humphries-2009 0.13%

Proteomic analysis of integrin-associated complexes identifies RCC2 as a dual regulator of Rac1 and Arf6. Humphries et al (2009). *Sci Signal*

Physical Interactions with 1,060 interactions from BioGRID

McCracken-Blencowe-2005 0.12%

Proteomic analysis of SRm160-containing complexes reveals a conserved association with cohesin. McCracken et al (2005). *J Biol*

Physical Interactions 77.64%

McCracken-Blencowe-2005

Chem

Physical Interactions with 198 interactions from BioGRID

Boldt-Roepman-2016 0.12%

An organelle-specific protein landscape identifies novel diseases and molecular mechanisms. Boldt et al (2016). *Nat Commun*

Physical Interactions with 4,898 interactions from BioGRID

Crow-Cristea-2017 0.12%

Human Antiviral Protein IFIX Suppresses Viral Gene Expression during Herpes Simplex Virus 1 (HSV-1) Infection and Is Counteracted by Virus-induced Proteasomal Degradation. Crow et al (2017). *Mol Cell Proteomics*

Physical Interactions with 180 interactions from iRefIndex

Alexander-Wang-2018 0.12%

Ubiquilin 2 modulates ALS/FTD-linked FUS-RNA complex dynamics and stress granule formation. Alexander et al (2018). *Proc Natl Acad Sci U S A*

Physical Interactions with 189 interactions from iRefIndex

Arroyo-Aloy-2014 0.12%

Charting the molecular links between driver and susceptibility genes in colorectal cancer. Arroyo et al (2014). *Biochem Biophys Res Commun*

Physical Interactions with 621 interactions from iRefIndex

Bett-Hay-2013 0.12%

The P-body component USP52/PAN2 is a novel regulator of HIF1A mRNA stability. Bett et al (2013). *Biochem J*

Physical Interactions with 238 interactions from iRefIndex

Cox-Rizzino-2013 0.12%

The SOX2-interactome in brain cancer cells identifies the requirement of MSI2 and USP9X for the growth of brain tumor cells. Cox et al (2013). *PLoS One*

Physical Interactions with 190 interactions from iRefIndex

Pao-Virdee-2018 0.11%

Activity-based E3 ligase profiling uncovers an E3 ligase with esterification activity. Pao et al (2018). *Nature*

Physical Interactions with 134 interactions from BioGRID

Bandyopadhyay-Ideker-2010 0.11%

A human MAP kinase interactome. Bandyopadhyay et al (2010). *Nat Methods*

Physical Interactions with 653 interactions from iRefIndex

Zhao-Krug-2005 0.11%

Human ISG15 conjugation targets both IFN-induced and constitutively expressed proteins functioning in diverse cellular pathways. Zhao et al (2005). *Proc Natl Acad Sci U S A*

Physical Interactions with 140 interactions from iRefIndex

Enzo-Dupont-2015 0.10%

Aerobic glycolysis tunes YAP/TAZ transcriptional activity. Enzo et al (2015). *EMBO J*

Physical Interactions with 156 interactions from BioGRID

Huttlin-Harper-2017 0.10%

Architecture of the human interactome defines protein communities and disease networks. Huttlin et al (2017). *Nature*

Physical Interactions with 55,868 interactions from BioGRID

Physical Interactions 77.64%

Napolitano-Meroni-2011 0.10%

Functional interactions between ubiquitin E2 enzymes and TRIM proteins. Napolitano et al (2011). *Biochem J*

Physical Interactions with 81 interactions from BioGRID

Varjosalo-Superti-Furga-2013 0.10%

Interlaboratory reproducibility of large-scale human protein-complex analysis by standardized AP-MS. Varjosalo et al (2013). *Nat Methods*

Physical Interactions with 484 interactions from BioGRID

Chi-Reed-2018 0.10%

Interactome analyses revealed that the U1 snRNP machinery overlaps extensively with the RNAP II machinery and contains multiple ALS/SMA-causative proteins. Chi et al (2018). *Sci Rep*

Physical Interactions with 456 interactions from BioGRID

Li-Lu-2018 0.10%

Heterozygous deletion of chromosome 17p renders prostate cancer vulnerable to inhibition of RNA polymerase II. Li et al (2018). *Nat Commun*

Physical Interactions with 176 interactions from iRefIndex

Viita-Vartiainen-2019 0.10%

Nuclear actin interactome analysis links actin to KAT14 histone acetyl transferase and mRNA splicing. Viita et al (2019). *J Cell Sci*

Physical Interactions with 213 interactions from BioGRID

Banks-Washburn-2016 0.09%

TNIP2 is a Hub Protein in the NF- BNetwork with Both Protein and RNA Mediated Interactions. Banks et al (2016). *Mol Cell Proteomics*

Physical Interactions with 616 interactions from BioGRID

Tarallo-Weisz-2011 0.09%

Identification of proteins associated with ligand-activated estrogen receptor in human breast cancer cell nuclei by tandem affinity purification and nano LC-MS/MS. Tarallo et al (2011). *Proteomics*

Physical Interactions with 244 interactions from BioGRID

Yamauchi-Maeda-2018 0.09%

Genome-wide CRISPR-Cas9 Screen Identifies Leukemia-Specific Dependence on a Pre-mRNA Metabolic Pathway Regulated by DCPS. Yamauchi et al (2018). *Cancer Cell*

Physical Interactions with 261 interactions from iRefIndex

Douanne-Bidère-2019 0.09%

CYLD Regulates Centriolar Satellites Proteostasis by Counteracting the E3 Ligase MIB1. Douanne et al (2019). *Cell Rep*

Physical Interactions with 111 interactions from iRefIndex

Panigrahi-Pati-2012 0.09%

A cohesin-RAD21 interactome. Panigrahi et al (2012). *Biochem J*

Physical Interactions with 137 interactions from iRefIndex

Joshi-Cristea-2013 0.09%

The functional interactome landscape of the human histone deacetylase family. Joshi et al (2013). *Mol Syst Biol*

Physical Interactions with 375 interactions from BioGRID

Katsogiannou-Rocchi-2014 0.09%

Physical Interactions 77.64%

Katsogiannou-Rocchi-2014

The functional landscape of Hsp27 reveals new cellular processes such as DNA repair and alternative splicing and proposes novel anticancer targets. Katsogiannou et al (2014). *Mol Cell Proteomics*

Physical Interactions with 221 interactions from iRefIndex

Trepte-Wanker-2018 A 0.09%

LuTHy: a double-readout bioluminescence-based two-hybrid technology for quantitative mapping of protein-protein interactions in mammalian cells. Trepte et al (2018). *Mol Syst Biol*

Physical Interactions with 70 interactions from BioGRID

Lau-Ronai-2012 0.09%

PKC promotes oncogenic functions of ATF2 in the nucleus while blocking its apoptotic function at mitochondria. Lau et al (2012). *Cell*

Physical Interactions with 102 interactions from iRefIndex

Campos-Reinberg-2015 0.09%

Analysis of the Histone H3.1 Interactome: A Suitable Chaperone for the Right Event. Campos et al (2015). *Mol Cell*

Physical Interactions with 105 interactions from iRefIndex

Malty-Babu-2017 0.09%

A Map of Human Mitochondrial Protein Interactions Linked to Neurodegeneration Reveals New Mechanisms of Redox Homeostasis and NF- B Signaling. Malty et al (2017). *Cell Syst*

Physical Interactions with 1,969 interactions from BioGRID

Roy-Parent-2013 0.09%

Novel, gel-free proteomics approach identifies RNF5 and JAMP as modulators of GPCR stability. Roy et al (2013). *Mol Endocrinol*

Physical Interactions with 114 interactions from iRefIndex

Christianson-Kopito-2011 0.09%

Defining human ERAD networks through an integrative mapping strategy. Christianson et al (2011). *Nat Cell Biol*

Physical Interactions with 294 interactions from BioGRID

Li-Dorf-2014 0.09%

TRIM65 regulates microRNA activity by ubiquitination of TNRC6. Li et al (2014). *Proc Natl Acad Sci U S A*

Physical Interactions with 495 interactions from BioGRID

Behrends-Harper-2010 0.09%

Network organization of the human autophagy system. Behrends et al (2010). *Nature*

Physical Interactions with 704 interactions from BioGRID

Drissi-Boisvert-2015 0.09%

Quantitative Proteomics Reveals Dynamic Interactions of the Minichromosome Maintenance Complex (MCM) in the Cellular Response to Etoposide Induced DNA Damage. Drissi et al (2015). *Mol Cell Proteomics*

Physical Interactions with 947 interactions from BioGRID

Foerster-Ritter-2013 0.08%

Characterization of the EGFR interactome reveals associated protein complex networks and intracellular receptor dynamics. Foerster et al (2013). *Proteomics*

Physical Interactions with 179 interactions from BioGRID

Li-Chen-2015 0.08%

Physical Interactions 77.64%

Li-Chen-2015

Proteomic analyses reveal distinct chromatin-associated and soluble transcription factor complexes. Li et al (2015). *Mol Syst Biol*

Physical Interactions with 1,811 interactions from BioGRID

Lipp-Guthrie-2015 0.08%

SR protein kinases promote splicing of nonconsensus introns. Lipp et al (2015). *Nat Struct Mol Biol*

Physical Interactions with 388 interactions from BioGRID

IREF-mint 0.08%

Physical Interactions with 14,408 interactions from iRefIndex

Kennedy-Kolch-2020 A 0.08%

Extensive rewiring of the EGFR network in colorectal cancer cells expressing transforming levels of KRAS^{G13D}. Kennedy et al (2020). *Nat Commun*

Physical Interactions with 4,232 interactions from BioGRID

IREF-corum 0.08%

Physical Interactions with 819 interactions from iRefIndex

Kärblane-Sarmiento-2015 0.07%

ABCE1 is a highly conserved RNA silencing suppressor. Kärblane et al (2015). *PLoS One*

Physical Interactions with 140 interactions from iRefIndex

Wong-O'Bryan-2012 0.07%

Intersectin (ITSN) family of scaffolds function as molecular hubs in protein interaction networks. Wong et al (2012). *PLoS One*

Physical Interactions with 114 interactions from BioGRID

Li-Zhou-2017 0.07%

Identification of translationally controlled tumor protein in promotion of DNA homologous recombination repair in cancer cells by affinity proteomics. Li et al (2017). *Oncogene*

Physical Interactions with 104 interactions from iRefIndex

Wang-Xu-2015 0.07%

Interaction of amyotrophic lateral sclerosis/frontotemporal lobar degeneration-associated fused-in-sarcoma with proteins involved in metabolic and protein degradation pathways. Wang et al (2015). *Neurobiol Aging*

Physical Interactions with 197 interactions from BioGRID

Saito-Kobarg-2017 0.07%

Human Regulatory Protein Ki-1/57 Is a Target of SUMOylation and Affects PML Nuclear Body Formation. Saito et al (2017). *J Proteome Res*

Physical Interactions with 165 interactions from iRefIndex

Dabbaghizadeh-Tanguay-2018 0.07%

Identification of proteins interacting with the mitochondrial small heat shock protein Hsp22 of Drosophila melanogaster: Implication in mitochondrial homeostasis. Dabbaghizadeh et al (2018). *PLoS One*

Physical Interactions with 144 interactions from BioGRID

Zhang-Xu-2018 0.07%

Revealing A-Raf functions through its interactome. Zhang et al (2018). *Biochim Biophys Acta Proteins Proteom*

Physical Interactions with 186 interactions from BioGRID

Hauri-Beisel-2016 0.07%

Physical Interactions 77.64%

Hauri-Beisel-2016

A High-Density Map for Navigating the Human Polycomb Complexome. Hauri et al (2016). *Cell Rep*

Physical Interactions with 1,216 interactions from BioGRID

Ewing-Figeys-2007 0.07%

Large-scale mapping of human protein-protein interactions by mass spectrometry. Ewing et al (2007). *Mol Syst Biol*

Physical Interactions with 5,759 interactions from iRefIndex

Menon-Litovchick-2019 0.07%

DYRK1A regulates the recruitment of 53BP1 to the sites of DNA damage in part through interaction with RNF169. Menon et al (2019). *Cell Cycle*

Physical Interactions with 119 interactions from BioGRID

IREF-bind-translation 0.07%

Physical Interactions with 6,056 interactions from iRefIndex

Hayes-Urbé-2012 0.06%

Direct and indirect control of mitogen-activated protein kinase pathway-associated components, BRAP/IMP E3 ubiquitin ligase and CRAF/RAF1 kinase, by the deubiquitylating enzyme USP15. Hayes et al (2012). *J Biol Chem*

Physical Interactions with 110 interactions from BioGRID

Reyniers-Taymans-2014 0.06%

Differential protein-protein interactions of LRRK1 and LRRK2 indicate roles in distinct cellular signaling pathways. Reyniers et al (2014). *J Neurochem*

Physical Interactions with 109 interactions from iRefIndex

Botham-Schimmer-2019 0.06%

Global Interactome Mapping of Mitochondrial Intermembrane Space Proteases Identifies a Novel Function for HTRA2. Botham et al (2019). *Proteomics*

Physical Interactions with 317 interactions from BioGRID

Kim-Major-2015 0.06%

Substrate trapping proteomics reveals targets of the TrCP2/FBXW11 ubiquitin ligase. Kim et al (2015). *Mol Cell Biol*

Physical Interactions with 137 interactions from BioGRID

Grossmann-Stelzl-2015 0.06%

Phospho-tyrosine dependent protein-protein interaction network. Grossmann et al (2015). *Mol Syst Biol*

Physical Interactions with 620 interactions from BioGRID

IREF-bind 0.06%

Physical Interactions with 3,524 interactions from iRefIndex

Bouwmeester-Superti-Furga-2004 0.06%

A physical and functional map of the human TNF-alpha/NF-kappa B signal transduction pathway. Bouwmeester et al (2004). *Nat Cell Biol*

Physical Interactions with 1,694 interactions from iRefIndex

St-Denis-Gingras-2015 0.05%

Myotubularin-related proteins 3 and 4 interact with polo-like kinase 1 and centrosomal protein of 55 kDa to ensure proper abscission. St-Denis et al (2015). *Mol Cell Proteomics*

Physical Interactions with 168 interactions from BioGRID

0.05%

Physical Interactions 77.64%

Blomen-Brummelkamp-2015

Gene essentiality and synthetic lethality in haploid human cells. Blomen et al (2015). *Science*

Physical Interactions with 138 interactions from BioGRID

Li-Haura-2013 0.05%

Perturbation of the mutated EGFR interactome identifies vulnerabilities and resistance mechanisms. Li et al (2013). *Mol Syst Biol*

Physical Interactions with 403 interactions from BioGRID

Liu-Chen-2019 0.05%

Oncogenic functions of protein kinase D2 and D3 in regulating multiple cancer-related pathways in breast cancer. Liu et al (2019). *Cancer Med*

Physical Interactions with 106 interactions from BioGRID

Liu-Yang-2019 0.05%

Inflammation-dependent overexpression of c-Myc enhances CRL4^{DCAF4} E3 ligase activity and promotes ubiquitination of ST7 in colitis-associated cancer. Liu et al (2019). *J Pathol*

Physical Interactions with 279 interactions from BioGRID

Oliviero-Cagney-2015 0.05%

The variant Polycomb Repressor Complex 1 component PCGF1 interacts with a pluripotency sub-network that includes DPPA4, a regulator of embryogenesis. Oliviero et al (2015). *Sci Rep*

Physical Interactions with 677 interactions from BioGRID

Huttlin-Gygi-2015 0.05%

The BioPlex Network: A Systematic Exploration of the Human Interactome. Huttlin et al (2015). *Cell*

Physical Interactions with 23,384 interactions from BioGRID

Watanabe-Fujita-2018 0.05%

GRWD1 regulates ribosomal protein L23 levels via the ubiquitin-proteasome system. Watanabe et al (2018). *J Cell Sci*

Physical Interactions with 158 interactions from iRefIndex

Hegele-Stelzl-2012 B 0.05%

Dynamic protein-protein interaction wiring of the human spliceosome. Hegele et al (2012). *Mol Cell*

Physical Interactions with 600 interactions from BioGRID

So-Colwill-2015 0.05%

Integrative analysis of kinase networks in TRAIL-induced apoptosis provides a source of potential targets for combination therapy. So et al (2015). *Sci Signal*

Physical Interactions with 652 interactions from BioGRID

Conte-Perez-Oliva-2018 0.05%

USP45 and Spindly are part of the same complex implicated in cell migration. Conte et al (2018). *Sci Rep*

Physical Interactions with 161 interactions from iRefIndex

Trepte-Wanker-2018 B 0.05%

LuTHy: a double-readout bioluminescence-based two-hybrid technology for quantitative mapping of protein-protein interactions in mammalian cells. Trepte et al (2018). *Mol Syst Biol*

Physical Interactions with 138 interactions from BioGRID

Rual-Vidal-2005 0.04%

Towards a proteome-scale map of the human protein-protein interaction network. Rual et al (2005). *Nature*

Physical Interactions 77.64%

Rual-Vidal-2005

Physical Interactions with 4,031 interactions from iRefIndex

Liu-Tan-2018 0.04%

Proteome-wide analysis of USP14 substrates revealed its role in hepatosteatosis via stabilization of FASN. Liu et al (2018). *Nat Commun*

Physical Interactions with 331 interactions from BioGRID

IREF-matrixdb 0.04%

Physical Interactions with 15,422 interactions from iRefIndex

Huber-Hoelz-2017 0.04%

Histone-binding of DPF2 mediates its repressive role in myeloid differentiation. Huber et al (2017). *Proc Natl Acad Sci U S A*

Physical Interactions with 217 interactions from iRefIndex

Teixeira-Laman-2016 A 0.04%

Gsk3 and Tomm20 are substrates of the SCFFbxo7/PARK15 ubiquitin ligase associated with Parkinson's disease. Teixeira et al (2016). *Biochem J*

Physical Interactions with 130 interactions from BioGRID

Coyaud-Raught-2015 0.04%

BioID-based Identification of Skp Cullin F-box (SCF) -TrCP1/2 E3 Ligase Substrates. Coyaud et al (2015). *Mol Cell Proteomics*

Physical Interactions with 164 interactions from BioGRID

Guard-Old-2019 0.04%

The nuclear interactome of DYRK1A reveals a functional role in DNA damage repair. Guard et al (2019). *Sci Rep*

Physical Interactions with 105 interactions from BioGRID

Rengasamy-Walsh-2017 0.04%

The PRMT5/WDR77 complex regulates alternative splicing through ZNF326 in breast cancer. Rengasamy et al (2017). *Nucleic Acids Res*

Physical Interactions with 103 interactions from iRefIndex

Bantscheff-Drewes-2011 0.04%

Chemoproteomics profiling of HDAC inhibitors reveals selective targeting of HDAC complexes. Bantscheff et al (2011). *Nat Biotechnol*

Physical Interactions with 103 interactions from BioGRID

Asadi-Dhanvantari-2018 0.04%

Plasticity in the Glucagon Interactome Reveals Novel Proteins That Regulate Glucagon Secretion in -TC1-6 Cells. Asadi et al (2018). *Front Endocrinol (Lausanne)*

Physical Interactions with 229 interactions from BioGRID

Giurato-Tarallo-2018 0.04%

Quantitative mapping of RNA-mediated nuclear estrogen receptor interactome in human breast cancer cells. Giurato et al (2018). *Sci Data*

Physical Interactions with 2,161 interactions from BioGRID

Rivera-Paes Leme-2018 0.04%

Agrin has a pathological role in the progression of oral cancer. Rivera et al (2018). *Br J Cancer*

Physical Interactions with 194 interactions from BioGRID

0.04%

Physical Interactions 77.64%

Yao-Stagljar-2017 B

A Global Analysis of the Receptor Tyrosine Kinase-Protein Phosphatase Interactome. Yao et al (2017). *Mol Cell*

Physical Interactions with 325 interactions from BioGRID

Zeller-Wei-2006 0.04%

Global mapping of c-Myc binding sites and target gene networks in human B cells. Zeller et al (2006). *Proc Natl Acad Sci U S A*

Physical Interactions with 634 interactions from iRefIndex

Choi-Beutler-2019 0.04%

LMBR1L regulates lymphopoiesis through Wnt/ -catenin signaling. Choi et al (2019). *Science*

Physical Interactions with 928 interactions from BioGRID

Pankow-Yates-2015 0.04%

F508 CFTR interactome remodelling promotes rescue of cystic fibrosis. Pankow et al (2015). *Nature*

Physical Interactions with 637 interactions from BioGRID

Zhang-Zou-2011 0.04%

A bead-based approach for large-scale identification of in vitro kinase substrates. Zhang et al (2011). *Proteomics*

Physical Interactions with 163 interactions from iRefIndex

van Wijk-Timmers-2009 0.04%

A comprehensive framework of E2-RING E3 interactions of the human ubiquitin-proteasome system. van Wijk et al (2009). *Mol Syst Biol*

Physical Interactions with 322 interactions from iRefIndex

Scholz-Taylor-2016 0.03%

FIH Regulates Cellular Metabolism through Hydroxylation of the Deubiquitinase OTUB1. Scholz et al (2016). *PLoS Biol*

Physical Interactions with 134 interactions from BioGRID

Abbasi-Schild-Poulter-2019 0.03%

Mapping the Ku Interactome Using Proximity-Dependent Biotin Identification in Human Cells. Abbasi et al (2019). *J Proteome Res*

Physical Interactions with 166 interactions from iRefIndex

Pichlmair-Superti-Furga-2011 0.03%

IFIT1 is an antiviral protein that recognizes 5'-triphosphate RNA. Pichlmair et al (2011). *Nat Immunol*

Physical Interactions with 99 interactions from BioGRID

Kahle-Zoghbi-2011 0.03%

Comparison of an expanded ataxia interactome with patient medical records reveals a relationship between macular degeneration and ataxia. Kahle et al (2011). *Hum Mol Genet*

Physical Interactions with 132 interactions from iRefIndex

Wilkinson-Coba-2019 0.03%

Endogenous Cell Type-Specific Disrupted in Schizophrenia 1 Interactomes Reveal Protein Networks Associated With Neurodevelopmental Disorders. Wilkinson et al (2019). *Biol Psychiatry*

Physical Interactions with 100 interactions from iRefIndex

Woods-Monteiro-2012 B 0.03%

Charting the landscape of tandem BRCT domain-mediated protein interactions. Woods et al (2012). *Sci Signal*

Physical Interactions with 325 interactions from BioGRID

Physical Interactions 77.64%

Cloutier-Coulombe-2017 0.03%

R2TP/Prefoldin-like component RUVBL1/RUVBL2 directly interacts with ZNHIT2 to regulate assembly of U5 small nuclear ribonucleoprotein. Cloutier et al (2017). *Nat Commun*

Physical Interactions with 506 interactions from BioGRID

Chen-Guan-2018 0.03%

SNIP1 Recruits TET2 to Regulate c-MYC Target Genes and Cellular DNA Damage Response. Chen et al (2018). *Cell Rep*

Physical Interactions with 240 interactions from BioGRID

Kumar-Vertegaal-2017 0.03%

The STUbL RNF4 regulates protein group SUMOylation by targeting the SUMO conjugation machinery. Kumar et al (2017). *Nat Commun*

Physical Interactions with 1,198 interactions from BioGRID

Lim-Zoghbi-2006 0.03%

A protein-protein interaction network for human inherited ataxias and disorders of Purkinje cell degeneration. Lim et al (2006). *Cell*

Physical Interactions with 5,393 interactions from iRefIndex

Foster-Marshall-2013 0.03%

Proteomic analysis of the NOS2 interactome in human airway epithelial cells. Foster et al (2013). *Nitric Oxide*

Physical Interactions with 111 interactions from iRefIndex

Xu-Ye-2012 0.03%

SGTA recognizes a noncanonical ubiquitin-like domain in the Bag6-Ubl4A-Trc35 complex to promote endoplasmic reticulum-associated degradation. Xu et al (2012). *Cell Rep*

Physical Interactions with 189 interactions from iRefIndex

Jin-Pawson-2004 0.03%

Proteomic, functional, and domain-based analysis of in vivo 14-3-3 binding proteins involved in cytoskeletal regulation and cellular organization. Jin et al (2004). *Curr Biol*

Physical Interactions with 247 interactions from iRefIndex

Soler-López-Aloy-2011 0.02%

Interactome mapping suggests new mechanistic details underlying Alzheimer's disease. Soler-López et al (2011). *Genome Res*

Physical Interactions with 283 interactions from iRefIndex

Rodriguez-von Kriegsheim-2016 0.02%

Substrate-Trapped Interactors of PHD3 and FIH Cluster in Distinct Signaling Pathways. Rodriguez et al (2016). *Cell Rep*

Physical Interactions with 2,035 interactions from BioGRID

IREF-spike 0.02%

Physical Interactions with 20,971 interactions from iRefIndex

Liu-Wang-2012 0.02%

Proteomic identification of common SCF ubiquitin ligase FBXO6-interacting glycoproteins in three kinds of cells. Liu et al (2012). *J Proteome Res*

Physical Interactions with 593 interactions from BioGRID

Chen-Yu-2018 0.02%

An interactome perturbation framework prioritizes damaging missense mutations for developmental disorders. Chen et al (2018). *Nat Genet*

Physical Interactions 77.64%

Chen-Yu-2018

Physical Interactions with 404 interactions from BioGRID

Perez-Hernandez-Yáñez-Mó-2013 0.02%

The intracellular interactome of tetraspanin-enriched microdomains reveals their function as sorting machineries toward exosomes. Perez-Hernandez et al (2013). *J Biol Chem*

Physical Interactions with 446 interactions from BioGRID

Sundell-Ivarsson-2018 0.02%

Proteome-wide analysis of phospho-regulated PDZ domain interactions. Sundell et al (2018). *Mol Syst Biol*

Physical Interactions with 129 interactions from iRefIndex

Hu-Woods-2019 0.02%

CTDP1 regulates breast cancer survival and DNA repair through BRCT-specific interactions with FANCI. Hu et al (2019). *Cell Death Discov*

Physical Interactions with 103 interactions from BioGRID

Wang-Yang-2011 0.02%

Toward an understanding of the protein interaction network of the human liver. Wang et al (2011). *Mol Syst Biol*

Physical Interactions with 3,408 interactions from BioGRID

Varjosalo-Gstaiger-2013 A 0.02%

The protein interaction landscape of the human CMGC kinase group. Varjosalo et al (2013). *Cell Rep*

Physical Interactions with 690 interactions from BioGRID

IREF-huri 0.02%

Physical Interactions with 47,604 interactions from iRefIndex

Yachie-Roth-2016 0.02%

Pooled-matrix protein interaction screens using Barcode Fusion Genetics. Yachie et al (2016). *Mol Syst Biol*

Physical Interactions with 671 interactions from BioGRID

Olma-Pintard-2009 0.01%

An interaction network of the mammalian COP9 signalosome identifies Dda1 as a core subunit of multiple Cul4-based E3 ligases. Olma et al (2009). *J Cell Sci*

Physical Interactions with 213 interactions from BioGRID

Xie-Zhang-2017 0.01%

FAF1 phosphorylation by AKT accumulates TGF- type II receptor and drives breast cancer metastasis. Xie et al (2017). *Nat Commun*

Physical Interactions with 227 interactions from BioGRID

Zhu-Liu-2018 0.01%

Deubiquitinating enzyme PSMD14 promotes tumor metastasis through stabilizing SNAIL in human esophageal squamous cell carcinoma. Zhu et al (2018). *Cancer Lett*

Physical Interactions with 287 interactions from iRefIndex

Bogachek-Weigel-2014 0.01%

Sumoylation pathway is required to maintain the basal breast cancer subtype. Bogachek et al (2014). *Cancer Cell*

Physical Interactions with 134 interactions from iRefIndex

St-Denis-Gingras-2016 0.01%

Physical Interactions 77.64%

St-Denis-Gingras-2016

Phenotypic and Interaction Profiling of the Human Phosphatases Identifies Diverse Mitotic Regulators. St-Denis et al (2016). *Cell Rep*

Physical Interactions with 783 interactions from BioGRID

Taipale-Lindquist-2014 0.01%

A quantitative chaperone interaction network reveals the architecture of cellular protein homeostasis pathways. Taipale et al (2014). *Cell*

Physical Interactions with 1,227 interactions from iRefIndex

Wang-Xiong-2019 0.01%

Impaired plasma membrane localization of ubiquitin ligase complex underlies 3-M syndrome development. Wang et al (2019). *J Clin Invest*

Physical Interactions with 609 interactions from BioGRID

Pichlmair-Superti-Furga-2012 0.01%

Viral immune modulators perturb the human molecular network by common and unique strategies. Pichlmair et al (2012). *Nature*

Physical Interactions with 14 interactions from BioGRID

Miyamoto-Sato-Yanagawa-2010 0.01%

A comprehensive resource of interacting protein regions for refining human transcription factor networks. Miyamoto-Sato et al (2010). *PLoS One*

Physical Interactions with 934 interactions from iRefIndex

Kawahara-Paes Leme-2017 0.01%

Mass spectrometry-based proteomics revealed Glypican-1 as a novel ADAM17 substrate. Kawahara et al (2017). *J Proteomics*

Physical Interactions with 114 interactions from BioGRID

Raman-Harper-2015 0.01%

Systematic proteomics of the VCP-UBXD adaptor network identifies a role for UBXN10 in regulating ciliogenesis. Raman et al (2015). *Nat Cell Biol*

Physical Interactions with 271 interactions from BioGRID

Kumar-Maddika-2017 0.01%

A Human Tyrosine Phosphatase Interactome Mapped by Proteomic Profiling. Kumar et al (2017). *J Proteome Res*

Physical Interactions with 1,863 interactions from BioGRID

Ravasi-Hayashizaki-2010 0.01%

An atlas of combinatorial transcriptional regulation in mouse and man. Ravasi et al (2010). *Cell*

Physical Interactions with 658 interactions from BioGRID

Li-Wang-2016 0.01%

Defining the Protein-Protein Interaction Network of the Human Protein Tyrosine Phosphatase Family. Li et al (2016). *Mol Cell Proteomics*

Physical Interactions with 1,476 interactions from BioGRID

Blandin-Richard-2013 0.01%

A human skeletal muscle interactome centered on proteins involved in muscular dystrophies: LGMD interactome. Blandin et al (2013). *Skelet Muscle*

Physical Interactions with 655 interactions from iRefIndex

Fragoza-Yu-2019 0.00%

Physical Interactions 77.64%

Fragoza-Yu-2019

Extensive disruption of protein interactions by genetic variants across the allele frequency spectrum in human populations. Fragoza et al (2019). *Nat Commun*

Physical Interactions with 603 interactions from iRefIndex

Liu-Sun-2019 0.00%

CCT3 acts upstream of YAP and TFCP2 as a potential target and tumour biomarker in liver cancer. Liu et al (2019). *Cell Death Dis*

Physical Interactions with 561 interactions from BioGRID

Ouyang-Gill-2009 0.00%

Direct binding of CoREST1 to SUMO-2/3 contributes to gene-specific repression by the LSD1/CoREST1/HDAC complex. Ouyang et al (2009). *Mol Cell*

Physical Interactions with 105 interactions from BioGRID

Albers-Koegl-2005 0.00%

Automated yeast two-hybrid screening for nuclear receptor-interacting proteins. Albers et al (2005). *Mol Cell Proteomics*

Physical Interactions with 289 interactions from iRefIndex

Swayampakula-Dedhar-2017 0.00%

The interactome of metabolic enzyme carbonic anhydrase IX reveals novel roles in tumor cell migration and invadopodia/MMP14-mediated invasion. Swayampakula et al (2017). *Oncogene*

Physical Interactions with 126 interactions from iRefIndex

Sahni-Vidal-2015 0.00%

Widespread macromolecular interaction perturbations in human genetic disorders. Sahni et al (2015). *Cell*

Physical Interactions with 591 interactions from BioGRID

IREF-biogrid 0.00%

Physical Interactions with 176,314 interactions from iRefIndex

Cao-Chinnaiyan-2014 0.00%

The central role of EED in the orchestration of polycomb group complexes. Cao et al (2014). *Nat Commun*

Physical Interactions with 1,769 interactions from BioGRID

Co-expression 8.01%

Wang-Maris-2006 0.60%

Integrative genomics identifies distinct molecular classes of neuroblastoma and shows that multiple genes are targeted by regional alterations in DNA copy number. Wang et al (2006). *Cancer Res*

Co-expression with 270,388 interactions from GEO

Mallon-McKay-2013 0.51%

StemCellDB: the human pluripotent stem cell database at the National Institutes of Health. Mallon et al (2013). *Stem Cell Res*

Co-expression with 602,113 interactions from GEO

Roth-Zlotnik-2006 0.50%

Gene expression analyses reveal molecular relationships among 20 regions of the human CNS. Roth et al (2006). *Neurogenetics*

Co-expression with 683,844 interactions from GEO

Ramaswamy-Golub-2001 0.48%

Multiclass cancer diagnosis using tumor gene expression signatures. Ramaswamy et al (2001). *Proc Natl Acad Sci U S A*

Co-expression 8.01%

Ramaswamy-Golub-2001

Co-expression with 284,829 interactions from supplementary material

Innocenti-Brown-2011 0.46%

Identification, replication, and functional fine-mapping of expression quantitative trait loci in primary human liver tissue. Innocenti et al (2011). *PLoS Genet*

Co-expression with 620,205 interactions from GEO

Alizadeh-Staudt-2000 0.46%

Distinct types of diffuse large B-cell lymphoma identified by gene expression profiling. Alizadeh et al (2000). *Nature*

Co-expression with 92,360 interactions from supplementary material

Dobbin-Giordano-2005 0.46%

Interlaboratory comparability study of cancer gene expression analysis using oligonucleotide microarrays. Dobbin et al (2005). *Clin Cancer Res*

Co-expression with 452,322 interactions from GEO

Rieger-Chu-2004 0.45%

Toxicity from radiation therapy associated with abnormal transcriptional responses to DNA damage. Rieger et al (2004). *Proc Natl Acad Sci U S A*

Co-expression with 266,879 interactions from GEO

Bild-Nevins-2006 B 0.44%

Oncogenic pathway signatures in human cancers as a guide to targeted therapies. Bild et al (2006). *Nature*

Co-expression with 285,368 interactions from GEO

Burington-Shaughnessy-2008 0.44%

Tumor cell gene expression changes following short-term in vivo exposure to single agent chemotherapeutics are related to survival in multiple myeloma. Burington et al (2008). *Clin Cancer Res*

Co-expression with 295,320 interactions from GEO

Boldrick-Relman-2002 0.39%

Stereotyped and specific gene expression programs in human innate immune responses to bacteria. Boldrick et al (2002). *Proc Natl Acad Sci U S A*

Co-expression with 116,197 interactions from supplementary material

Arijs-Rutgeerts-2009 0.39%

Mucosal gene expression of antimicrobial peptides in inflammatory bowel disease before and after first infliximab treatment. Arijs et al (2009). *PLoS One*

Co-expression with 676,695 interactions from GEO

Jiang-de Kok-2017 0.36%

Omics-based identification of the combined effects of idiosyncratic drugs and inflammatory cytokines on the development of drug-induced liver injury. Jiang et al (2017). *Toxicol Appl Pharmacol*

Co-expression with 444,959 interactions from GEO

Perou-Botstein-2000 0.36%

Molecular portraits of human breast tumours. Perou et al (2000). *Nature*

Co-expression with 189,373 interactions from supplementary material

Chen-Brown-2002 0.35%

Gene expression patterns in human liver cancers. Chen et al (2002). *Mol Biol Cell*

Co-expression 8.01%

Chen-Brown-2002

Co-expression with 291,300 interactions from supplementary material

Wang-Cheung-2015 0.34%

Genetic variation in insulin-induced kinase signaling. Wang et al (2015). *Mol Syst Biol*

Co-expression with 422,896 interactions from GEO

Wu-Garvey-2007 0.28%

The effect of insulin on expression of genes and biochemical pathways in human skeletal muscle. Wu et al (2007). *Endocrine*

Co-expression with 275,155 interactions from GEO

Rosenwald-Staudt-2001 0.28%

Relation of gene expression phenotype to immunoglobulin mutation genotype in B cell chronic lymphocytic leukemia. Rosenwald et al (2001). *J Exp Med*

Co-expression with 118,097 interactions from supplementary material

Ross-Perou-2001 0.26%

A comparison of gene expression signatures from breast tumors and breast tissue derived cell lines. Ross et al (2001). *Dis Markers*

Co-expression with 146,858 interactions from supplementary material

Perou-Botstein-1999 0.22%

Distinctive gene expression patterns in human mammary epithelial cells and breast cancers. Perou et al (1999). *Proc Natl Acad Sci U S A*

Co-expression with 68,200 interactions from supplementary material

Predicted 5.37%

I2D-Li-Vidal-2004-CE-DATA-Worm2Human 0.73%

A map of the interactome network of the metazoan C. elegans. Li et al (2004). *Science*

Predicted with 48 interactions from I2D

I2D-BioGRID-Yeast2Human 0.71%

BioGRID: a general repository for interaction datasets. Stark et al (2006). *Nucleic Acids Res*

Predicted with 17,314 interactions from I2D

I2D-vonMering-Bork-2002-High-Yeast2Human 0.60%

Comparative assessment of large-scale data sets of protein-protein interactions. von Mering et al (2002). *Nature*

Predicted with 723 interactions from I2D

I2D-Li-Vidal-2004-interolog-Worm2Human 0.51%

A map of the interactome network of the metazoan C. elegans. Li et al (2004). *Science*

Predicted with 396 interactions from I2D

I2D-vonMering-Bork-2002-Medium-Yeast2Human 0.40%

Comparative assessment of large-scale data sets of protein-protein interactions. von Mering et al (2002). *Nature*

Predicted with 1,280 interactions from I2D

I2D-Tarassov-PCA-Yeast2Human 0.29%

An in vivo map of the yeast protein interactome. Tarassov et al (2008). *Science*

Predicted with 235 interactions from I2D

Wu-Stein-2010 0.24%

Predicted 5.37%

Wu-Stein-2010

A human functional protein interaction network and its application to cancer data analysis. Wu et al (2010). *Genome Biol*

Predicted with 89,967 interactions from supplementary material

I2D-vonMering-Bork-2002-Low-Yeast2Human 0.18%

Comparative assessment of large-scale data sets of protein-protein interactions. von Mering et al (2002). *Nature*

Predicted with 7,979 interactions from I2D

I2D-Wang-Orkin-2006-EScmplxIP-Mouse2Human 0.18%

A protein interaction network for pluripotency of embryonic stem cells. Wang et al (2006). *Nature*

Predicted with 5 interactions from I2D

I2D-IntAct-Mouse2Human 0.16%

The IntAct molecular interaction database in 2010. Aranda et al (2010). *Nucleic Acids Res*

Predicted with 11,478 interactions from I2D

I2D-INNATEDB-Mouse2Human 0.15%

InnateDB: facilitating systems-level analyses of the mammalian innate immune response. Lynn et al (2008). *Mol Syst Biol*

Predicted with 4,049 interactions from I2D

I2D-BioGRID-Rat2Human 0.14%

BioGRID: a general repository for interaction datasets. Stark et al (2006). *Nucleic Acids Res*

Predicted with 2,148 interactions from I2D

I2D-BioGRID-Mouse2Human 0.14%

BioGRID: a general repository for interaction datasets. Stark et al (2006). *Nucleic Acids Res*

Predicted with 10,524 interactions from I2D

Stuart-Kim-2003 0.14%

A gene-coexpression network for global discovery of conserved genetic modules. Stuart et al (2003). *Science*

Predicted with 25,001 interactions from supplementary material

I2D-Yu-Vidal-2008-GoldStd-Yeast2Human 0.12%

High-quality binary protein interaction map of the yeast interactome network. Yu et al (2008). *Science*

Predicted with 173 interactions from I2D

I2D-Krogan-Greenblatt-2006-Core-Yeast2Human 0.10%

Global landscape of protein complexes in the yeast Saccharomyces cerevisiae. Krogan et al (2006). *Nature*

Predicted with 860 interactions from I2D

I2D-BioGRID-Fly2Human 0.09%

BioGRID: a general repository for interaction datasets. Stark et al (2006). *Nucleic Acids Res*

Predicted with 8,676 interactions from I2D

I2D-BIND-Rat2Human 0.08%

BIND--a data specification for storing and describing biomolecular interactions, molecular complexes and pathways. Bader et al (2000). *Bioinformatics*

Predicted with 468 interactions from I2D

I2D-IntAct-Yeast2Human 0.08%

The IntAct molecular interaction database in 2010. Aranda et al (2010). *Nucleic Acids Res*

Predicted 5.37%

I2D-IntAct-Yeast2Human

Predicted with 7,325 interactions from I2D

I2D-IntAct-Rat2Human 0.08%

The IntAct molecular interaction database in 2010. Aranda et al (2010). *Nucleic Acids Res*

Predicted with 1,690 interactions from I2D

I2D-Chen-Pawson-2009-PiwiScreen-Mouse2Human 0.06%

Mouse Piwi interactome identifies binding mechanism of Tdrkh Tudor domain to arginine methylated Miwi. Chen et al (2009). *Proc Natl Acad Sci U S A*

Predicted with 29 interactions from I2D

I2D-BIND-Mouse2Human 0.05%

BIND--a data specification for storing and describing biomolecular interactions, molecular complexes and pathways. Bader et al (2000). *Bioinformatics*

Predicted with 1,007 interactions from I2D

I2D-Krogan-Greenblatt-2006-NonCore-Yeast2Human 0.04%

Global landscape of protein complexes in the yeast Saccharomyces cerevisiae. Krogan et al (2006). *Nature*

Predicted with 678 interactions from I2D

I2D-MGI-Mouse2Human 0.04%

Ontological visualization of protein-protein interactions. Drabkin et al (2005). *BMC Bioinformatics*

Predicted with 595 interactions from I2D

I2D-MINT-Rat2Human 0.04%

MINT: a Molecular INTeraction database. Zanzoni et al (2002). *FEBS Lett*

Predicted with 540 interactions from I2D

I2D-BIND-Worm2Human 0.02%

BIND--a data specification for storing and describing biomolecular interactions, molecular complexes and pathways. Bader et al (2000). *Bioinformatics*

Predicted with 349 interactions from I2D

I2D-BIND-Yeast2Human 0.01%

BIND--a data specification for storing and describing biomolecular interactions, molecular complexes and pathways. Bader et al (2000). *Bioinformatics*

Predicted with 599 interactions from I2D

I2D-Formstecher-Daviet-2005-Embryo-Fly2Human 0.00%

Protein interaction mapping: a Drosophila case study. Formstecher et al (2005). *Genome Res*

Predicted with 270 interactions from I2D

Co-localization 3.63%

Zhang-Shang-2006 1.44%

The catalytic subunit of the proteasome is engaged in the entire process of estrogen receptor-regulated transcription. Zhang et al (2006). *EMBO J*

Co-localization with 53 interactions from BioGRID

Schadt-Shoemaker-2004 1.11%

A comprehensive transcript index of the human genome generated using microarrays and computational approaches. Schadt et al (2004). *Genome Biol*

Co-localization 3.63%

Schadt-Shoemaker-2004

Co-localization with 59,920 interactions from GEO

Johnson-Shoemaker-2003 0.71%

Genome-wide survey of human alternative pre-mRNA splicing with exon junction microarrays. Johnson et al (2003). *Science*

Co-localization with 426,464 interactions from GEO

Chen-Huang-2014 0.37%

Using an in situ proximity ligation assay to systematically profile endogenous protein-protein interactions in a pathway network. Chen et al (2014). *J Proteome Res*

Co-localization with 559 interactions from BioGRID

Genetic Interactions 2.87%

Shen-Mali-2017 0.32%

Combinatorial CRISPR-Cas9 screens for de novo mapping of genetic interactions. Shen et al (2017). *Nat Methods*

Genetic Interactions with 152 interactions from BioGRID

Toyoshima-Grandori-2012 0.27%

Functional genomics identifies therapeutic targets for MYC-driven cancer. Toyoshima et al (2012). *Proc Natl Acad Sci U S A*

Genetic Interactions with 101 interactions from BioGRID

Matsuoka-Elledge-2007 0.25%

ATM and ATR substrate analysis reveals extensive protein networks responsive to DNA damage. Matsuoka et al (2007). *Science*

Genetic Interactions with 342 interactions from iRefIndex

Vizeacoumar-Moffat-2013 0.25%

A negative genetic interaction map in isogenic cancer cell lines reveals cancer cell vulnerabilities. Vizeacoumar et al (2013). *Mol Syst Biol*

Genetic Interactions with 201 interactions from BioGRID

Xie-Green-2012 0.23%

A synthetic interaction screen identifies factors selectively required for proliferation and TERT transcription in p53-deficient human cancer cells. Xie et al (2012). *PLoS Genet*

Genetic Interactions with 100 interactions from BioGRID

Srivas-Ideker-2016 0.23%

A Network of Conserved Synthetic Lethal Interactions for Exploration of Precision Cancer Therapy. Srivas et al (2016). *Mol Cell*

Genetic Interactions with 173 interactions from BioGRID

BIOGRID-SMALL-SCALE-STUDIES 0.22%

Genetic Interactions with 651 interactions from BioGRID

IREF-SMALL-SCALE-STUDIES 0.22%

Genetic Interactions with 1,159 interactions from iRefIndex

Han-Bassik-2017 A 0.22%

Synergistic drug combinations for cancer identified in a CRISPR screen for pairwise genetic interactions. Han et al (2017). *Nat Biotechnol*

Genetic Interactions with 1,042 interactions from BioGRID

Du-Krogan-2017 0.11%

Genetic Interactions 2.87%

Du-Krogan-2017

Genetic interaction mapping in mammalian cells using CRISPR interference. Du et al (2017). *Nat Methods*

Genetic Interactions with 166 interactions from BioGRID

Horlbeck-Gilbert-2018 A 0.11%

Mapping the Genetic Landscape of Human Cells. Horlbeck et al (2018). *Cell*

Genetic Interactions with 1,808 interactions from BioGRID

Horlbeck-Gilbert-2018 B 0.10%

Mapping the Genetic Landscape of Human Cells. Horlbeck et al (2018). *Cell*

Genetic Interactions with 946 interactions from BioGRID

Blomen-Brummelkamp-2015 0.09%

Gene essentiality and synthetic lethality in haploid human cells. Blomen et al (2015). *Science*

Genetic Interactions with 127 interactions from BioGRID

Han-Bassik-2017 B 0.07%

Synergistic drug combinations for cancer identified in a CRISPR screen for pairwise genetic interactions. Han et al (2017). *Nat Biotechnol*

Genetic Interactions with 893 interactions from BioGRID

Willingham-Muchowski-2003 0.06%

Yeast genes that enhance the toxicity of a mutant huntingtin fragment or alpha-synuclein. Willingham et al (2003). *Science*

Genetic Interactions with 37 interactions from BioGRID

Xiao-Brown-2018 0.06%

Estrogen-regulated feedback loop limits the efficacy of estrogen receptor-targeted breast cancer therapy. Xiao et al (2018). *Proc Natl Acad Sci U S A*

Genetic Interactions with 684 interactions from BioGRID

Achuthankutty-Mailand-2019 0.03%

Regulation of ETAA1-mediated ATR activation couples DNA replication fidelity and genome stability. Achuthankutty et al (2019). *J Cell Biol*

Genetic Interactions with 99 interactions from BioGRID

Luo-Elledge-2009 0.03%

A genome-wide RNAi screen identifies multiple synthetic lethal interactions with the Ras oncogene. Luo et al (2009). *Cell*

Genetic Interactions with 316 interactions from BioGRID

Lin-Smith-2010 0.01%

A genome-wide map of human genetic interactions inferred from radiation hybrid genotypes. Lin et al (2010). *Genome Res*

Genetic Interactions with 4,805,334 interactions from supplementary material

Pathway 1.88%

Wu-Stein-2010 0.60%

A human functional protein interaction network and its application to cancer data analysis. Wu et al (2010). *Genome Biol*

Pathway with 78,117 interactions from supplementary material

NCI_NATURE 0.39%

Pathway with 10,118 interactions from Pathway Commons

**Pathway**	1.88%
REACTOME<br>Pathway with 24,890 interactions from Pathway Commons	0.38%
IMID<br>Pathway with 1,023 interactions from Pathway Commons	0.27%
CELL_MAP<br>Pathway with 397 interactions from Pathway Commons	0.23%
HUMANCYC<br>Pathway with 681 interactions from Pathway Commons	0.01%

**Shared protein domains**	0.60%
INTERPRO<br>Shared protein domains with 621,159 interactions from InterPro	0.39%
PFAM<br>Shared protein domains with 471,533 interactions from Pfam	0.21%
