# Supplementary material for: Downregulation of dermatopontin in cholangiocarcinoma cells suppresses CCL19 secretion of macrophages and immune infiltration
Source: J Cancer Res Clin Oncol. 2024 Feb 1;150(2):66. doi: 10.1007/s00432-023-05532-1 (PMC10834663; doi:10.1007/s00432-023-05532-1)
Supplement: Supplementary file 1 — Supplementary file1 (ZIP 153266 KB) [file 432_2023_5532_MOESM1_ESM.zip › Websites/UALCAN/肝癌分期.pdf]

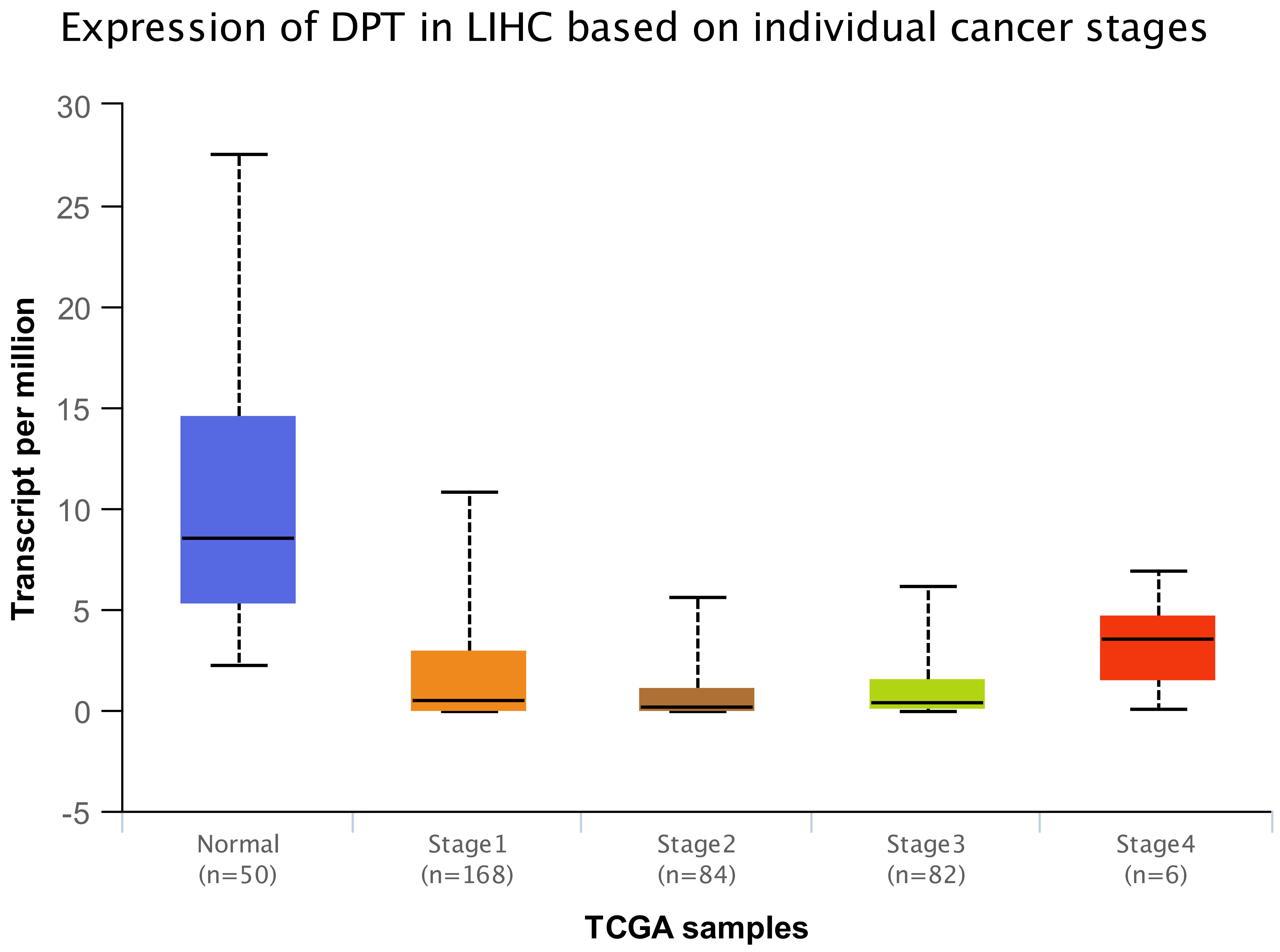
Expression of DPT in LIHC based on individual cancer stages
Transcript per million
30
25
20
15
10
5
0
-5
Normal
(n=50)
Stage1
(n=168)
Stage2
(n=84)
Stage3
(n=82)
Stage4
(n=6)
TCGA samples
