# Supplementary material for: Downregulation of dermatopontin in cholangiocarcinoma cells suppresses CCL19 secretion of macrophages and immune infiltration
Source: J Cancer Res Clin Oncol. 2024 Feb 1;150(2):66. doi: 10.1007/s00432-023-05532-1 (PMC10834663; doi:10.1007/s00432-023-05532-1)
Supplement: Supplementary file 1 — Supplementary file1 (ZIP 153266 KB) [file 432_2023_5532_MOESM1_ESM.zip › Websites/UALCAN/肿瘤分期.pdf]

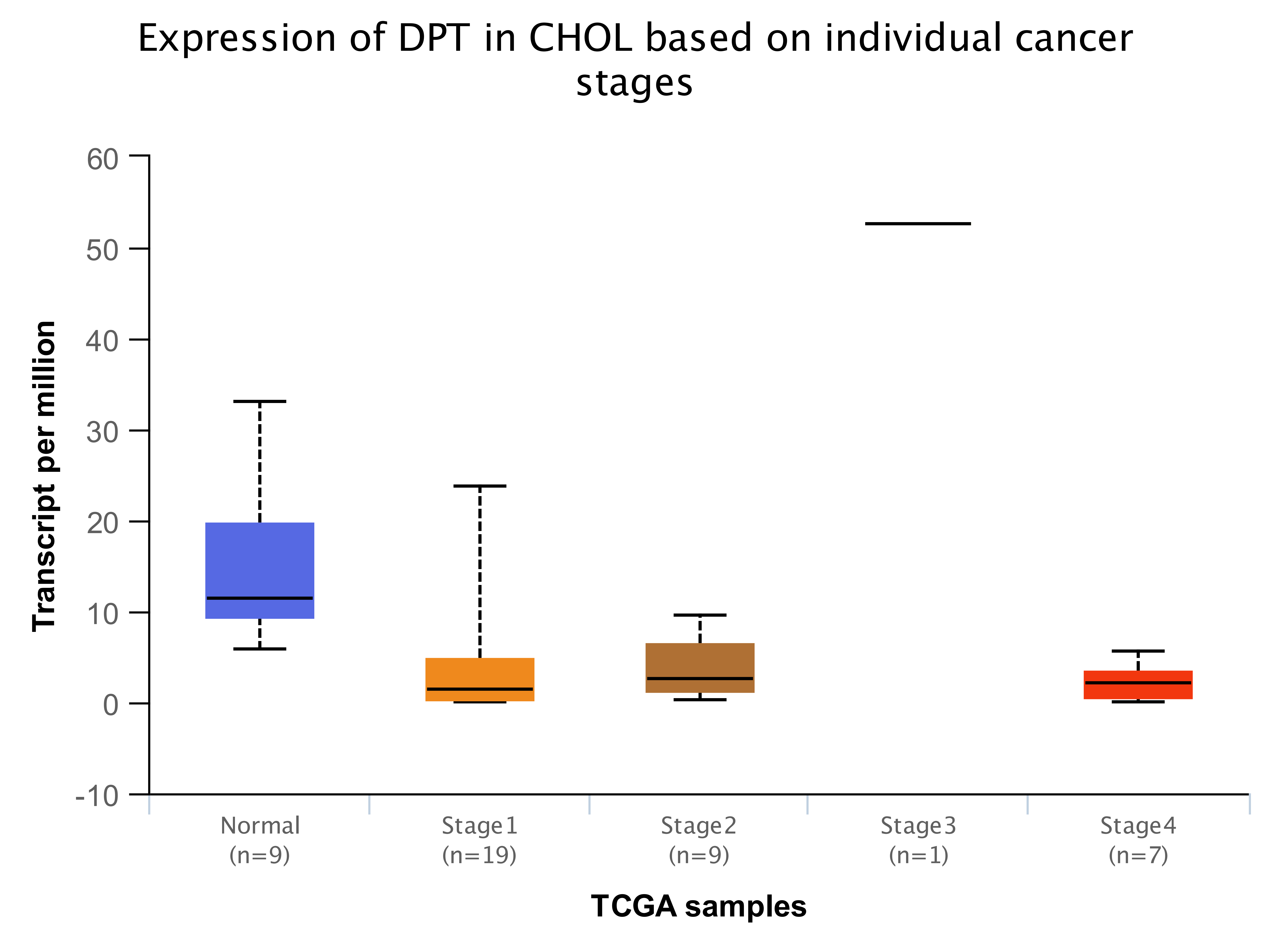
Expression of DPT in CHOL based on individual cancer stages
Transcript per million
60
50
40
30
20
10
0
-10
Normal (n=9)
Stage1 (n=19)
Stage2 (n=9)
Stage3 (n=1)
Stage4 (n=7)
TCGA samples
