# Supplementary material for: Downregulation of dermatopontin in cholangiocarcinoma cells suppresses CCL19 secretion of macrophages and immune infiltration
Source: J Cancer Res Clin Oncol. 2024 Feb 1;150(2):66. doi: 10.1007/s00432-023-05532-1 (PMC10834663; doi:10.1007/s00432-023-05532-1)
Supplement: Supplementary file 1 — Supplementary file1 (ZIP 153266 KB) [file 432_2023_5532_MOESM1_ESM.zip › Websites/UALCAN/肿瘤分级.pdf]

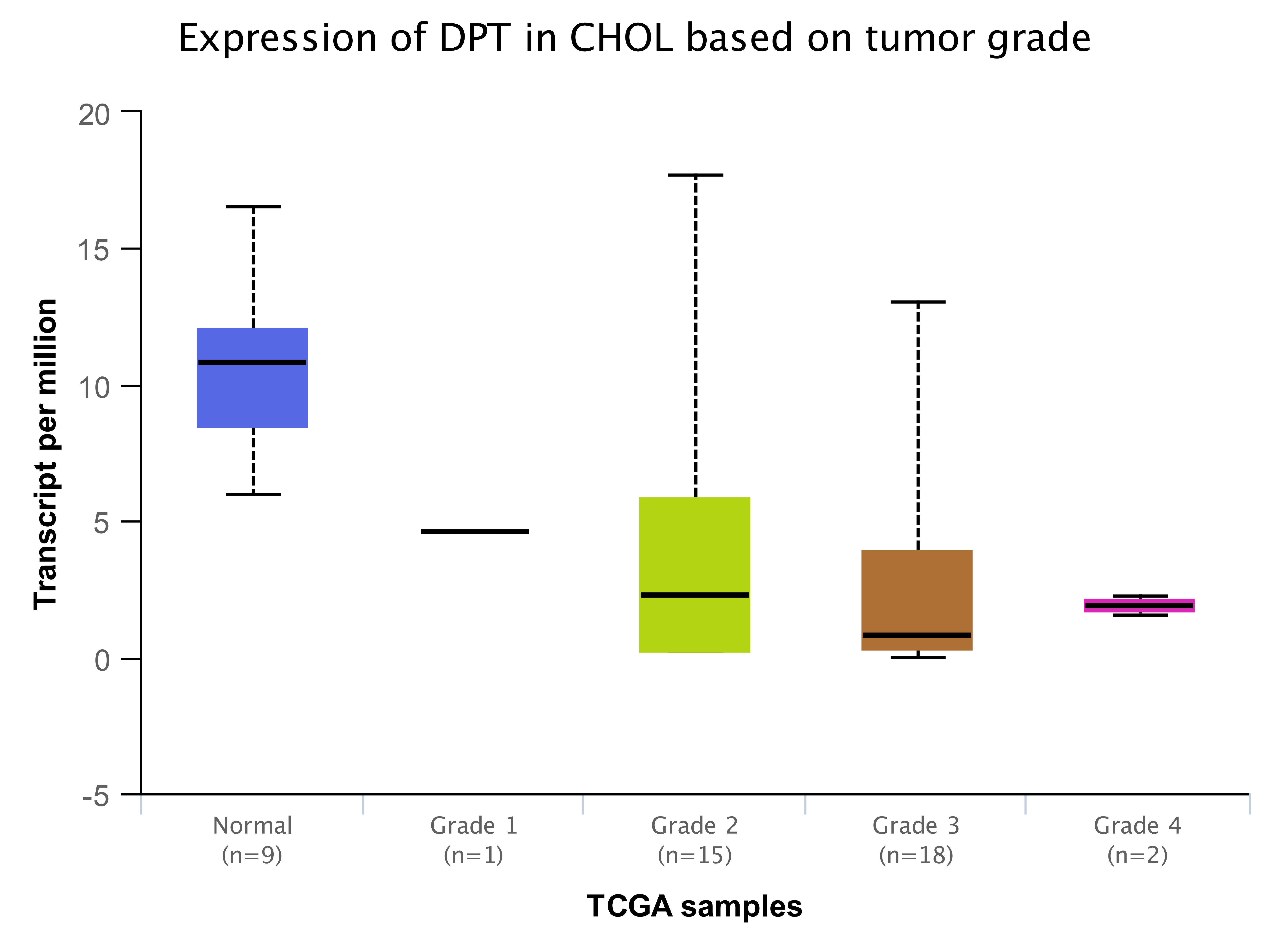

Expression of DPT in CHOL based on tumor grade
Transcript per million
20
15
10
5
0
-5
Normal (n=9)
Grade 1 (n=1)
Grade 2 (n=15)
Grade 3 (n=18)
Grade 4 (n=2)
TCGA samples
