# Supplementary material for: Downregulation of dermatopontin in cholangiocarcinoma cells suppresses CCL19 secretion of macrophages and immune infiltration
Source: J Cancer Res Clin Oncol. 2024 Feb 1;150(2):66. doi: 10.1007/s00432-023-05532-1 (PMC10834663; doi:10.1007/s00432-023-05532-1)
Supplement: Supplementary file 1 — Supplementary file1 (ZIP 153266 KB) [file 432_2023_5532_MOESM1_ESM.zip › Websites/cbioportal/DPT突变的分组/oncoprint.pdf]

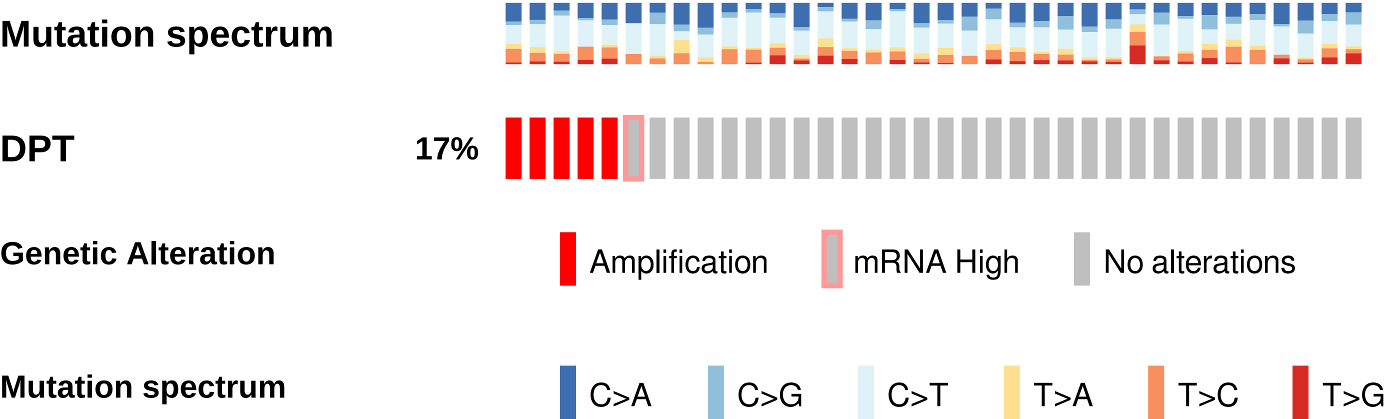
Mutation spectrum
DPT
17%
Genetic Alteration
Amplification
mRNA High
No alterations
Mutation spectrum
C>A
C>G
C>T
T>A
T>C
T>G
