# Supplementary material for: Downregulation of dermatopontin in cholangiocarcinoma cells suppresses CCL19 secretion of macrophages and immune infiltration
Source: J Cancer Res Clin Oncol. 2024 Feb 1;150(2):66. doi: 10.1007/s00432-023-05532-1 (PMC10834663; doi:10.1007/s00432-023-05532-1)
Supplement: Supplementary file 1 — Supplementary file1 (ZIP 153266 KB) [file 432_2023_5532_MOESM1_ESM.zip › Websites/cbioportal/co-expression/CCL19.pdf]

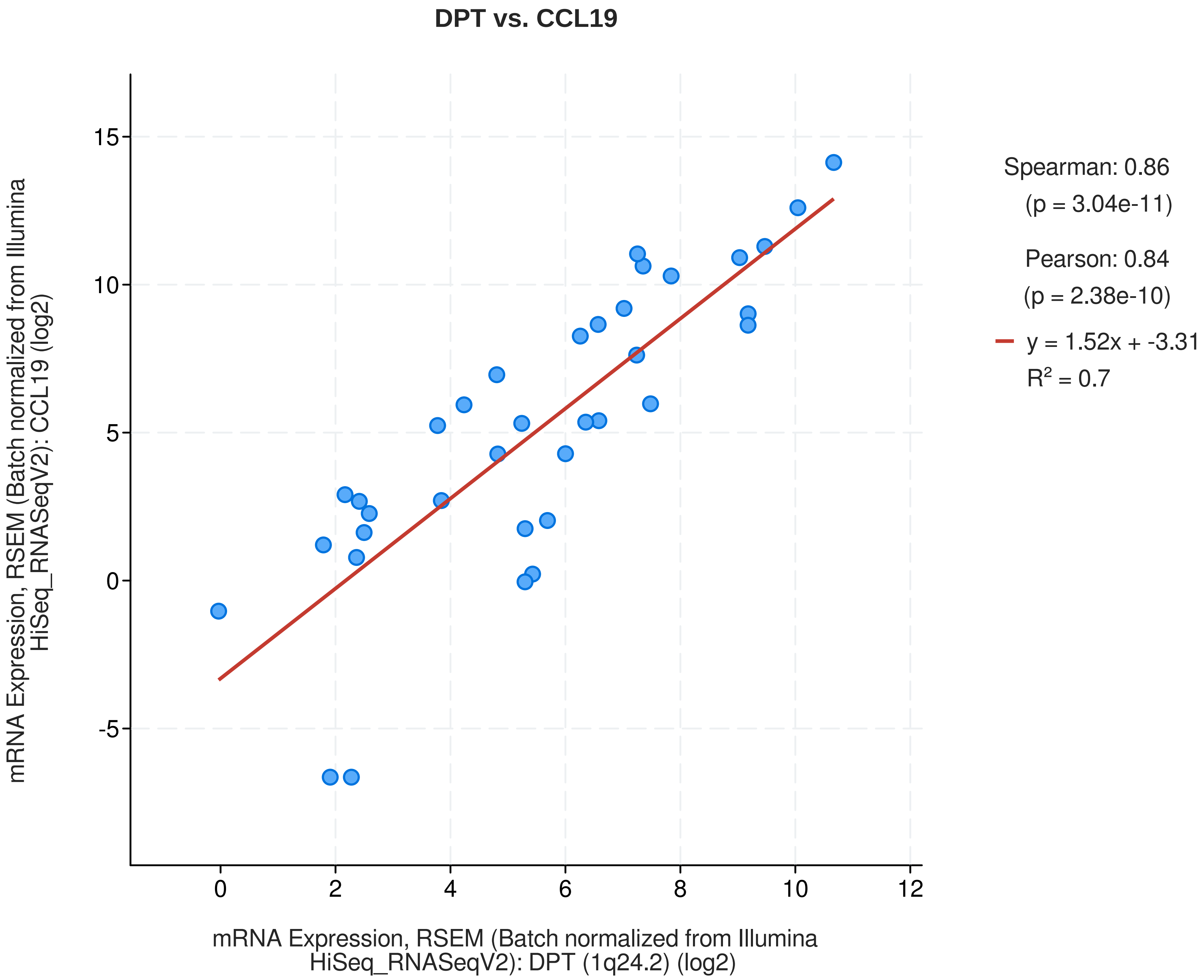
DPT vs. CCL19
mRNA Expression, RSEM (Batch normalized from Illumina HiSeq_RNASeqV2): CCL19 (log2)
15
10
5
0
-5
0
2
4
6
8
10
12
mRNA Expression, RSEM (Batch normalized from Illumina HiSeq_RNASeqV2): DPT (1q24.2) (log2)
Spearman: 0.86
(p = 3.04e-11)
Pearson: 0.84
(p = 2.38e-10)
y = 1.52x + -3.31
R² = 0.7
