# Supplementary material for: Downregulation of dermatopontin in cholangiocarcinoma cells suppresses CCL19 secretion of macrophages and immune infiltration
Source: J Cancer Res Clin Oncol. 2024 Feb 1;150(2):66. doi: 10.1007/s00432-023-05532-1 (PMC10834663; doi:10.1007/s00432-023-05532-1)
Supplement: Supplementary file 1 — Supplementary file1 (ZIP 153266 KB) [file 432_2023_5532_MOESM1_ESM.zip › Websites/cbioportal/co-expression/DPT共表达前十基因.docx]

| Correlated Gene | Cytoband | Spearman's Correlation | | p-Value | q-Value |
| --- | --- | --- | --- | --- | --- |
| MFAP4 | 17p11.2 | 0.873 | 3.93E-12 | | 7.65E-08 |
| ACKR1 | 1q23.2 | 0.856 | 2.89E-11 | | 1.97E-07 |
| CCL19 | 9p13.3 | 0.855 | 3.04E-11 | | 1.97E-07 |
| C7 | 5p13.1 | 0.842 | 1.20E-10 | | 5.86E-07 |
| BHLHE22 | 8q12.3 | 0.823 | 7.37E-10 | | 2.87E-06 |
| LHX6 | 9q33.2 | 0.815 | 1.45E-09 | | 4.7E-06 |
| MEOX1 | 17q21.31 | 0.809 | 2.41E-09 | | 6.69E-06 |
| SELP | 1q24.2 | 0.798 | 5.50E-09 | | 1.34E-05 |
| TMEM119 | 12q23.3 | 0.792 | 8.56E-09 | | 1.85E-05 |
| SRL | 16p13.3 | 0.778 | 2.37E-08 | | 4.62E-05 |
