# Supplementary material for: Downregulation of dermatopontin in cholangiocarcinoma cells suppresses CCL19 secretion of macrophages and immune infiltration
Source: J Cancer Res Clin Oncol. 2024 Feb 1;150(2):66. doi: 10.1007/s00432-023-05532-1 (PMC10834663; doi:10.1007/s00432-023-05532-1)
Supplement: Supplementary file 1 — Supplementary file1 (ZIP 153266 KB) [file 432_2023_5532_MOESM1_ESM.zip › Websites/cbioportal/co-expression/SELP.pdf]

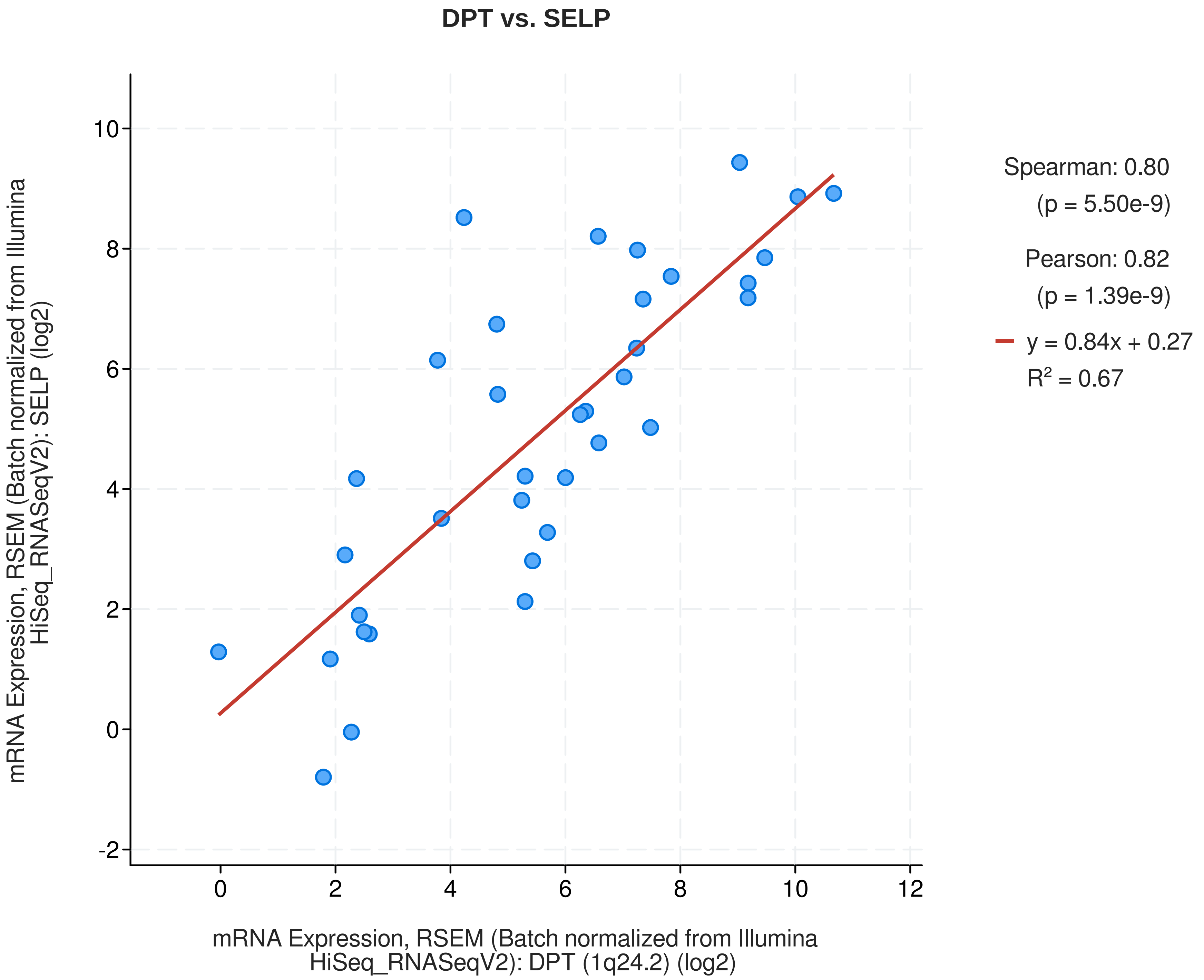
DPT vs. SELP
Spearman: 0.80
(p = 5.50e-9)
Pearson: 0.82
(p = 1.39e-9)
y = 0.84x + 0.27
$R^2$ = 0.67
mRNA Expression, RSEM (Batch normalized from Illumina HiSeq_RNASeqV2): SELP (log2)
mRNA Expression, RSEM (Batch normalized from Illumina HiSeq_RNASeqV2): DPT (1q24.2) (log2)
-2
0
2
4
6
8
10
12
