# Supplementary material for: Downregulation of dermatopontin in cholangiocarcinoma cells suppresses CCL19 secretion of macrophages and immune infiltration
Source: J Cancer Res Clin Oncol. 2024 Feb 1;150(2):66. doi: 10.1007/s00432-023-05532-1 (PMC10834663; doi:10.1007/s00432-023-05532-1)
Supplement: Supplementary file 1 — Supplementary file1 (ZIP 153266 KB) [file 432_2023_5532_MOESM1_ESM.zip › Websites/single cell/05-cluster.TSEN.pdf]

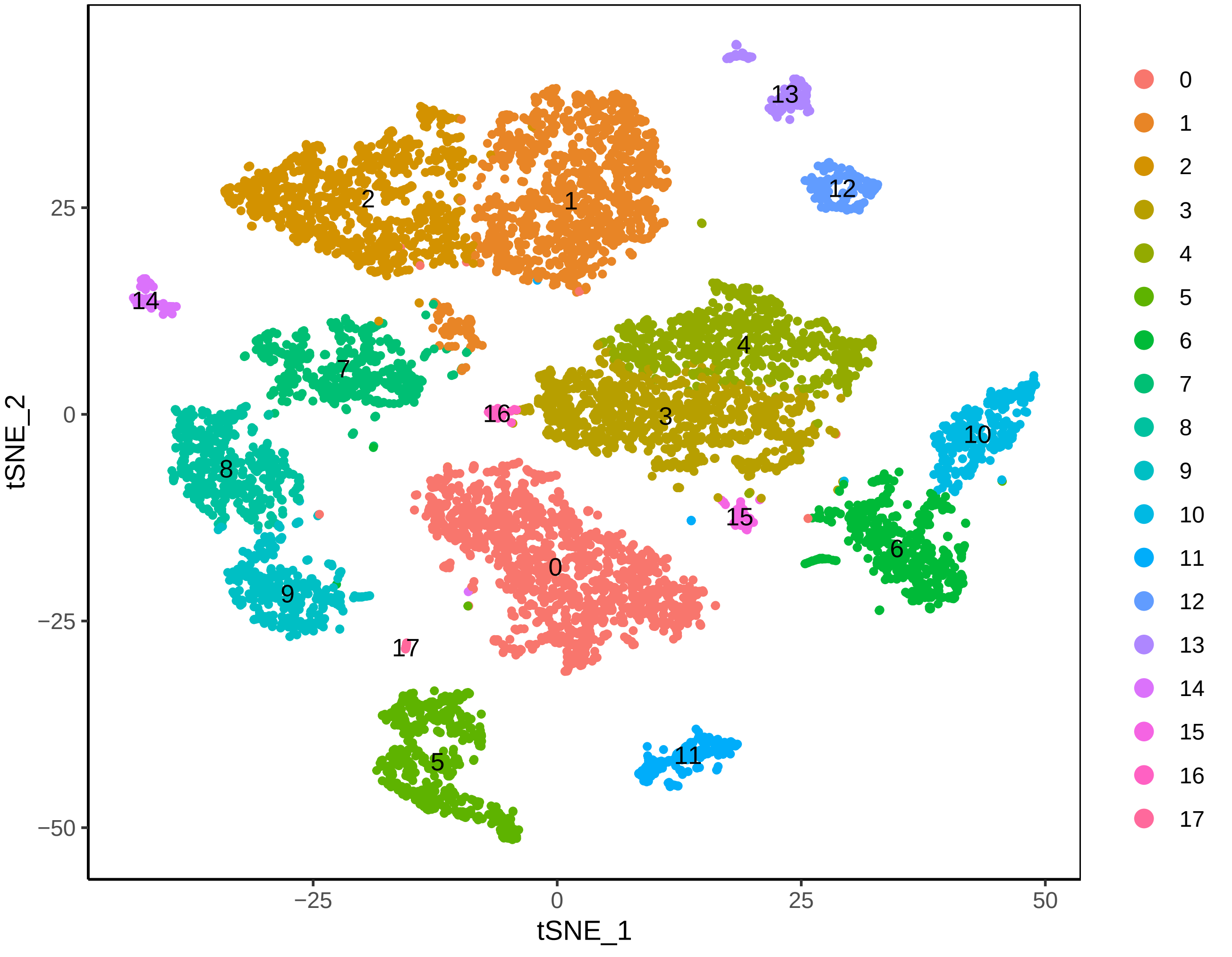

tSNE_2
tSNE_1
25
0
−25
−50
−25
0
25
50
0
1
2
3
4
5
6
7
8
9
10
11
12
13
14
15
16
17
