# Supplementary material for: Downregulation of dermatopontin in cholangiocarcinoma cells suppresses CCL19 secretion of macrophages and immune infiltration
Source: J Cancer Res Clin Oncol. 2024 Feb 1;150(2):66. doi: 10.1007/s00432-023-05532-1 (PMC10834663; doi:10.1007/s00432-023-05532-1)
Supplement: Supplementary file 1 — Supplementary file1 (ZIP 153266 KB) [file 432_2023_5532_MOESM1_ESM.zip › Websites/single cell/08-ann.scRNA.TSEN.pdf]

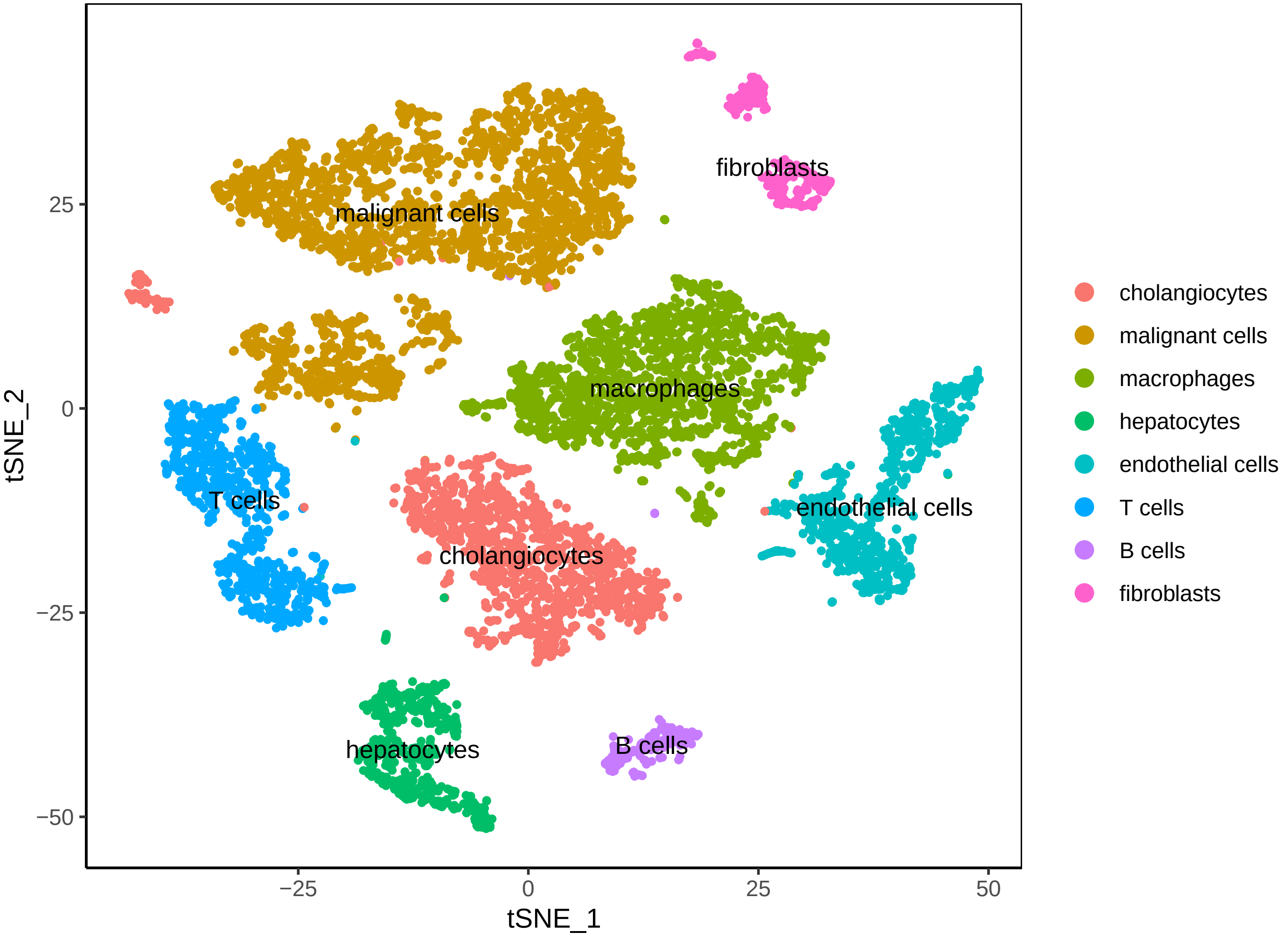
malignant cells
fibroblasts
macrophages
T cells
endothelial cells
cholangiocytes
hepatocytes
B cells
tSNE_2
tSNE_1
25
0
−25
−50
−25
0
25
50
cholangiocytes
malignant cells
macrophages
hepatocytes
endothelial cells
T cells
B cells
fibroblasts
