# Supplementary material for: Downregulation of dermatopontin in cholangiocarcinoma cells suppresses CCL19 secretion of macrophages and immune infiltration
Source: J Cancer Res Clin Oncol. 2024 Feb 1;150(2):66. doi: 10.1007/s00432-023-05532-1 (PMC10834663; doi:10.1007/s00432-023-05532-1)
Supplement: Supplementary file 1 — Supplementary file1 (ZIP 153266 KB) [file 432_2023_5532_MOESM1_ESM.zip › Websites/single cell/08.ann_cluster_marker.pdf]

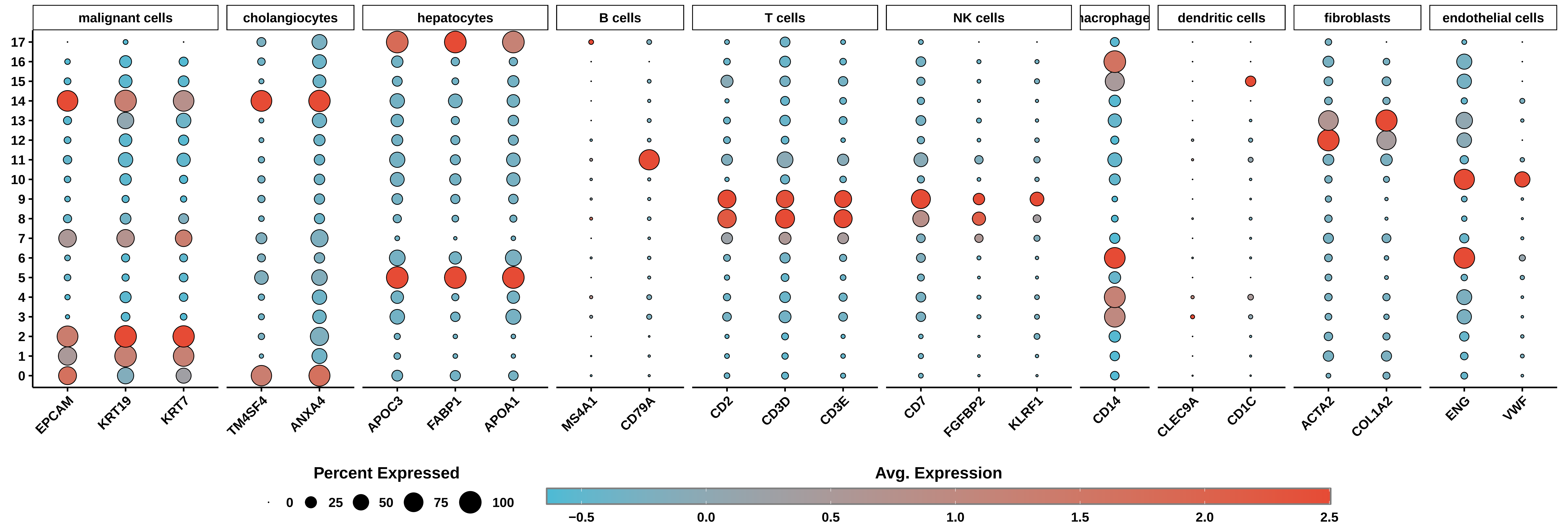

malignant cells
cholangiocytes
hepatocytes
B cells
T cells
NK cells
acrophage
dendritic cells
fibroblasts
endothelial cells
17
16
15
14
13
12
11
10
9
8
7
6
5
4
3
2
1
0
EPCAM
KRT19
KRT7
TM4SF4
ANXA4
APOC3
FABP1
APOA1
MS4A1
CD79A
CD2
CD3D
CD3E
CD7
FGFBP2
KLRF1
CD14
CLEC9A
CD1C
ACTA2
COL1A2
ENG
VWF
Percent Expressed
0
25
50
75
100
Avg. Expression
-0.5
0.0
0.5
1.0
1.5
2.0
2.5
