# Supplementary material for: Downregulation of dermatopontin in cholangiocarcinoma cells suppresses CCL19 secretion of macrophages and immune infiltration
Source: J Cancer Res Clin Oncol. 2024 Feb 1;150(2):66. doi: 10.1007/s00432-023-05532-1 (PMC10834663; doi:10.1007/s00432-023-05532-1)
Supplement: Supplementary file 1 — Supplementary file1 (ZIP 153266 KB) [file 432_2023_5532_MOESM1_ESM.zip › Websites/single cell/09-cell_marker.hetmap.pdf]

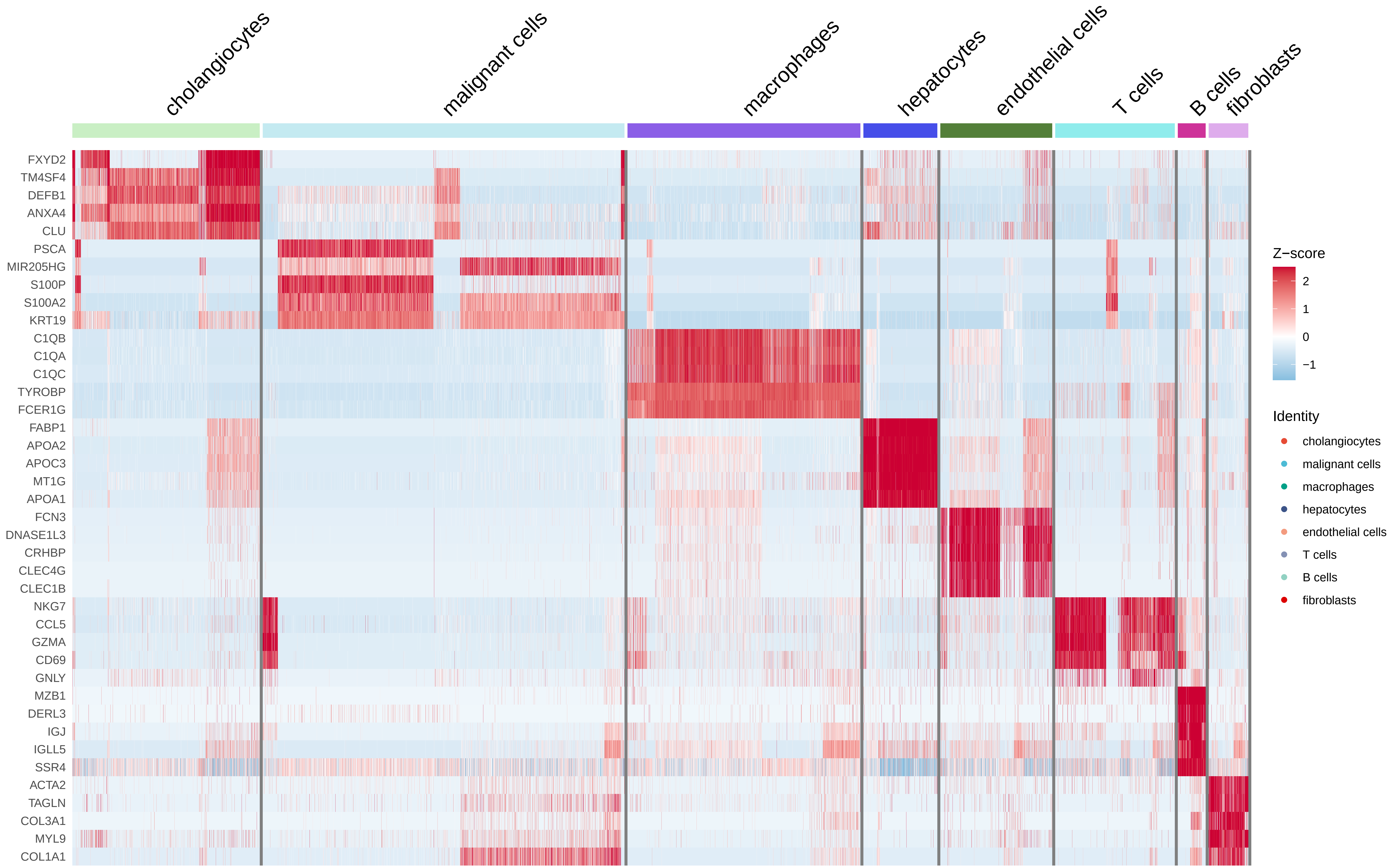
cholangiocytes
malignant cells
macrophages
hepatocytes
endothelial cells
T cells
B cells
fibroblasts
FXYD2
TM4SF4
DEFB1
ANXA4
CLU
PSCA
MIR205HG
S100P
S100A2
KRT19
C1QB
C1QA
C1QC
TYROBP
FCER1G
FABP1
APOA2
APOC3
MT1G
APOA1
FCN3
DNASE1L3
CRHBP
CLEC4G
CLEC1B
NKG7
CCL5
GZMA
CD69
GNLY
MZB1
DERL3
IGJ
IGLL5
SSR4
ACTA2
TAGLN
COL3A1
MYL9
COL1A1
Z−score
2
1
0
−1
Identity
cholangiocytes
malignant cells
macrophages
hepatocytes
endothelial cells
T cells
B cells
fibroblasts
