# Supplementary material for: Downregulation of dermatopontin in cholangiocarcinoma cells suppresses CCL19 secretion of macrophages and immune infiltration
Source: J Cancer Res Clin Oncol. 2024 Feb 1;150(2):66. doi: 10.1007/s00432-023-05532-1 (PMC10834663; doi:10.1007/s00432-023-05532-1)
Supplement: Supplementary file 1 — Supplementary file1 (ZIP 153266 KB) [file 432_2023_5532_MOESM1_ESM.zip › Websites/single cell/CCL19表达.pdf]

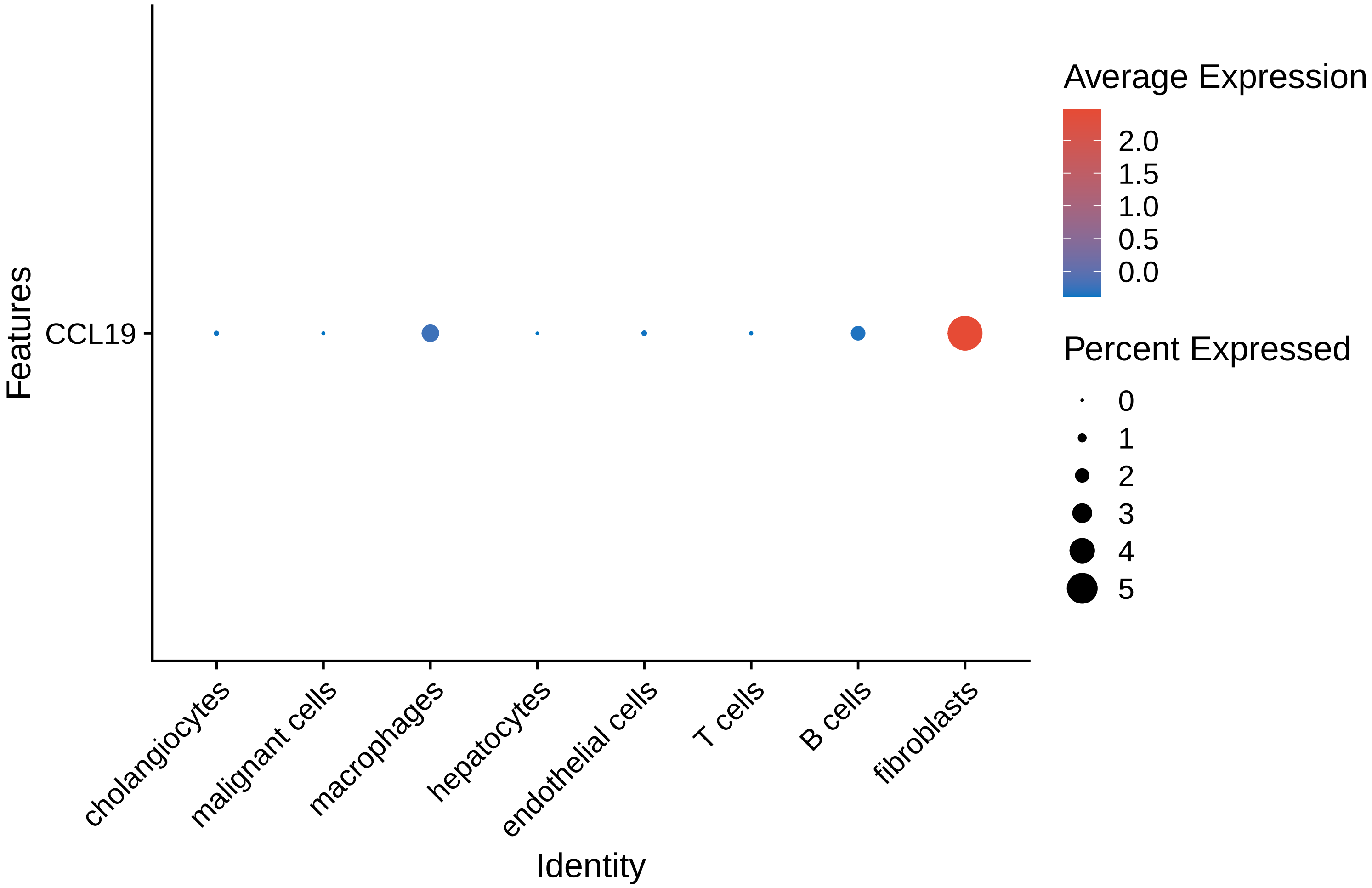

Average Expression
2.0
1.5
1.0
0.5
0.0
Percent Expressed
0
1
2
3
4
5
Features
CCL19
cholangiocytes
malignant cells
macrophages
hepatocytes
endothelial cells
T cells
B cells
fibroblasts
Identity
