# Supplementary material for: Downregulation of dermatopontin in cholangiocarcinoma cells suppresses CCL19 secretion of macrophages and immune infiltration
Source: J Cancer Res Clin Oncol. 2024 Feb 1;150(2):66. doi: 10.1007/s00432-023-05532-1 (PMC10834663; doi:10.1007/s00432-023-05532-1)
Supplement: Supplementary file 1 — Supplementary file1 (ZIP 153266 KB) [file 432_2023_5532_MOESM1_ESM.zip › Websites/survival curve of 9 genes/DPT.pdf]

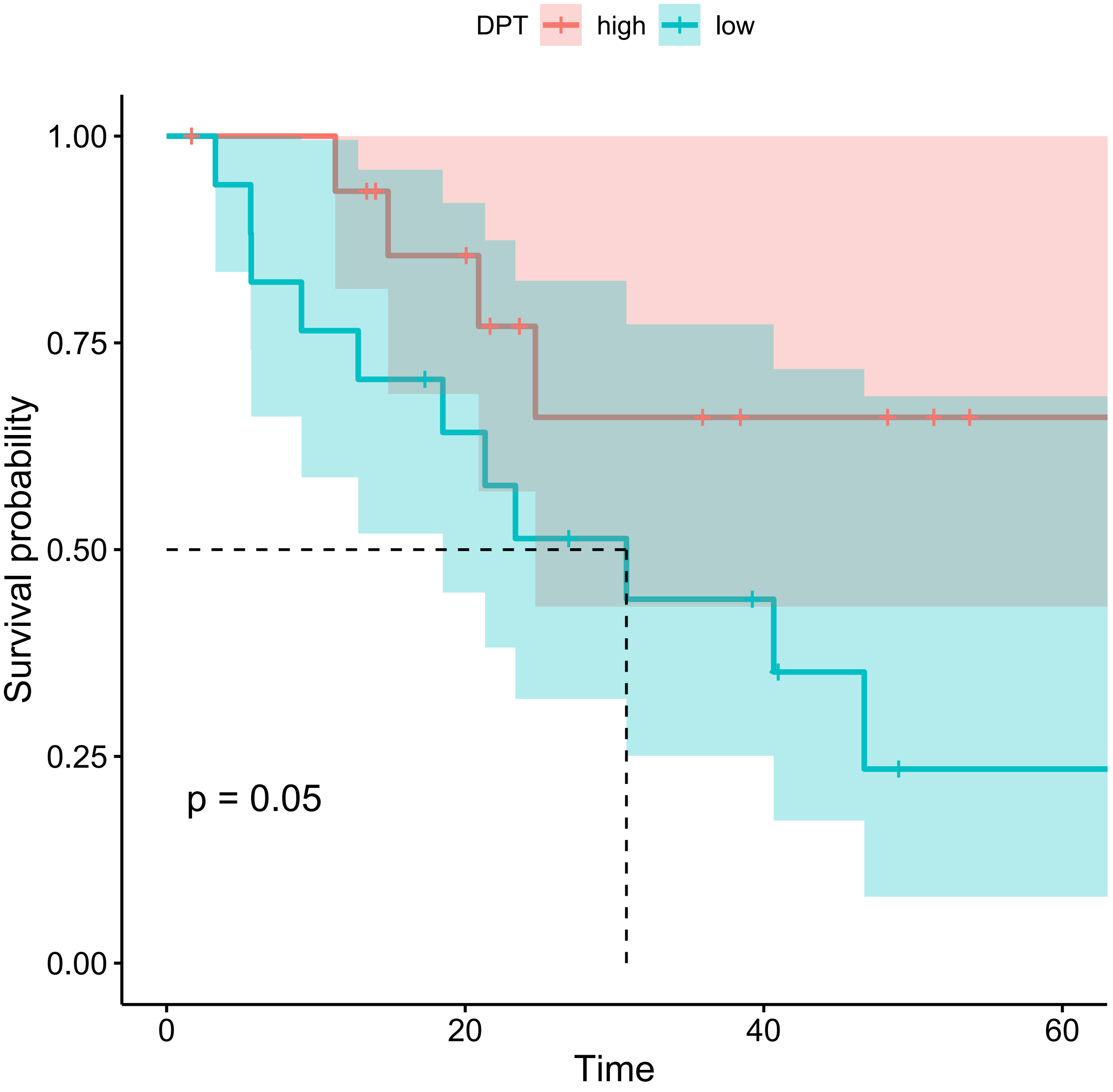
DPT
high
low
1.00
0.75
0.50
0.25
0.00
Survival probability
p = 0.05
0
20
40
60
Time
